# Design, Synthesis, and Evaluation of Novel 2*H*-Benzo[b][1,4]thiazin-3(4*H*)-one Derivatives as New Acetylcholinesterase Inhibitors

**DOI:** 10.3390/molecules27072121

**Published:** 2022-03-25

**Authors:** Sazan Haji Ali, Derya Osmaniye, Begüm Nurpelin Sağlık, Serkan Levent, Yusuf Özkay, Zafer Asım Kaplancıklı

**Affiliations:** 1 Department of Pharmaceutical Chemistry, Faculty of Pharmacy, Hawler Medical University, Erbil 44000, Iraq; sazandrug@gmail.com; 2 Department of Pharmaceutical Chemistry, Faculty of Pharmacy, Anadolu University, Eskişehir 26470, Turkey; dosmaniye@anadolu.edu.tr (D.O.); bnsaglik@anadolu.edu.tr (B.N.S.); serkanlevent@anadolu.edu.tr (S.L.); yozkay@anadolu.edu.tr (Y.Ö.); 3 Central Research Laboratory, Faculty of Pharmacy, Anadolu University, Eskişehir 26470, Turkey

**Keywords:** Alzheimer’s disease, thiadiazole, benzothiazine, acetylcholinesterase, molecular docking, antioxidant activity, blood–brain barrier permeability, cytotoxicity

## Abstract

Alzheimer’s disease (AD) is a slowly progressive neurodegenerative disease that causes dementia in people aged 65 and over. In the present study, a series of thiadiazole hybrid compounds with benzothiazine derivatives as acetylcholinesterase inhibitors were developed and evaluated for their biological activity. The AChE and BChE inhibition potentials of all compounds were evaluated by using the in vitro Ellman method. The biological evaluation showed that compounds **3i** and **3j** displayed significant inhibitory activity against AChE. Compounds **3i** and **3j** showed IC_50_ values of 0.027 µM and 0.025 µM against AChE, respectively. The reference drug donepezil (IC_50_ = 0.021 µM) also showed significant inhibition against AChE. Further docking simulation also revealed that these compounds (**3i** and **3j**) interacted with the active site of the enzyme similarly to donepezil. The antioxidant study revealed that compounds **3i** and **3j** exhibited greater antioxidant effects. An in vitro blood–brain barrier permeability study showed that compounds **3i** and **3j** are promising compounds against AD. The cytotoxicity study of compounds **3i** and **3j** showed non-cytotoxic with an IC_50_ value of 98.29 ± 3.98 µM and 159.68 ± 5.53 µM against NIH/3T3 cells, respectively.

## 1. Introduction

A silent epidemic in Europe, and worldwide in general, this is one of the most common types of dementia in people over the age of 65, known as Alzheimer’s disease (AD). According to the 2021 WHO global status report, the prevalence of AD and estimated cases with dementia will increase dramatically in the coming decades, from 55 million in 2019 to 139 million in 2050 [1]. AD is still an intricate health issue and remains an incurable disease in modern medicine. Unfortunately, this complication in treatment is due to many factors, such as erroneous differential diagnoses, unclear pathophysiology, and individual differences in symptoms; rarely do two people experience symptoms of dementia in the same way.

AD is a progressive, destructive neurodegenerative disorder. The main characteristics and clinical demonstrations of AD are cognitive impairment and memory loss, changes in mood and behavior, difficulty in performing familiar tasks, impairment in daily physical activities, in addition to a variety of neuropsychiatric symptoms [2]. The duration of the disease, during which the patient suffers from difficulty in performing daily activities to the final stage of memory loss and immobility, is protracted, about 8–10 years [3].

The etiology of AD, leading to the neurodegeneration and destruction of neurons, is explained by several factors. The aggregation of beta-amyloid proteins (Ab), the destruction of cholinergic neurons, neuroinflammation, mitochondrial damage, oxidative stress, and the degradation of tau proteins are the major predisposing factors for the progression of AD [4,5,6]. To date, the causes of neurodegeneration in AD patients are not well understood, so effective and curative drugs cannot yet be developed.

The prominent neuronal alteration in AD is a change in the level of cholinergic neurotransmitters such as acetylcholine ACh concentration in the cortex and hippocampus [7]. Studies on cholinergic neurons have shown that with disease progression, premature loss and severe damage to cholinergic neurons in the basal forebrain region can be observed. [8]. Consequently, the inhibition of the AChE enzyme to prevent the hydrolysis of ACh is the most important strategy in the treatment of AD. Currently, the clinically used AChEI drugs only have symptom-relieving effects and improve the quality of life of patients in mild to moderate stages of the disease. So far, there is no AChEI that can prevent the progression of the disease [9,10,11,12]. In addition, AChEIs do not show the same pharmacological effect in all AD patients, and to date, it remains unclear why some patients respond while others do not. [13,14]. According to some clinical studies regarding responders and non-responders to AChEIs, it was reported that the probability of response to donepezil in patients with temporal lobe atrophy is very low, while patients with a high level of an allele known as APOE-ε4 are more likely to show a response to donepezil [13,15,16,17]. On the other hand, oxidative stress, characterized by the production of free radical reactive oxygen species (ROS), is one of the predisposing factors for the progression of AD. The overproduction of ROS plays an important role in the age-related progression of neurodegeneration and cognitive impairment [6,18].

**Figure 1:**
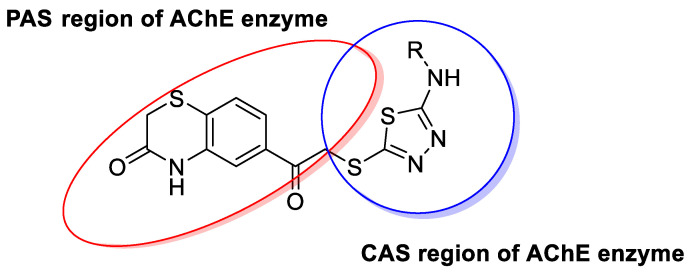
Acetylcholine esterase enzyme-binding sites.

Compounds with dual binding affinity to both active binding sites of the AChE enzyme, such as donepezil, have an excellent AChE inhibition profile [19,20]. In continuation of our previous research [21], in this work, we designed and synthesized a series of donepezil-based molecules with an evaluation of their AChE inhibitor activity in comparison to donepezil, as shown in Figure 1.

In drug development, heterocyclic rings are usually used as the main constituent of the molecular structure. In medicinal chemistry, molecules with nitrogen and sulfur as functional groups have shown a variety of biological activities [22]. Five membered 1,3,4-thiadiazole rings have demonstrated various biological activities, which includes acetylcholinesterase inhibitors [23], antibacterial [24,25], antifungal [26,27], anti-cancer [28,29], antioxidant [30], analgesic, and anti-inflammatory activity [31].

In the present study, the hybridization of two bioactive molecules, thiadiazole with benzothiazine, as a single biologically active moiety was designed. The newly combined molecules were tested for their biological activity as an acetylcholinesterase inhibitor in the treatment of Alzheimer’s disease.

## 2. Results and Discussion

### 2.1. Chemistry

**Scheme 1:**
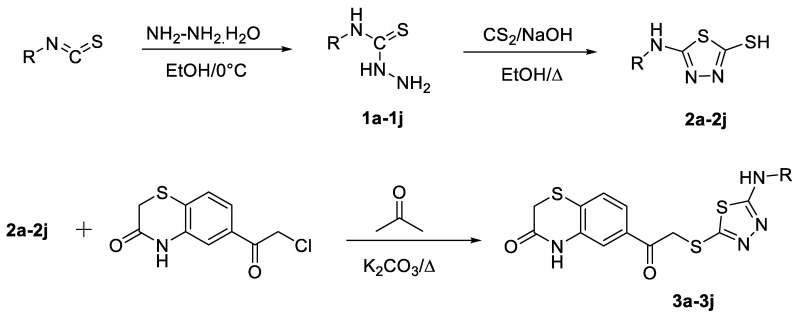
Synthesis pathway of compounds (**3a**–**3j**).

Compounds **3a**–**3j** were synthesized in several steps, as shown in Scheme 1. In addition, the formulas of the compounds obtained are presented in Table 1. Initially, the synthesis was started with the derivatives of isothiocyanate in reaction with hydrazine hydrate. The series of **1a**–**1j** compounds were obtained and, in another step, reacted with CS_2_ to achieve compounds **2a**–**2j**. Finally, the target compounds **3a**–**3j** were obtained via a nucleophilic substitution reaction between thiadiazole ring derivatives **2a**–**2j** and a 6-(2-chloroacetyl)-2*H*-benzo[b][1,4]thiazin-3(4*H*)-one molecule. The structures of the achieved compounds were verified using spectroscopic methods, namely IR, ^1^H-NMR, ^13^C-NMR, and HRMS (Supplementary Data).

### 2.2. Cholinesterase Enzymes Inhibition Assay

The inhibitory activities of all of the obtained chalcone derivatives (**3a**–**3j**) against cholinesterase enzymes were evaluated using the previously described in vitro modified Ellman’s spectrophotometric method [32,33,34,35,36,37,38,39,40].

**Figure 2:**
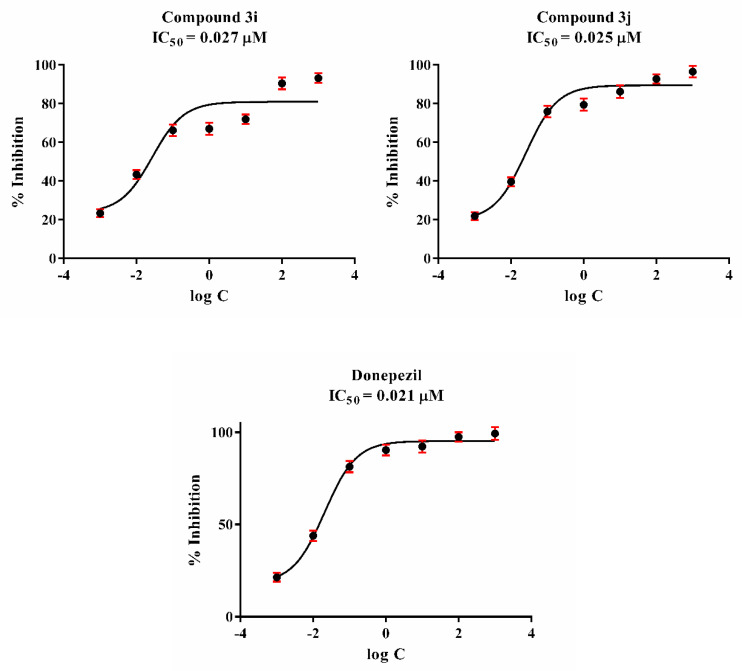
IC_50_ graphs of compounds **3i** and **3j** and donepezil on AChE enzyme (the graphs were formed using GraphPad Prism Version 6 via regression analyses).

The assay was completed in two steps. The first step was achieved by means of all the chalcones (**3a**–**3j**) and reference agents, namely donepezil and tacrine, at concentrations of 1000 and 100 µM. The enzyme activity results of the first step are presented in Table 2. Next, the selected compounds (**3i** and **3j**) that displayed more than 50% inhibitory activity at concentrations of 1000 and 100 µM were further tested, along with the reference agents, at concentrations of 10 to 0.001 µM. The IC_50_ values of the test compounds and reference agents are presented in Figure 2.

As a result of the enzyme inhibition test, it was observed that all compounds were more effective against AChE. None of the compounds exhibited greater than 50% inhibitory activity against BChE in the first step. However, especially compounds with an aromatic substituent showed inhibitory activity comparable to that of donepezil against AChE. Compounds **3i** and **3j** passed the second step of the enzyme activity assay, and their IC_50_ values were calculated by performing an enzyme inhibition study at concentrations of 10 to 0.001 µM. The IC_50_ values of compounds **3i** and **3j** were calculated as 0.027 and 0.025 µM, respectively.

When the building activity relations were examined, derivatives containing aromatic substitutions were found to be more active. Derivatives with an electron-withdrawing substituent in the fourth position of the phenyl ring were more active than the non-substituted phenyl ring. In this case, it can be said that the substituent in the fourth position was necessary for the activity. One of the derivatives containing substituents in this position carried methoxy and the other chlorine substituents. It was observed that the chlorine-bearing substituent was more active. Molecular docking studies were carried out to elucidate the reason for this.

### 2.3. DPPH Free Radical Scavenging Antioxidant Activity

This activity method is a method based on measuring the scavenging effects of the DPPH• (1,1-Diphenyl-2-picrylhydrazil) radical, which is a stable organic nitrogen radical of antioxidants. One of the few stable organic nitrogen radicals, the DPPH• radical is dark violet in color. This method, which is based on the scavenging of the DPPH radical via antioxidants, is a redox reaction. When this radical interacts with hydrogen donors, it is reduced to hydrazine. The dark purple color of the methanolic DPPH• solution turns yellow with the addition of antioxidants, and the change in absorbance is measured spectrophotometrically. More illumination is determined by a greater reduction in the absorbance of the reaction mixture and indicates higher radical scavenging capacity [41,42].

**Figure 3:**
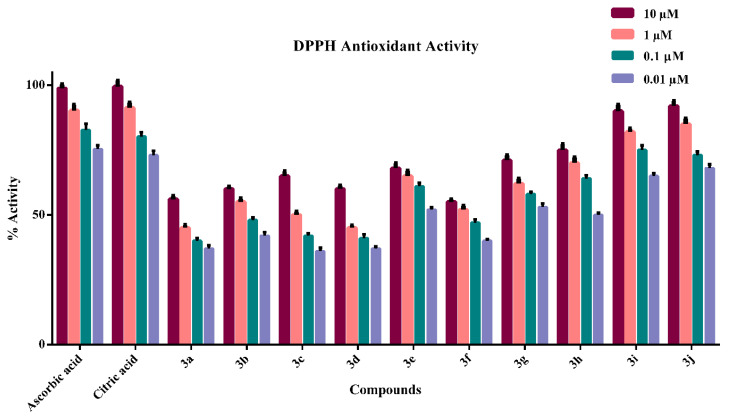
% DPPH antioxidant activity of the synthesized compounds **3a**–**3j** and reference agents.

For the DPPH free radical scavenging activity test, test compounds were prepared at 10 µM, 1 µM, 0.1 µM, and 0.01 µM concentrations, as shown in Figure 3. By using the absorbance changes determined because of the spectroscopic measurement, the % DPPH free radical scavenging activities of the synthesized compounds and reference materials (ascorbic acid and citric acid) were determined. The % antioxidant activities of all tested compounds were calculated based on the control. Among the test compounds, all compounds at a concentration of 10 µM showed more than 50% antioxidant activity. Compounds **3i** and **3j** showed inhibition of 90.00% ± 2.40; 92.00% ± 1.80, respectively, at this concentration. They showed inhibition of 82.00% ± 1.20; 85.00% ± 2.00, respectively, at 1 µM concentration. Compounds **3i** and **3j** showed inhibition of 75.00% ± 1.80; 73.00% ± 1.40, respectively, at a concentration of 0.1 µM. They showed inhibition of 65.00% ± 1.10; 68.00% ± 1.50, respectively, at a concentration of 0.01 µM. Compounds **3i** and **3j** were observed to have a higher antioxidant activity compared to other obtained compounds. As a result of these findings, it was revealed that compounds **3i** and **3j** may have the potential effects on patients suffering from AD.

### 2.4. In Vitro BBB Permeability Assay

As it is known, the ability of drugs to be used in diseases related to the central nervous system to pass the blood–brain barrier is an indispensable feature. These properties of the synthesized compounds were estimated using physicochemical parameters. Additionally, according to these parameters, it is seen that they have physicochemical properties that can pass the BBB. To verify the validity of these estimated data, in vitro PAMPA tests were performed on the most active derivatives. Additionally, the results are presented in Table 3. Compounds **3i** and **3j** was found to have high BBB permeability. This valuable evidence once again proved that compounds **3i** and **3j** are promising compounds against AD.

### 2.5. Cytotoxicity Assay

Compounds **3i** and **3j** exhibited potent AChE inhibition profiles and were further tested for toxicity using the MTT assay in the NIH/3T3 cell line; the IC_50_ values of these compounds were calculated. Compound **3i** and **3j** showed non-cytotoxic with an IC_50_ value of 98.29 ± 3.98 µM and 159.68 ± 5.53 µM against NIH/3T3 cells, respectively. This result suggests that the compounds did not show cytotoxic activity.

### 2.6. Molecular Docking Studies

**Figure 4:**
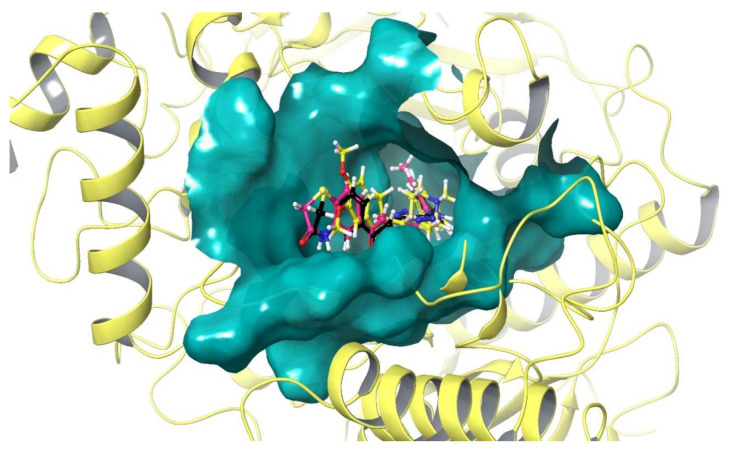
The three-dimensional superimposition poses of compounds **3i**, **3j** and donepezil in the active region of human AChE enzyme (PDB ID: 4EY7). The important residues in the active site and related compounds are presented by tube model and colored with turquoise, pink, black and yellow, respectively.

**Figure 5:**
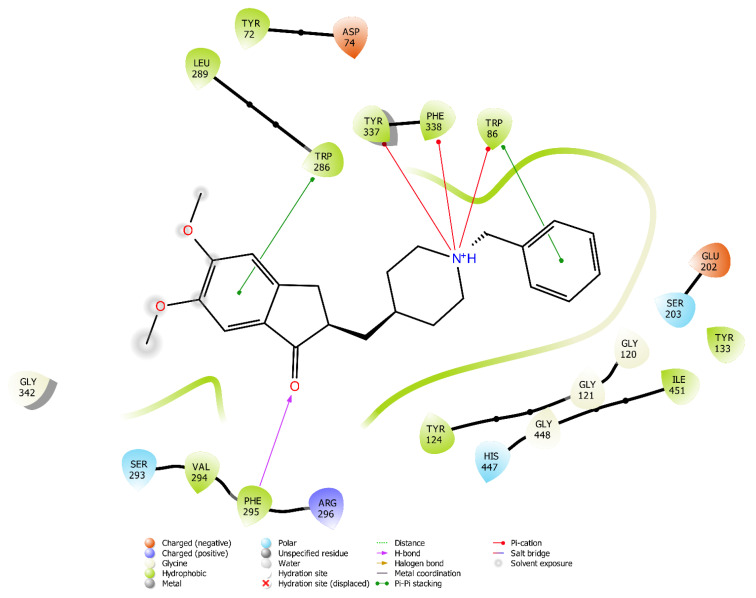
The two-dimensional interacting mode of donepezil in the active region of AChE (PDB ID: 4EY7).

**Figure 6:**
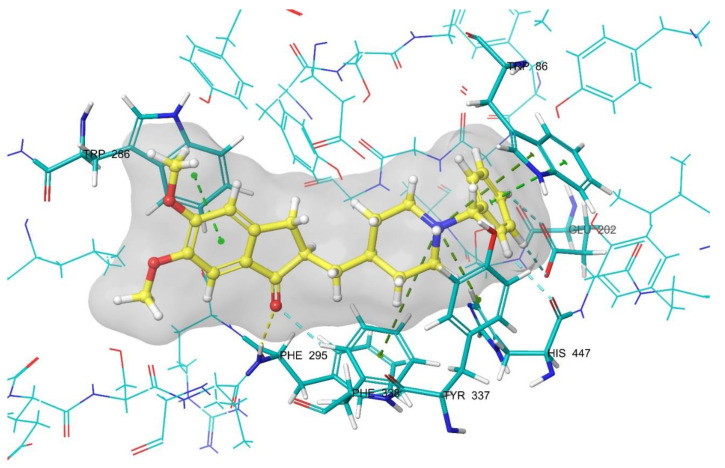
The three-dimensional interacting mode of donepezil in the active region of AChE (PDB ID: 4EY7). The inhibitor and important residues in the active site of enzyme are presented by tube model colored with red and turquoise, respectively.

Molecular docking studies were performed to verify the compounds’ inhibition capabilities in silico. For this purpose, the crystal structure of human acetylcholine esterase enzyme (PDB ID: 4EY7) [19] was retrieved from the Protein Data Bank server (www.pdb.org) (accessed on 1 January 2022). The resulting docking poses are presented in Figure 4, Figure 5 and Figure 6.

Figure 4 shows the localization of all active compounds (**3i** and **3j**) and donepezil to the enzyme active site. All compounds were localized to the enzyme active site.

Figure 5 and Figure 6 show that the indanone ring of donepezil exhibited one π–π interaction with the indole ring of amino acid Trp286. The carbonyl group of the indanone formed a hydrogen bond with the amine group of Phe295, and this group formed an aromatic hydrogen bond with phenyl of Phe338. The nitrogen of piperidine ring exhibited three π–π interactions with the phenyl ring of Phe338, Tyr337, and the indole ring of Trp86. The phenyl ring of the benzyl group attached to the piperidine ring exhibited one π–π interaction and three aromatic hydrogen bonds. While the π–π interaction occurred with the indole ring of Trp86, the aromatic hydrogen bonds were formed between the carbonyl group of His447, the carbonyl group of Glu202, and the hydroxyl group of Glu202.

**Figure 7:**
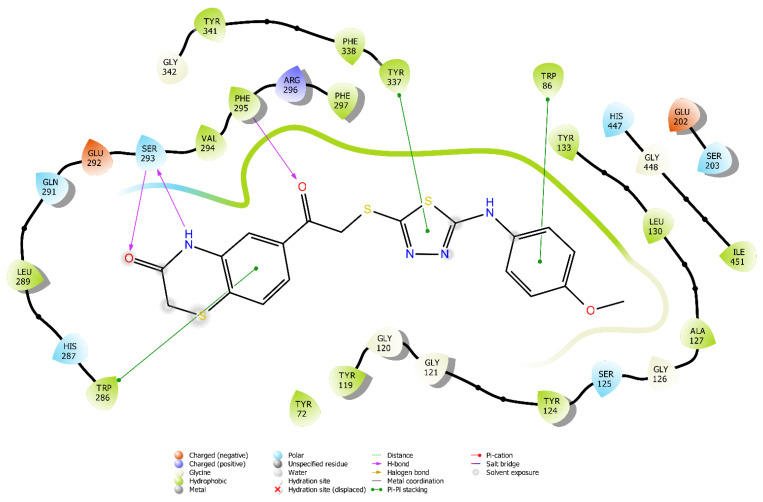
The two-dimensional interacting mode of compound **3i** in the active region of AChE (PDB ID: 4EY7).

**Figure 8:**
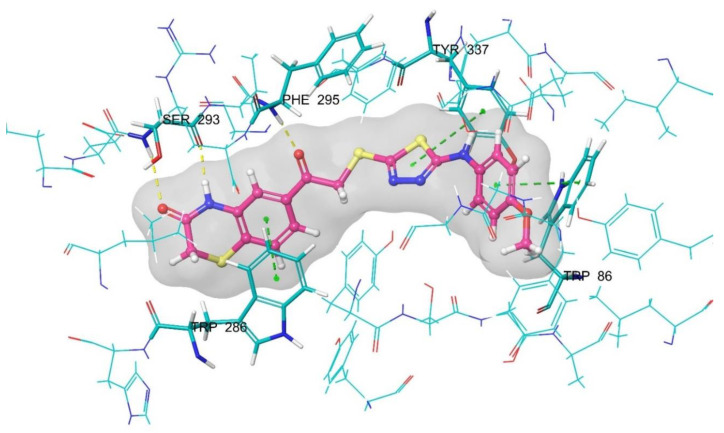
The three-dimensional interacting mode of compound **3i** in the active region of AChE (PDB ID: 4EY7). The inhibitor and important residues in the active site of enzyme are presented by tube model colored with pink and turquoise, respectively.

Figure 7 and Figure 8 show that the 2*H*-benzo[b][1,4]thiazin-3(4*H*)-one ring of compound **3i** exhibited π–π interactions with the indole ring of amino acid Trp286. The carbonyl group and amino group of 2*H*-benzo[b][1,4]thiazin-3(4*H*)-one ring formed two hydrogen bonds with the hydroxy group of Ser293 and carbonyl group of Ser293. The carbonyl group of compound **3i** formed a hydrogen bond with the hydroxy group of Phe295. The thiadiazol ring of compound **3i** formed a π–π interaction with the phenyl ring of Tyr337. The phenyl ring of the compound **3i** exhibited π–π interactions with the indole ring of Trp86.

**Figure 9:**
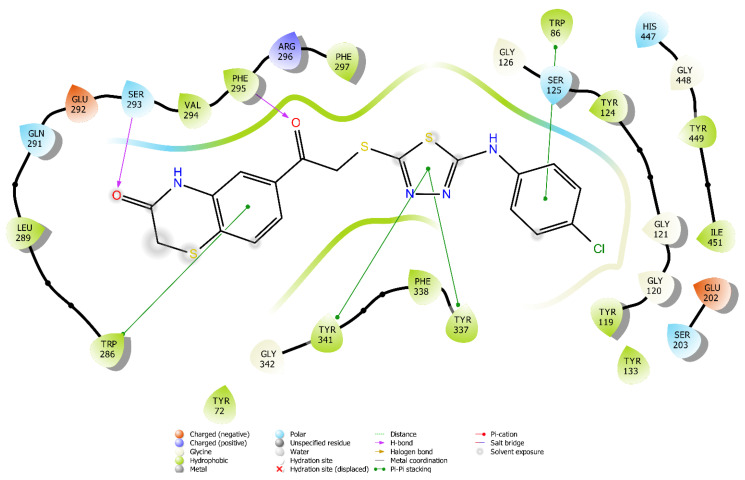
The two-dimensional interacting mode of compound **3j** in the active region of AChE (PDB ID: 4EY7).

**Figure 10:**
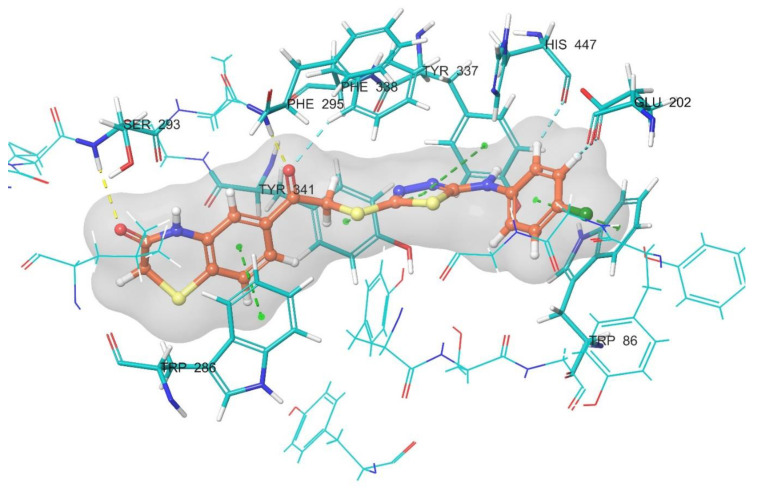
The three-dimensional interacting mode of compound **3j** in the active region of AChE (PDB ID: 4EY7). The inhibitor and important residues in the active site of enzyme are presented by tube model colored with purple and turquoise, respectively.

Figure 9 and Figure 10 show that the 2*H*-benzo[b][1,4]thiazin-3(4*H*)-one ring of compound **3j** exhibited a π–π interaction with the indole ring of amino acid Trp286. The carbonyl group of 2*H*-benzo[b][1,4]thiazin-3(4*H*)-one ring formed a hydrogen bond with the amine group of Ser293. The carbonyl group of compound **3j** formed a hydrogen bond with the amino group of Phe295, and this group formed an aromatic hydrogen bond with phenyl rings of Phe338. The thiadiazol ring of compound **3j** formed two π–π interactions with phenyl rings of Tyr341 and Tyr337. The phenyl ring of compound **3j** formed a π–π interaction with the indole ring of Trp86. Additionally, this phenyl ring formed two aromatic hydrogen bonds with carbonyl groups of His447 and Glu202.

In the light of the above information, it is seen that the aromatic ring is required for the thiadiazole ring. According to the activity results, active derivatives carry aromatic substituents. This seems to be due to the interaction between aromatic substituents and Trp86 in the CAS region of the enzyme. In addition, if it carries halogen on the aromatic structure here, it also establishes aromatic hydrogen bonds with Glu202 and His447. These bonds are also made by donepezil, and their properties show selectivity against AChE.

## 3. Materials and Methods

### 3.1. Chemistry

#### 3.1.1. General

All reagents were purchased from commercial suppliers and were used without further purification. Melting points (M.p.) were determined using the Mettler Toledo-MP90 Melting Point System and were uncorrected. A ^1^H-NMR (nuclear magnetic resonance) Bruker DPX 300 FT-NMR spectrometer and a ^13^C-NMR, Bruker DPX 75 MHz spectrometer (Bruker Bioscience, Billerica, MA, USA) were used. A total of 10 mg of compound was weighed and dissolved in 600 µL of DMSO-*d*_6_. In this way, the NMR procedure was carried out. Mass spectra were recorded on an LCMS-IT-TOF (Shimadzu, Kyoto, Japan) using ESI. In total, 10 mg of compound was weighed and dissolved in 1500 µL of MeOH. In this way, the HRMS procedure was carried out.

#### 3.1.2. Synthesis of Hydrazine Carbosulfanylamide Derivatives (**1a**–**1j**)

Isothiocyanate derivatives (0.007 mol) and an excess of hydrazine hydrate were dissolved in ethanol (50 mL) and mixed at 0 °C. At the end of the reaction, ethanol was evaporated, and the precipitated product was purified via recrystallization.

#### 3.1.3. Synthesis of 5-(R-amino)-1,3,4-thiadiazole-2-sulfanyl Derivatives (**2a**–**2j**)

Compounds **1a**–**1j** (0.006 mol) were dissolved in absolute ethanol (20 mL). To the reaction mixture, 1 equivalent of NaOH and 1.2 equivalent of CS_2_ were added. The reaction mixture was refluxed for 8–10 h and followed up by TLC. The finished reaction mixture was acidified with 20% HCl, and the precipitates were filtered and purified through recrystallization in ethanol.

*5-(Ethylamino)-1,3,4-thiadiazole-2-thiol* (**2a**)

Yield: 90%, ^1^H-NMR (300 MHz, DMSO-*d*_6_): δ = 1.08 (3H, t, *J* = 7.1, -CH_3_), 3.1–3.36 (2H, q, *J*_1_ = 5.4, *J*_2_= 7.2, -CH_2_), 7.52 (1H, s, thiazin-NH-), 13.27 (1H, s, SH).

*5-(Propylamino)-1,3,4-thiadiazole-2-thiol* (**2b**)

Yield: 85%, ^1^H-NMR (300 MHz, DMSO-*d*_6_): δ = 0.84 (2H, m, -CH_2_), 1.4–1.52 (3H, t, *J* = 7.1, -CH_3_) 3.05–3.08 (2H, t, *J*_1_ = 7.1, -CH_2_), 7.56 (1H, s, thiazin-NH-), 13.26 (1H, s, SH).

*5-(Isopropylamino)-1,3,4-thiadiazole-2-thiol* (**2c**)

Yield: 92%, ^1^H-NMR (300 MHz, DMSO-*d*_6_): δ = 1.12 (6H, s, -2CH_3_) 3.61 (1H, m, -CH), 7.49 (1H, s, thiazin-NH-), 13.26 (1H, s, SH).

5-(Butyllamino)-1,3,4-thiadiazole-2-thiol (**2d**)

Yield: 89%, ^1^H-NMR (300 MHz, DMSO-*d*_6_): δ = 0.84 (3H, t, *J* = 7.15 -CH_3_), 1.26–1.33 (2H, m, -CH_2_), 1.45–1.49 (2H, m, -CH_2_), 3.05–3.43 (2H, s, -CH_2_), 7.57 (1H, s, thiazin-NH-), 13.26 (1H, s, SH).

*5-(Isobutylamino)-1,3,4-thiadiazole-2-thiol* (**2e**)

Yield: 92%, ^1^H-NMR (300 MHz, DMSO-*d*_6_): δ = 0.86 (6H, d, *J* = 6.6 -2CH_3_), 1.75–1.88 (1H, m, -CH), 2.95–2.99 (2H, d, *J* = 6.1 -CH_2_), 7.77 (1H, s, thiazin-NH-), 13.24 (1H, s, SH).

*5-(Cyclohexylamino)-1,3,4-thiadiazole-2-thiol* (**2f**)

Yield: 88%, ^1^H-NMR (300 MHz, DMSO-*d*_6_): δ = 1.17–1.29 (4H, m, cyclohexane), 1.51–1.66 (2H, m, cyclohexane), 1.86–1.88 (4H, m, cyclohexane), 1.9 (1H, m, cyclohexane), 7.49 (1H, s, thiazin-NH-), 13.24 (1H, s, SH).

*5-(Phenylamino)-1,3,4-thiadiazole-2-thiol* (**2g**)

Yield: 91%, ^1^H-NMR (300 MHz, DMSO-*d*_6_): δ = 6.97 (1H, t, J = 7.4, phenyl), 7.02 (2H, m, phenyl), 7.42 (2H, dd, *J*_1_ = 1.75, *J*_2_ = 8.1, phenyl), 10.23 (1H, s, thiazin-NH-), 13.67 (1H, s, SH).

*5-(p-Tolylamino)-1,3,4-thiadiazole-2-thiol* (**2h**)

Yield: 91%, ^1^H-NMR (300 MHz, DMSO-*d*_6_): δ = 2.5 (3H, s, -CH_3_), 7.1–7.3 (4H, dd, *J*_1_ = 1.7, *J*_2_ = 7.79, phenyl), 10.06 (1H, s, thiazin-NH-), 13.56 (1H, s, SH).

*5-(4-Methoxyphenylamino)-1,3,4-thiadiazole-2-thiol* (**2i**)

Yield: 87%, ^1^H-NMR (300 MHz, DMSO-*d*_6_): δ = 3.7 (3H, s, -CH_3_), 6.89–7.33 (4H, dd, *J*_1_ = 1.7, *J*_2_ = 7.79, phenyl), 9.98 (1H, s, thiazin-NH-), 13.39 (1H, s, SH).

*5-((4-Chlorophenyl)amino)-1,3,4-thiadiazole-2-thiol* (**2j**)

Yield: 84%, ^1^H-NMR (300 MHz, DMSO-*d*_6_): δ = 7.35–7.45 (4H, dd, *J*_1_ = 1.69, *J*_2_ = 7.71, phenyl), 10.4 (1H, s, thiazin-NH-), 13.72 (1H, s, SH).

#### 3.1.4. Synthesis of Target Compounds (**3a**–**3j**)

The thiadiazole derivatives (**2a**–**2j**) (0.001 mol) and 6-(2-chloroacetyl)-2H-benzo[b] [1,4] thiazin-3(4H)-one (0.001 mol) were dissolved in acetone (20 mL) with the addition of 0.001 mol of K_2_CO_3_, and the mixture was refluxed overnight. After the completion of the reaction, the mixture was cooled, and precipitated product was filtered and purified using the recrystallization method in ethanol.

*6-(2-((5-(Ethylamino)-1,3,4-thiadiazol-2-yl)sulfanyl)acetyl)-2H-benzo[b][1,4]thiazin-3(4H)-one* (**3a**)

Yield: 90%, M.P.: 196.9–199 °C. IR (cm^−1^ bands): 3190 (N-H), 3093 (C-H), 1670 (C=O), 1394 (C-O); ^1^H-NMR (300 MHz, DMSO-*d*_6_): δ = 1.13 (3H, t, *J* = 7.18, -CH_3_), 3.21–3.25 (2H, q, *J*_1_ = 5.39, *J*_2_ = 7.15, -CH_2_), 3.56 (2H, s, 1,4-thiazine-3(4*H*)-one), 4.72 (2H, s, Sulfanylacetyl-H), 7.48–7.53 (2H, m, benzothiazine -H), 7.63–7.66 (1H, dd, *J*_1_ = 1.77, *J*_2_ = 8.13 benzothiazine, -H), 7.77–7.80 (1H, t, *J*_1_ = 5.26 Hz, propylamin-NH), 10.77 (1H, s, thiazin-NH-); ^13^C-NMR (75 MHz, DMSO-*d*_6_): δ = 11.81, 22.19, 28.87, 41.81, 46.83, 123.60, 124.64, 126.79, 128.98, 134.11, 138,09, 165.28, 192.77. HRMS (*m*/*z*): [M + H]^+^ calcd for C_14_ H_14_ N_4_ O_2_ S_3_: 367.0352; found: 367.0370.

*6-(2-((5-(Propylamino)-1,3,4-thiadiazol-2-yl)sulfanyl)acetyl)-2H-benzo[b][1,4]thiazin-3(4H)-one* (**3b**)

Yield: 89%, M.P.: 191.3–193.8 °C. IR (cm^−1^ bands): 3226 (N-H), 3095 (C-H), 1695 (C=O), 1369 (C-O); ^1^H-NMR (300 MHz, DMSO-*d*_6_): δ = 0.9 (3H, t, *J* = 7.93, -CH_3_), 1.50–1.57 (2H, m, -CH_2_), 3.1–3.2 (2H, q, -CH_2_), 3.56 (2H, s, 1,4-thiazine-3(4*H*)-one), 4.72 (2H, s, Sulfanylacetyl-H), 7.48–7.53 (2H, m, benzothiazine -H), 7.63–7.66 (1H, dd, *J*_1_ = 1.79, *J*_2_ = 8.1 benzothiazine, -H), 7.79–7.83 (1H, t, *J*_1_ = 5.42 Hz, propylamin-NH), 10.76(1H, s, thiazin-NH-); ^13^C-NMR (75 MHz, DMSO-*d*_6_): δ = 11.81, 22.19, 28.87, 41.81, 46.83, 123.60, 124.64, 126.79, 128.98, 134.11, 138,09, 165.28, 192.77. HRMS (*m*/*z*): [M + H]^+^ calcd for C_15_ H_16_ N_4_ O_2_ S_3_: 381.0508; found: 381.0513.

*6-(2-((5-(Isopropylamino)-1,3,4-thiadiazol-2-yl)sulfanyl)acetyl)-2H-benzo[b][1,4]thiazin-3(4H)-one* (**3c**)

Yield: 92%, M.P.: 212.5–214.4 °C. IR (cm^−1^ bands): 3219 (N-H), 2983 (C-H), 1699 (C=O), 1369 (C-O); ^1^H-NMR (300 MHz, DMSO-*d*_6_): δ = 1.14–1.16 (6H, d, *J* = 6.44, CH_3-_ isopropyl), 3.68–3.79 (1H, m, -CH), 3.56 (2H, s, 1,4-thiazine-3(4*H*)-one), 4.72 (2H, s, Sulfanylacetyl-H), 7.48–7.53 (2H, m, benzothiazine -H), 7.63–7.66 (1H, dd, *J*_1_ = 1.81*, J*_2_ = 8.13 benzothiazine, -H), 7.70–7.72 (1H, d, *J*_1_ = 7.1 Hz, isopropylamin-NH), 10.76 (1H, s, thiazin-NH); ^13^C-NMR (75 MHz, DMSO-*d*_6_): δ = 22.57, 28.86, 41.78, 46.98,116.54, 123.59, 126.85, 127.88, 134.06, 138,09, 140.66, 148.77, 165.27, 169.19, 192.77. HRMS (*m*/*z*): [M + H]^+^ calcd for C_15_ H_16_ N_4_ O_2_ S_3_: 381.0508; found: 381.0529.

*6-(2-((5-(Butylamino)-1,3,4-thiadiazol-2-yl)sulfanyl)acetyl)-2H-benzo[b][1,4]thiazin-3(4H)-one* (**3d**)

Yield: 70%, M.P.: 177–180 °C. IR (cm^−1^ bands): 3240 (N-H), 3184 (C-H), 1666 (C=O), 1398 (C-O); ^1^H-NMR (300 MHz, DMSO-*d*_6_): δ = 0.8–0.9 (3H, t, *J* = 7.3 Hz -CH_3_), 1.27–1.35 (2H, m, methylene), 1.46–1.53 (2H, m, methylene), 1.02–1.07 (2H, t, *J* = 6.99, methylamine-CH_2_), 3.56 (2H, s, 1,4-thiazine-3(4*H*)-one), 4.99 (2H, s, Sulfanylacetyl-H), 7.5–7.54 (2H, m, benzothiazine -H), 7.65–7.66 (1H, dd, *J*_1_ = 1.8*, J*_2_ = 8.13 benzothiazine, -H), 7.94 (1H, s, butylamine-NH), 10.76 (1H, s, thiazin-NH-). HRMS (*m*/*z*): [M + H]^+^ calcd for C_16_ H_18_ N_4_ O_2_ S_3_:395.0665; found: 395.0659.

*6-(2-((5-(Isobutylamino)-1,3,4-thiadiazol-2-yl)sulfanyl)acetyl)-2H-benzo[b][1,4]thiazin-3(4H)-one* (**3e**)

Yield: 92%, M.P.: 194.1–195.8 °C. IR (cm^−1^ bands): 3242 (N-H), 3196 (C-H), 1668 (C=O), 1398 (C-O); ^1^H-NMR (300 MHz, DMSO-*d*_6_): δ = 0.87–0.89 (6H, d, *J* = 6.67 Hz, 2CH_3-_ isobutyl), 1.79–1.88 (1H, m, CH-isobutyl), 3.02–3.06 (2H, dd, *J*_1_ = 1.01, *J*_2_ = 12.44, CH_2_-isobutyl), 3.56 (2H, s, 1,4-thiazine-3(4*H*)-one), 4.71 (2H, s, Sulfanylacetyl-H), 7.48–7.53 (2H, m, benzothiazine -H), 7.63–7.66 (1H, dd, *J*_1_ = 1.81*, J*_2_ = 8.13 benzothiazine, -H), 7.81–7.85 (1H, t, *J*_1_ = 5.59 Hz, isobutylamin-H), 10.76 (1H, s, thiazin-NH-); ^13^C-NMR (75 MHz, DMSO-*d*_6_): δ = 20.50, 27.95, 28.87, 41.79, 52.65, 116.56, 123.60, 126.86, 127.89, 134.07, 138,09, 165.28, 170.38, 192.78. HRMS (*m*/*z*): [M + H]+ calcd for C_16_ H_18_ N_4_ O_2_ S_3_: 395.0665; found: 395.0665.

*6-(2-((5-(Cyclohexylamino)-1,3,4-thiadiazol-2-yl)sulfanyl)acetyl)-2H-benzo[b][1,4]thiazin-3(4H)-one* (**3f**)

Yield: 88%, M.P.: 229–232 °C. IR (cm^−1^ bands): 3242 (N-H), 3221 (C-H), 1674 (C=O), 1388 (C-O); ^1^H-NMR (300 MHz, DMSO-*d*_6_): δ = 1.14–1.35 (4H, m, cyclohexane), 1.52–1.69 (2H, m, cyclohexane), 1.91–1.93 (4H, m, cyclohexane), 2.7 (1H, m, cyclohexane), 3.56 (2H, s, 1,4-thiazine-3(4*H*)-one), 4.70 (2H, s, Sulfanylacetyl-H), 7.48–7.53 (2H, m, benzothiazine -H), 7.62–7.66 (1H, dd, *J*_1_ = 1.83*, J*_2_ = 8.13 benzothiazine, -H), 7.73–7.75 (1H, t, *J*_1_ = 7.29 Hz, isobutylamin-H), 7.95 (1H, s, benzothiazine, -H), 10.76 (1H, s, thiazin-NH); ^13^C-NMR (75 MHz, DMSO-*d*_6_): δ = 24.65, 25.65, 28.86, 32.44, 41.76, 53.91, 116.55, 123.60, 126.85, 127.88, 134.08, 138,09, 165.28, 169.22, 192.80. HRMS (*m*/*z*): [M + H]+ calcd for C_18_ H_20_ N_4_ O_2_ S_3_: 421.0821; found: 421.0821.

*6-(2-((5-(Phenylamino)-1,3,4-thiadiazol-2-yl)sulfanyl)acetyl)-2H-benzo[b][1,4]thiazin-3(4H)-one* (**3g**)

Yield: 91%, M.P.: 243–245 °C. IR (cm^−1^ bands): 3197 (N-H), 3074 (C-H), 1662 (C=O), 1398 (C-O); ^1^H-NMR (300 MHz, DMSO-*d*_6_): δ = 3.56 (2H, s, 1,4-thiazine-3(4*H*)-one), 4.87 (2H, s, Sulfanylacetyl-H), 6.96–7.0 (1H, t, J = 7.34, phenyl), 7.29–7.35 (2H, t, *J* = 15.91, phenyl), 7.50–7.56 (2H, d, *J* = 6.83, benzothiazine -H), 7.67–7.70 (2H, dd, *J*_1_ *=* 1.79, *J*_2_ = 8.14, phenyl), 7.94 (1H, s, benzothiazine, -H)), 10.37 (1H, s, phenylamine-H), 10.78 (1H, s, thiazin-NH-); ^13^C-NMR (75 MHz, DMSO-*d*_6_): δ = 28.86, 41.61, 116.53, 117.81, 122.46, 123.59, 126.96, 127.94, 129.58, 134.07, 138,12, 140.79, 152.34, 165.29, 165.32, 192.57. HRMS (*m*/*z*): [M + H]^+^ calcd for C_18_ H_14_ N_4_ O_2_ S_3_: 415.0352; found: 415.0357.

*6-(2-((5-(p-Tolylamino)-1,3,4-thiadiazol-2-yl)sulfanyl)acetyl)-2H-benzo[b][1,4]thiazin-3(4H)-one* (**3h**)

Yield: 95%, M.P.: 245–247.7 °C. IR (cm^−1^ bands): 3244 (N-H), 3192 (C-H), 1662 (C=O), 1400 (C-O); ^1^H-NMR (300 MHz, DMSO-*d*_6_): δ = 2.24 (3H, s, methyl), 3.56 (2H, s, 1,4-thiazine-3(4*H*)-one), 4.85 (2H, s, Sulfanylacetyl-H), 7.11–7.14 (2H, d, *J* = 8.3, phenyl), 7.40–7.43 (2H, d, *J* = 8.48, phenyl), 7.66–7.69 (2H, dd, *J*_1_ = 1.79, *J*_2_ = 8.14 benzothiazine), 7.94 (1H, s, benzothiazine-H), 10.26 (1H, s, phenylamine-H), 10.77 (1H, s, thiazin-NH-); ^13^C-NMR (75 MHz, DMSO-*d*_6_): δ = 20.78, 28.86, 41.62, 116.52, 117.94, 123.58, 126.95, 127.93, 129.96, 131.46, 134.07, 138,11, 138.42, 151.83, 165.29, 165.56, 192.58. HRMS (*m*/*z*): [M + H]^+^ calcd for C_19_ H_16_ N_4_ O_2_ S_3_: 429.0508; found: 429.0509.

*6-(2-((5-((4-Methoxyphenyl)amino)-1,3,4-thiadiazol-2-yl)sulfanyl)acetyl)-2H-benzo[b][1,4]thiazin-3(4H)-one* (**3i**)

Yield: 96%, M.P.: 217–220 °C. IR (cm^−1^ bands): 3234 (N-H), 3197 (C-H), 1681 (C=O), 1244 (C-O); ^1^H-NMR (300 MHz, DMSO-*d*_6_): δ = 3.71 (3H, s, methyl), 3.56 (2H, s, 1,4-thiazine-3(4*H*)-one), 4.83 (2H, s, Sulfanylacetyl-H), 6.89–6.92 (2H, m, phenyl), 7.43–7.55 (2H, m, phenyl), 7.66 (1H, d, *J*_1_ = 1.79, benzothiazine-H), 7.69 (1H, d, *J*_1_ = 1.79, benzothiazine-H), 7.94 (1H, s, benzothiazine-H), 10.17 (1H, s, phenylamine-H), 10.77 (1H, s, thiazin-NH-); ^13^C-NMR (75 MHz, DMSO-*d*_6_): δ = 28.86, 41.67, 55.70, 114.76, 116.52, 119.70, 123.59, 126.94, 127.92, 134.06, 134.29, 138.11, 151.29, 155.07, 165.29, 166.05, 192.62. HRMS (*m*/*z*): [M+H]^+^ calcd for C_19_ H_16_ N_4_ O_3_ S_3:_ 445.449; found: 445.457.

*6-(2-((5-((4-chlorophenyl)amino)-1,3,4-thiadiazol-2-yl)sulfanyl)acetyl)-2H benzo[b][1,4]thiazin-3(4H)-one* (**3j**)

Yield: 95%, M.P.: 241–244 °C. IR (cm^−1^ bands): 3234 (N-H), 3194 (C-H), 1660 (C=O), 1244 (C-O); ^1^H-NMR (300 MHz, DMSO-*d*_6_): δ = 3.56 (2H, s, 1,4-thiazine-3(4*H*)-one), 4.88 (2H, s, Sulfanylacetyl-H), 7.35–7.38 (2H, m, phenyl), 7.50–7.59 (2H, m, phenyl), 7.67–7.70 (2H, dd, *J*_1_ = 1.8, *J*_2_ = 8.13 benzothiazine), 7.94 (1H, s, benzothiazine-H), 10.50 (1H, s, phenylamine-H), 10.78 (1H, s, thiazin-NH-); ^13^C-NMR (75 MHz, DMSO-*d*_6_): δ = 28.86, 41.57, 116.52, 119.34, 123.59, 125.82, 126.97, 127.94, 129.39, 134.08, 138,12, 139.67, 153.02, 164.92, 165.29, 192.53. HRMS (*m*/*z*): [M + H]^+^ calcd for C_19_ H_16_ N_4_ O_2_ S_3_ Cl:448.9962; found: 448.9976.

### 3.2. Cholinesterase Enzymes Inhibition Assay

The in vitro AChE and BChE inhibition potencies of the synthesized compounds (**3a**–**3j**) were evaluated according to the modified Ellman’s spectrophotometric method [33]. The reagents and materials used in the enzyme inhibition assay were supplied commercially by Sigma-Aldrich (St. Louis, MO, USA) and Fluka (Steinheim, Germany). The cholinesterase enzyme inhibition procedure was applied as reported in our previous research papers [32,34,35,36,37,38,39,40].

### 3.3. DPPH Free Radical Scavenging Antioxidant Activity

This is a method based on measuring the scavenging effects of the DPPH (1,1-Diphenyl-2- picrylhydrazyl) radical, a stable organic nitrogen-centered free radical. To prepare the DPHH solution, 9.86 mg of DPPH was measured and topped up to 25 mL with methanol. A total of 100 µL of DPPH solution and 100 µL of the test solutions were placed in the test wells. Only 200 µL of methanol was used for blank reading; 100 µL of methanol and 100 µL of DPPH solution were used for control. After incubation, spectrophotometric reading was performed at 517 nm [41,42].

### 3.4. In Vitro BBB Permeability Assay

To observe the BBB permeability of the active compounds **3i** and **3j**, the parallel artificial membrane permeability assay (PAMPA) was performed as previously described [21]. Briefly, the solutions of each compound were prepared in dimethyl sulfoxide (DMSO) at 10 mM and then diluted with PBS (Phosphate-Buffered Saline; pH = 7.4) to obtain the donor drug solution with the nominal final concentration of 100 μM (297 μL of buffer + 3 μL of DMSO drug solution). An amount of 100 μM is suitable for direct HPLC-DAD measurements. This solution was shaken for 1 h at room temperature in a 96-well polypropylene plate (Agilent, Waldbronn, Germany) and then filtered to avoid solid particles. The data were measured in 3 replicates on each plate. After the preparation of the solutions, the assay procedure was performed according to the kit method [43,44,45].

### 3.5. Cytotoxicity Assay

The NIH/3T3 mouse embryonic fibroblast cell line (ATCC^®^ CRL-1658™, London, UK) was used for cytotoxicity assays. The incubation period of NIH/3T3 cells was based on the supplier’s recommendation. NIH/3T3 cells were seeded at 1 × 10^4^ cells into each well of the 96-well plates. The MTT assay was carried out in accordance with the standards previously described [32,33,34].

### 3.6. Molecular Docking

A structure-based in silico procedure was applied to discover the binding modes of compounds **3i** and **3j** to the *h*AChE enzyme active site. The crystal structure of *h*AChE (PDB ID: 4EY7), which was crystallized with donepezil, was retrieved from the Protein Data Bank server (www.pdb.org) (accessed on 1 January 2022).

The structures of the ligands were built using the Schrödinger Maestro [46] interface and then were submitted to the Protein Preparation Wizard protocol of the Schrödinger Suite 2020 Update 2 [47]. The ligands were prepared using LigPrep 3.8 [48] to assign the protonation states at pH 7.4 ± 1.0 and the atom types correctly. Bond orders were assigned, and hydrogen atoms were added to the structures. The grid was generated using Glide 7.1 [49]. Flexible docking runs were performed with single precision docking mode (SP).

## 4. Conclusions

The designed series of 6-(2-((5-(substituted-amino)-1,3,4-thiadiazol-2-yl) sulfanyl) acetyl)-2*H*-benzo[b][1,4]thiazin-3(4*H*)-one compounds were successfully synthesized and characterized for their biological activity as acetylcholine esterase inhibitor agents. All compounds were analyzed using ^1^HNMR, ^13^CNMR, and HRMS spectrometric techniques. The compounds **3a**–**3j** showed no inhibitory activity toward BChE, while their enzyme inhibition activity was observed in vitro. As a result of the enzyme inhibition test, it was observed that all compounds were more effective against AChE. During the examination the biological activity of the compounds **3a**–**3j**, it was observed that derivatives with an electron-withdrawing substituent at position 4 of the phenyl ring showed greater inhibitory activity than non-substituted phenyl and aliphatic derivatives. Therefore, it can be said that the substituent in the fourth position is necessary for this activity. One of the derivatives containing substituents in this position carries methoxy **3i** and the other chlorine substituents **3j**. It was observed that the chlorine-bearing substituent **3i** was more active, with the IC_50_ value of 0.027 M, and **3j** that bears a methoxy group at position 4 showed an IC_50_ value of 0.025 M. Molecular docking studies were carried out to elucidate the reason for this. According to the docking study of the designed compounds **3a**–**3j**, it was demonstrated that the aromatic ring is required for the thiadiazole ring to exert its activity. This seems to be due to the interaction between aromatic substituents and Trp86 in the CAS region of the enzyme. In addition, if the aromatics moiety carries electronegative substitution at the para position, it also establishes aromatic hydrogen bonds with Glu202 and His447 and enhances its inhibitory activity. These bonds are also made by donepezil, and their properties show selectivity against AChE.

The antioxidant activities of compounds **3i** and **3j** showed great results in comparison to citric acid and ascorbic acid as antioxidative agents. As a result of these findings, it was revealed that compounds **3i** and **3j** may have potential effects on patients suffering from AD. In conclusion, based on this study, further molecular modification can be carried out on thiadiazole ring derivatives, and new molecules with higher affinity to AChE can be designed and may be subjected to future studies.

## Figures and Tables

**Table 1:** Synthesized compounds (**3a**–**3j**).

Compounds	R
**3a**	-CH_2_-CH_3_
**3b**	-CH_2_-CH_2_-CH_3_
**3c**	-CH(CH_3_)_2_
**3d**	-CH_2_(CH_2_)_2_-CH_3_
**3e**	-CH_2_-CH(CH_3_)_2_
**3f**	-Cyclohexyl
**3g**	-Phenyl
**3h**	-*p*-Tolyl
**3i**	-4-Methoxyphenyl
**3j**	-4-Chlorophenyl

**Table 2:** % Inhibition of the synthesized compounds, donepezil and tacrine against AChE and BChE.

Compound	AChE % Inhibition		BChE % Inhibition	
	10^−3^ M	10^−4^ M	10^−3^ M	10^−4^ M
**3a**	78.2 ± 1.2	48.8 ± 0.8	31.8 ± 0.8	25.1 ± 0.8
**3b**	75.4 ± 1.4	45.6 ± 0.9	34.9 ± 0.8	26.9 ± 0.6
**3c**	70.6 ± 1.0	40.1 ± 0.9	29.1 ± 0.8	20.4 ± 0.7
**3d**	72.7 ± 1.5	47.3 ± 0.9	36.1 ± 0.9	21.4 ± 0.9
**3e**	69.9 ± 1.2	46.2 ± 0.8	37.3 ± 0.8	24.6 ± 0.9
**3f**	65.4 ± 1.3	45.0 ± 1.0	30.5 ± 0.8	26.2 ± 0.8
**3g**	80.1 ± 1.2	44.5 ± 0.8	39.6 ± 0.9	24.8 ± 0.8
**3h**	82.2 ± 1.8	42.7 ± 0.8	35.6 ± 0.7	23.5 ± 0.7
**3i**	93.2 ± 1.4	90.5 ± 1.1	40.2 ± 0.9	28.0 ± 0.8
**3j**	96.5 ± 1.2	92.3 ± 1.3	42.5 ± 0.9	27.9 ± 0.7
**Donepezil**	99.3 ± 2.1	97.4 ± 1.9	-	-
**Tacrine**	-	-	98.3 ± 1.9	95.5 ± 1.3

**Table 3:** Type of blood–brain barrier (BBB) penetration of compounds **3i** and **3j**.

Classification	Type of BBB Permeation	Compounds	Type of BBB Permeation
CNS+	High BBB permeation *P_e_* (10^−6^ cm s^−1^) > 4.0	**3i**	CNS+ High BBB permeation
		**3j**	CNS+ High BBB permeation
CNS−	Low BBB permeation *P_e_* (10^−6^ cm s^−1^) < 2.0		
CNS±	BBB permeation uncertain 2.0 < *P_e_* (10^−6^ cm s^−1^) < 4.0		

## Data Availability

Not available.

## Supplementary Materials

The following supporting information can be downloaded at: https://www.mdpi.com/article/10.3390/molecules27072121/s1, Figures S1–S10. HRMS of **3a**–**3j**; Figures S11–S20. ^1^HNMR of **3a**–**3j**; Figures S21–S29. ^13^CNMR of **3a**–**3c**, **3e**–**3j**; Figures S30–S39. IR spectrum of **3a**–**3j**; Figures S40–S49. ^1^HNMR of **2a**–**2j**.

## Sample Availability

Samples of the compounds **2a**–**2j** and **3a**–**3j** are available from the authors.
