# Supplementary material for: Design, Synthesis, and Evaluation of Novel 2H-Benzo[b][1,4]thiazin-3(4H)-one Derivatives as New Acetylcholinesterase Inhibitors
Source: Molecules. 2022 Mar 25;27(7):2121. doi: 10.3390/molecules27072121 (PMC9000418; doi:10.3390/molecules27072121)

molecules

MDPI

Design, Synthesis and In Vitro Evaluation of Acetylcholinesterase Inhibitory Activity Of Novel Thiadiazol Derivatives

Sazan Hajiali[1], Derya Osmaniye[1,2], Begüm Nurpelin Sağlık[1,2], Yusuf Özkay[1,2], Zafer Asım Kaplancıklı[1*]

1 Department of Pharmaceutical Chemistry, Faculty of Pharmacy, Anadolu University, 26470 Eskişehir, Turkey
2 Doping and Narcotic Compounds Analysis Laboratory, Faculty of Pharmacy, Anadolu University, 26470 Eskişehir, Turkey

*Correspondence:
Prof. Dr. Zafer Asım KAPLANCIKLI, Department of Pharmaceutical Chemistry, Faculty of Pharmacy, Anadolu University, 26470 Eskişehir, Turkey
Email: zakaplan@anadolu.edu.tr

Citation: Haji Ali, S.; Osmaniye, D.; Sağlık, B.N.; Levent, S.; Özkay, Y.; Kaplancıklı, Z.A. Design, Synthesis, and Evaluation of Novel 2*H*-Benzo[b][1,4]thiazin-3(4*H*)-one Derivatives as New Acetylcholinesterase Inhibitors. *Molecules* **2022**, *27*, 2121. https://doi.org/10.3390/molecules27072121

Academic Editor: Athina Geronikaki

Received: 28 February 2022
Accepted: 22 March 2022
Published: 25 March 2022

Publisher's Note: MDPI stays neutral with regard to jurisdictional claims in published maps and institutional affiliations.

Copyright: © 2022 by the authors. Licensee MDPI, Basel, Switzerland. This article is an open access article distributed under the terms and conditions of the Creative Commons Attribution (CC BY) license (https://creativecommons.org/licenses/by/4.0/).

Supplementary Data:

Figure S1. HRMS of 3a:

Data File: C:\LabSolutions\Data\Analiz\derya\COMP-1_99.lcd

Elmt	Val.	Min	Max	Elmt	Val.	Min	Max	Elmt	Val.	Min	Max	Elmt	Val.	Min	Max	Use Adduct
H	1	10	40	O	2	1	7	S	2	2	3	Ru	2	0	0	H
C	4	10	40	F	1	0	0	Cl	1	0	0	Pd	2	0	0	
N	3	2	7	P	3	0	0	Br	1	0	1	I	3	0	0	

Error Margin (ppm): 10
HC Ratio: unlimited
Max Isotopes: 3
MSn Iso RI (%): 10.00

DBE Range: 10.0 - 25.0
Apply N Rule: yes
Isotope RI (%): 1.00
MSn Logic Mode: AND

Electron Ions: both
Use MSn Info: yes
Isotope Res: 9000
Max Results: 150

Event#: 1 MS(E+) Ret. Time : 3.467 Scan# : 521

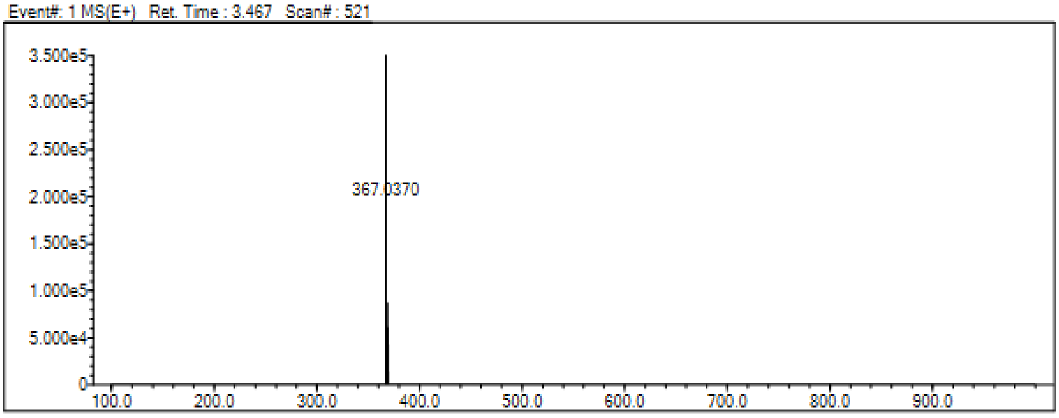

Measured region for 367.0370 m/z

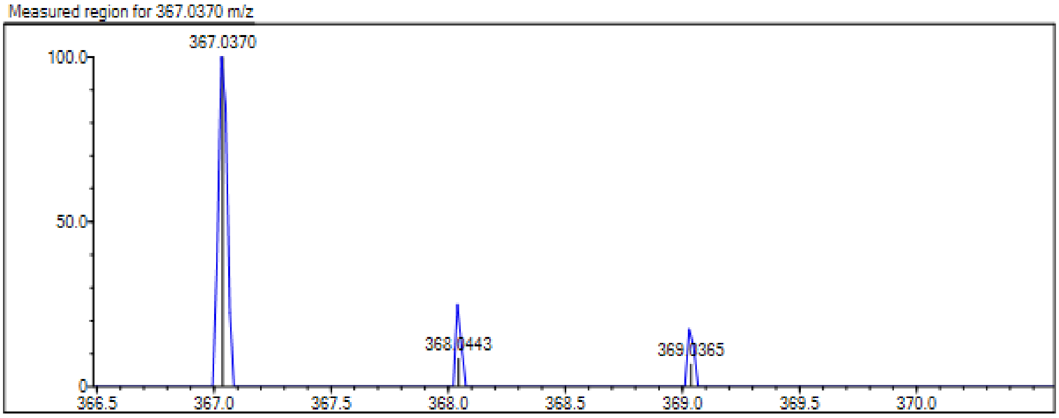

C14 H14 N4 O2 S3 [M+H]+ : Predicted region for 367.0352 m/z

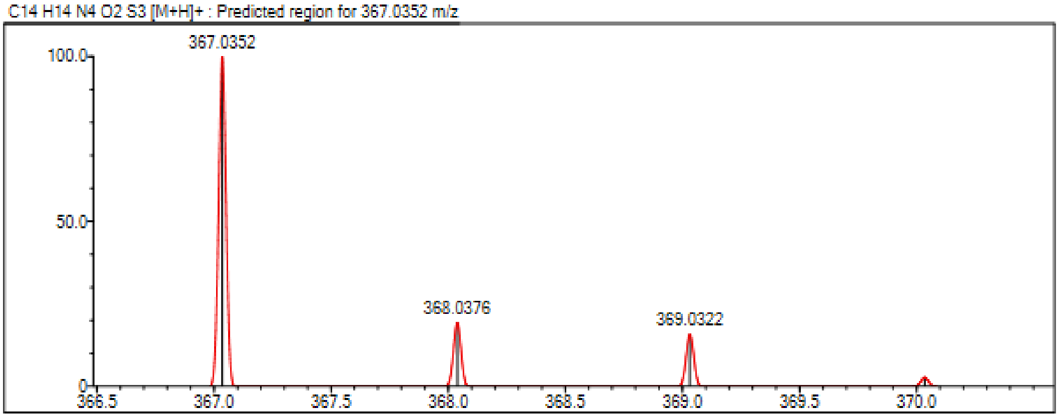

Rank	Score	Formula (M)	Ion	Meas. m/z	Pred. m/z	Df. (mDa)	Df. (ppm)	Iso	DBE
1	58.68	C14 H14 N4 O2 S3	[M+H]+	367.0370	367.0352	1.8	4.90	65.02	10.0

Figure S2. HRMS of 3b:

Data File: C:\LabSolutions\Data\Analiz\derya\COMP-3_103.lcd

Elmt	Val.	Min	Max	Elmt	Val.	Min	Max	Elmt	Val.	Min	Max	Elmt	Val.	Min	Max	Use Adduct
H	1	10	40	O	2	2	5	S	2	1	3	Ru	2	0	0	H
C	4	10	40	F	1	0	0	Cl	1	0	0	Pd	2	0	0	
N	3	2	7	P	3	0	0	Br	1	0	0	I	3	0	0	

Error Margin (ppm): 5
HC Ratio: unlimited
Max Isotopes: 3
MSn Iso RI (%): 10.00

DBE Range: 5.0 - 25.0
Apply N Rule: yes
Isotope RI (%): 1.00
MSn Logic Mode: AND

Electron Ions: both
Use MSn Info: yes
Isotope Res: 9000
Max Results: 150

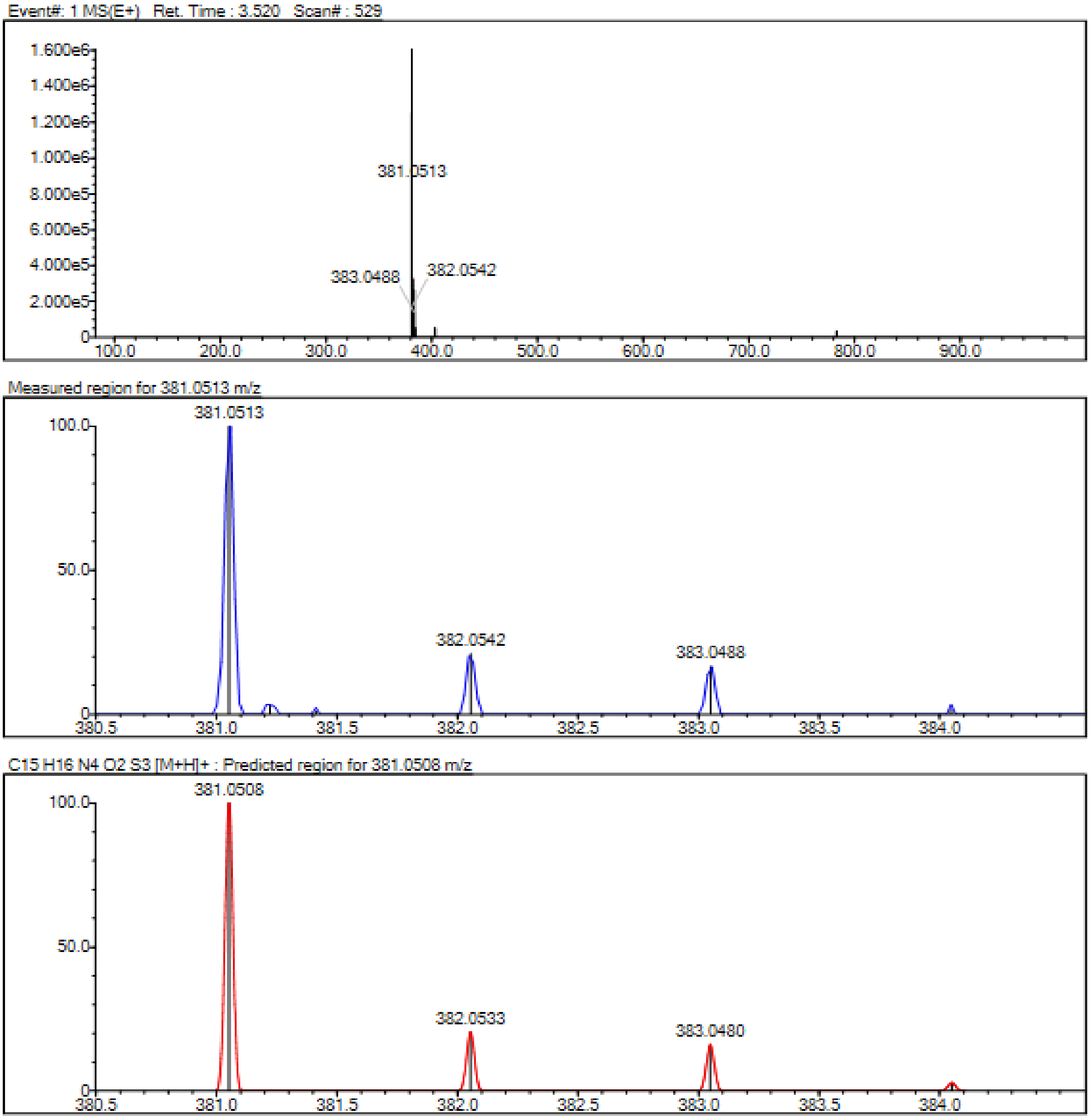

Rank	Score	Formula (M)	Ion	Meas. m/z	Pred. m/z	Df. (mDa)	Df. (ppm)	Iso	DBE
1	88.22	C15 H16 N4 O2 S3	[M+H]+	381.0513	381.0508	0.5	1.31	88.91	10.0

Figure S3. HRMS of 3c:

Data File: C:\LabSolutions\Data\Analiz\derya\COMP-4_102.lcd

Elmt	Val.	Min	Max	Elmt	Val.	Min	Max	Elmt	Val.	Min	Max	Elmt	Val.	Min	Max	Use Adduct
H	1	10	40	O	2	1	7	S	2	2	3	Ru	2	0	0	H
C	4	10	40	F	1	0	0	Cl	1	0	0	Pd	2	0	0	
N	3	2	7	P	3	0	0	Br	1	0	1	I	3	0	0	

Error Margin (ppm): 10
HC Ratio: unlimited
Max Isotopes: 3
MSn Iso RI (%): 10.00

DBE Range: 10.0 - 25.0
Apply N Rule: yes
Isotope RI (%): 1.00
MSn Logic Mode: AND

Electron Ions: both
Use MSn Info: yes
Isotope Res: 9000
Max Results: 150

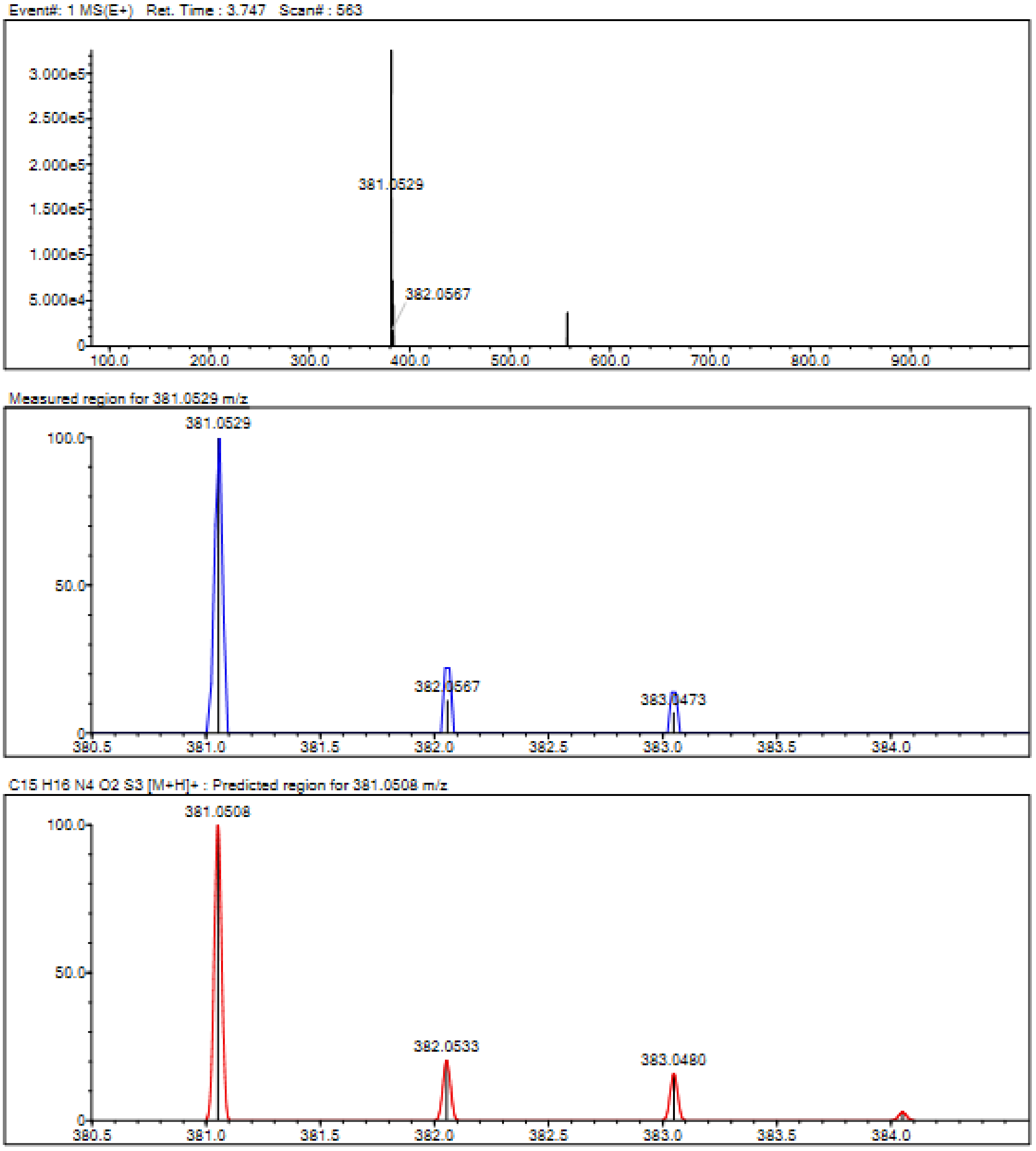

Rank	Score	Formula (M)	Ion	Meas. m/z	Pred. m/z	Df. (mDa)	Df. (ppm)	Iso	DBE
1	52.99	C15 H16 N4 O2 S3	[M+H]+	381.0529	381.0508	2.1	5.51	62.42	10.0

Figure S4. HRMS of 3d:

Data File: C:\LabSolutions\Data\Analiz\derya\COMP-5_2_176.lcd

Elmt	Val.	Min	Max	Elmt	Val.	Min	Max	Elmt	Val.	Min	Max	Elmt	Val.	Min	Max	Use Adduct
H	1	10	40	O	2	0	3	S	2	1	3	Ru	2	0	0	H
C	4	10	40	F	1	0	0	Cl	1	0	0	Pd	2	0	0	
N	3	3	7	P	3	0	0	Br	1	0	0	I	3	0	0	

Error Margin (ppm):	5	DBE Range:	5.0 - 20.0	Electron Ions:	both
HC Ratio:	unlimited	Apply N Rule:	yes	Use MSn Info:	yes
Max Isotopes:	3	Isotope RI (%):	1.00	Isotope Res:	9000
MSn Iso RI (%):	10.00	MSn Logic Mode:	AND	Max Results:	150

Event#: 1 MS(E+) Ret. Time : 2.613 Scan# : 393

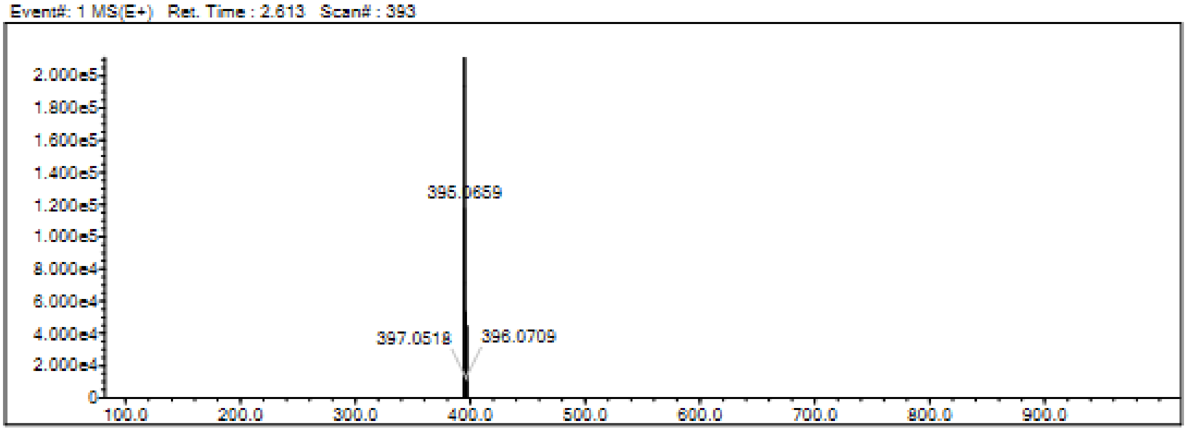

Measured region for 395.0659 m/z

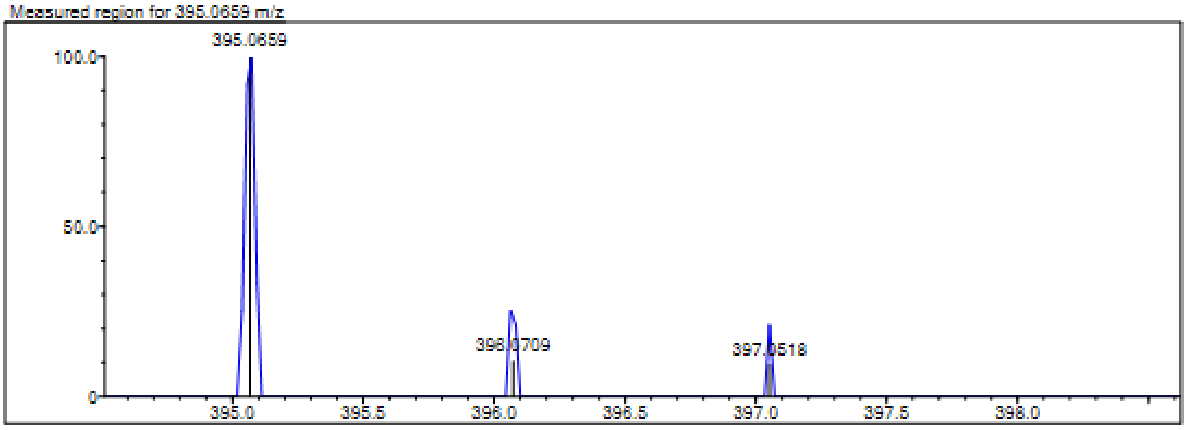

C16 H18 N4 O2 S3 [M+H]+ : Predicted region for 395.0665 m/z

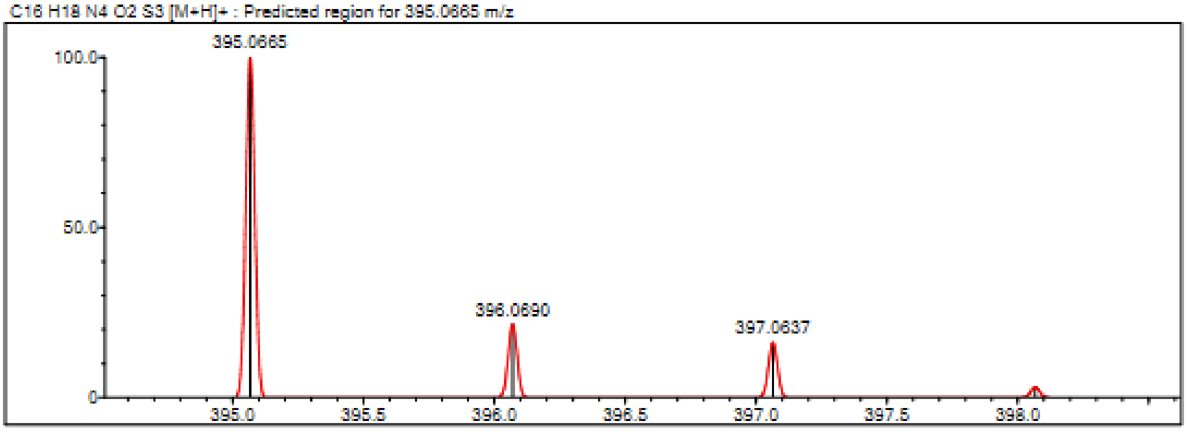

Rank	Score	Formula (M)	Ion	Meas. m/z	Pred. m/z	Df. (mDa)	Df. (ppm)	Iso	DBE
1	46.37	C16 H18 N4 O2 S3	[M+H]+	395.0659	395.0665	-0.6	-1.52	46.98	10.0

Figure S5. HRMS of 3e:

Data File: C:\LabSolutions\Data\Analiz\derya\COMP-6_106.lcd

Elmt	Val.	Min	Max	Elmt	Val.	Min	Max	Elmt	Val.	Min	Max	Elmt	Val.	Min	Max	Use Adduct
H	1	10	40	O	2	2	5	S	2	1	3	Ru	2	0	0	H
C	4	10	40	F	1	0	0	Cl	1	0	0	Pd	2	0	0	
N	3	2	7	P	3	0	0	Br	1	0	0	I	3	0	0	

Error Margin (ppm):	5	DBE Range:	5.0 - 25.0	Electron Ions:	both
HC Ratio:	unlimited	Apply N Rule:	yes	Use MSn Info:	yes
Max Isotopes:	3	Isotope RI (%):	1.00	Isotope Res:	9000
MSn Iso RI (%):	10.00	MSn Logic Mode:	AND	Max Results:	150

Event#: 1 MS(E+) Ret. Time : 3.747 Scan# : 563

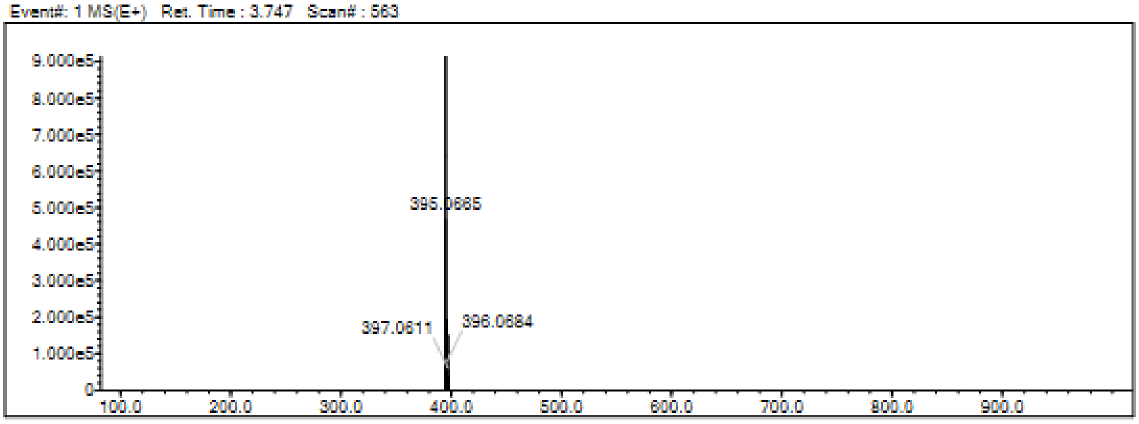

Measured region for 395.0665 m/z

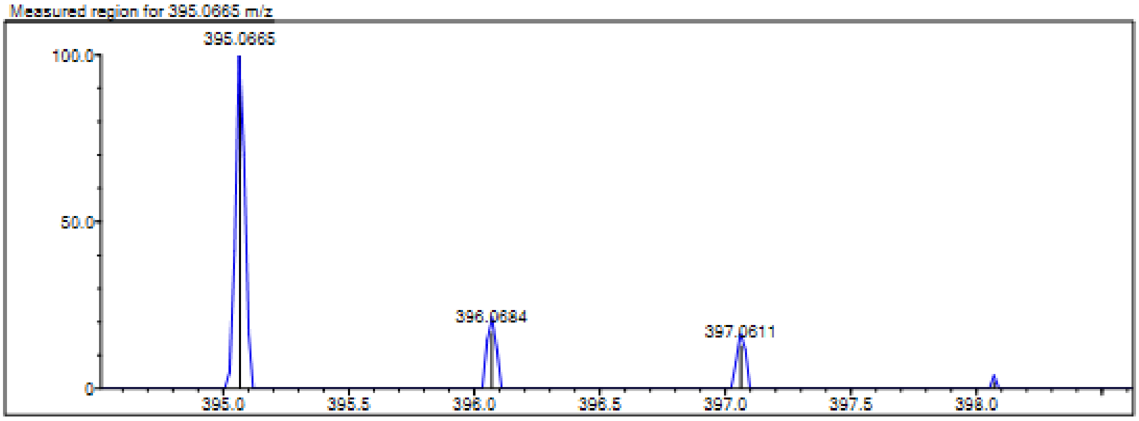

C16 H18 N4 O2 S3 [M+H]+ : Predicted region for 395.0665 m/z

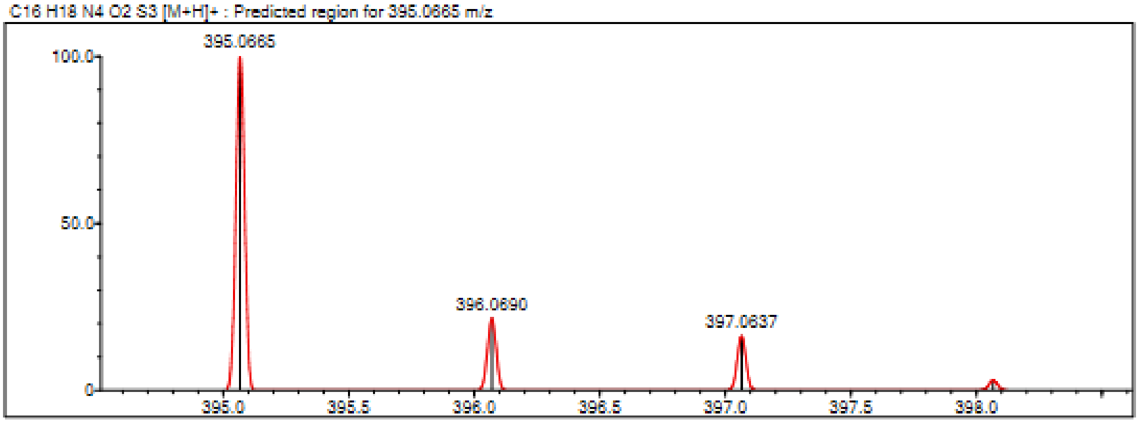

Rank	Score	Formula (M)	Ion	Meas. m/z	Pred. m/z	Df. (mDa)	Df. (ppm)	Iso	DBE
1	85.40	C16 H18 N4 O2 S3	[M+H]+	395.0665	395.0665	0.0	0.00	85.40	10.0

Figure S6. HRMS of 3f:

Elmt	Val.	Min	Max	Elmt	Val.	Min	Max	Elmt	Val.	Min	Max	Elmt	Val.	Min	Max	Use Adduct
H	1	10	40	O	2	2	5	S	2	1	3	Ru	2	0	0	H
C	4	10	40	F	1	0	0	Cl	1	0	0	Pd	2	0	0	
N	3	2	7	P	3	0	0	Br	1	0	0	I	3	0	0	

Error Margin (ppm): 5
HC Ratio: unlimited
Max Isotopes: 3
MSn Iso RI (%): 10.00

DBE Range: 5.0 - 25.0
Apply N Rule: yes
Isotope RI (%): 1.00
MSn Logic Mode: AND

Electron Ions: both
Use MSn Info: yes
Isotope Res: 9000
Max Results: 150

Event#: 1 MS(E+) Ret. Time : 4.160 Scan# : 625

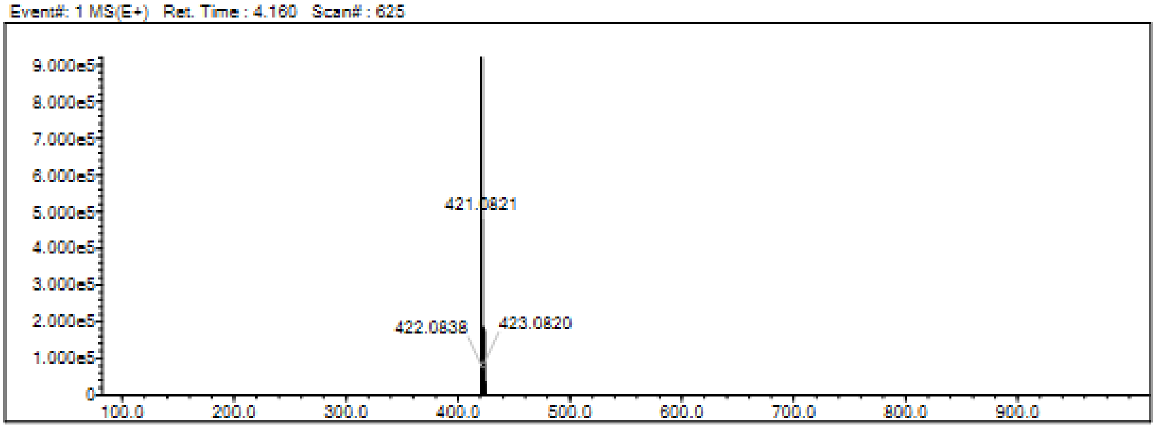

Measured region for 421.0821 m/z

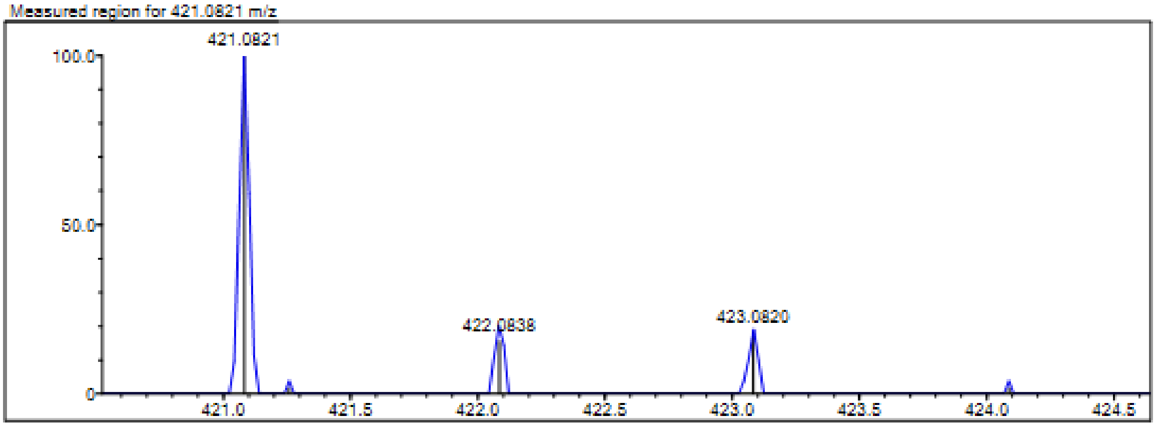

C18 H20 N4 O2 S3 [M+H]+ : Predicted region for 421.0821 m/z

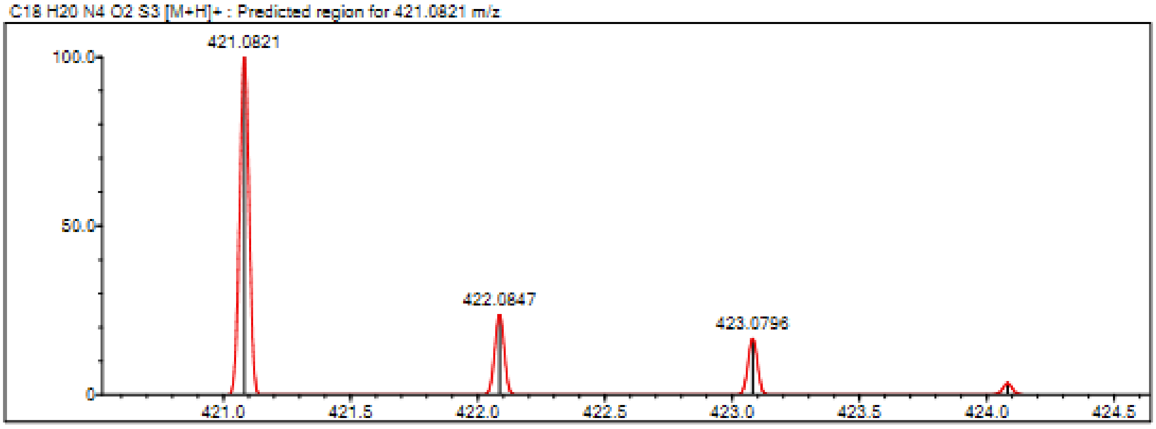

Rank	Score	Formula (M)	Ion	Meas. m/z	Pred. m/z	Df. (mDa)	Df. (ppm)	Iso	DBE
1	87.90	C18 H20 N4 O2 S3	[M+H]+	421.0821	421.0821	-0.0	0.00	87.90	11.0

Figure S7. HRMS of 3g:

Data File: C:\LabSolutions\Data\Analiz\derya\COMP-8_108.lcd

Elmt	Val.	Min	Max	Elmt	Val.	Min	Max	Elmt	Val.	Min	Max	Elmt	Val.	Min	Max	Use Adduct
H	1	10	40	O	2	2	5	S	2	1	3	Ru	2	0	0	H
C	4	10	40	F	1	0	0	Cl	1	0	0	Pd	2	0	0	
N	3	2	7	P	3	0	0	Br	1	0	0	I	3	0	0	

Error Margin (ppm):	5	DBE Range:	5.0 - 25.0	Electron Ions:	both
HC Ratio:	unlimited	Apply N Rule:	yes	Use MSn Info:	yes
Max Isotopes:	3	Isotope RI (%):	1.00	Isotope Res:	9000
MSn Iso RI (%):	10.00	MSn Logic Mode:	AND	Max Results:	150

Event#: 1 MS(E+) Ret. Time : 3.853 Scan# : 579

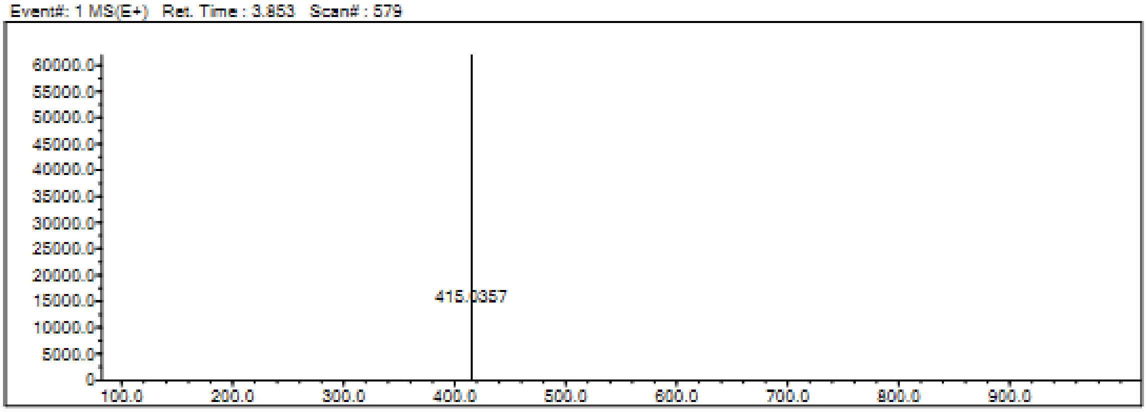

Measured region for 415.0357 m/z

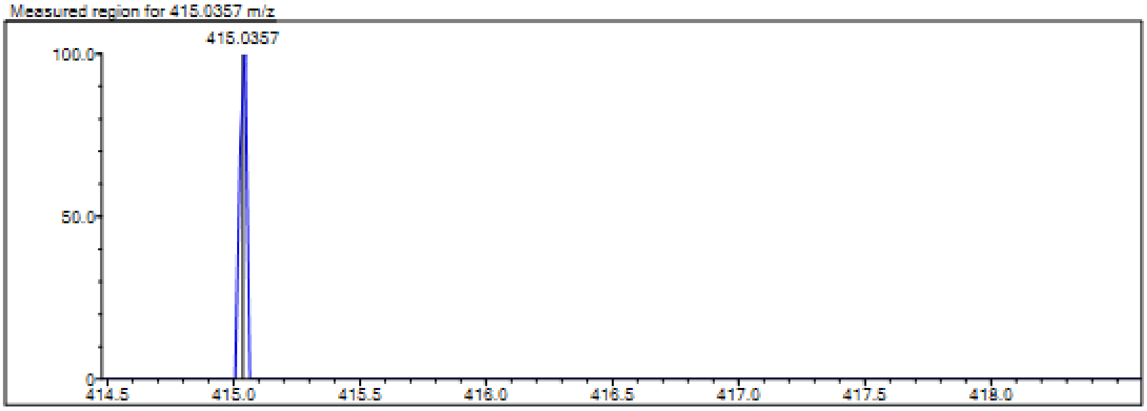

C18 H14 N4 O2 S3 [M+H]+ : Predicted region for 415.0352 m/z

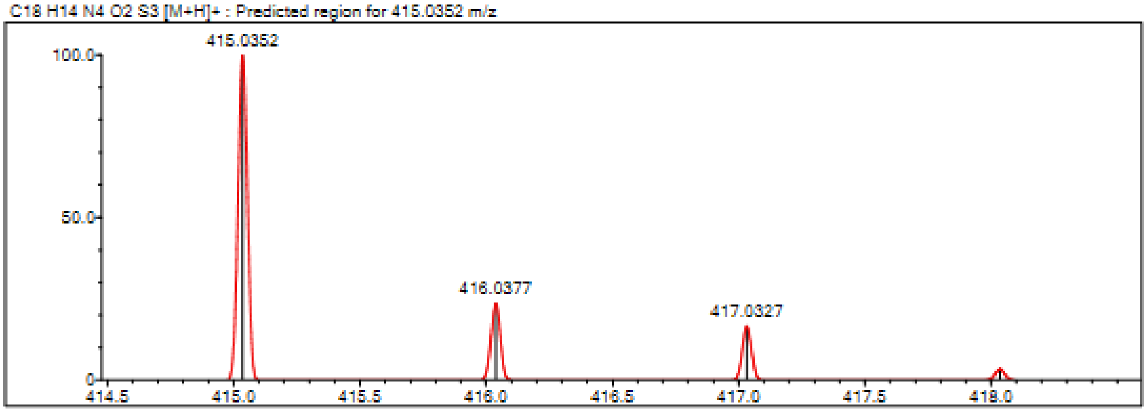

Rank	Score	Formula (M)	Ion	Meas. m/z	Pred. m/z	Df. (mDa)	Df. (ppm)	Iso	DBE
1	0.00	C18 H14 N4 O2 S3	[M+H]+	415.0357	415.0352	0.5	1.20	0.00	14.0

Figure S8. HRMS of 3h:

Data File: C:\LabSolutions\Data\Analiz\derya\COMP-9_109.lcd

Elmt	Val.	Min	Max	Elmt	Val.	Min	Max	Elmt	Val.	Min	Max	Elmt	Val.	Min	Max	Use Adduct
H	1	10	40	O	2	2	5	S	2	1	3	Ru	2	0	0	H
C	4	10	40	F	1	0	0	Cl	1	0	0	Pd	2	0	0	
N	3	2	7	P	3	0	0	Br	1	0	0	I	3	0	0	

Error Margin (ppm): 5
HC Ratio: unlimited
Max Isotopes: 3
MSn Iso RI (%): 10.00

DBE Range: 5.0 - 25.0
Apply N Rule: yes
Isotope RI (%): 1.00
MSn Logic Mode: AND

Electron Ions: both
Use MSn Info: yes
Isotope Res: 9000
Max Results: 150

Event#: 1 MS(E+) Ret. Time : 4.027 -> 4.560 Scan# : 605 -> 685

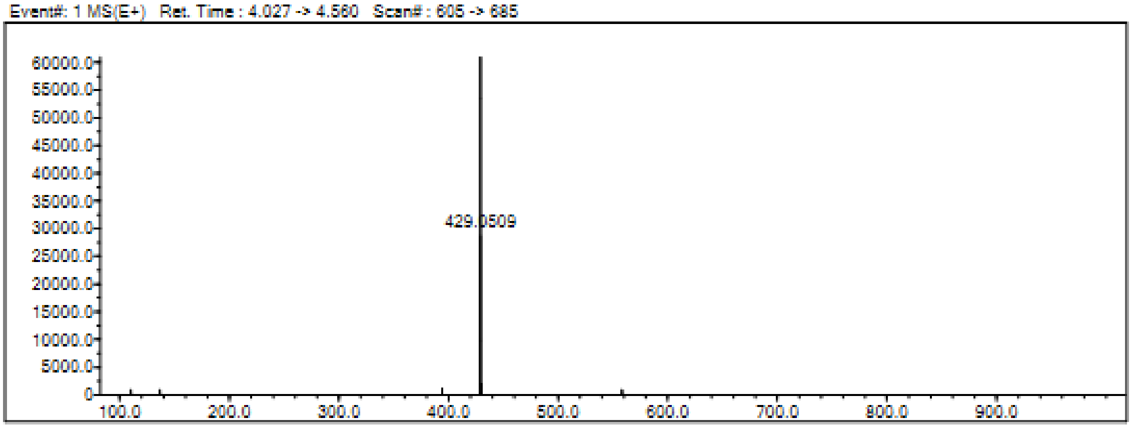

Measured region for 429.0509 m/z

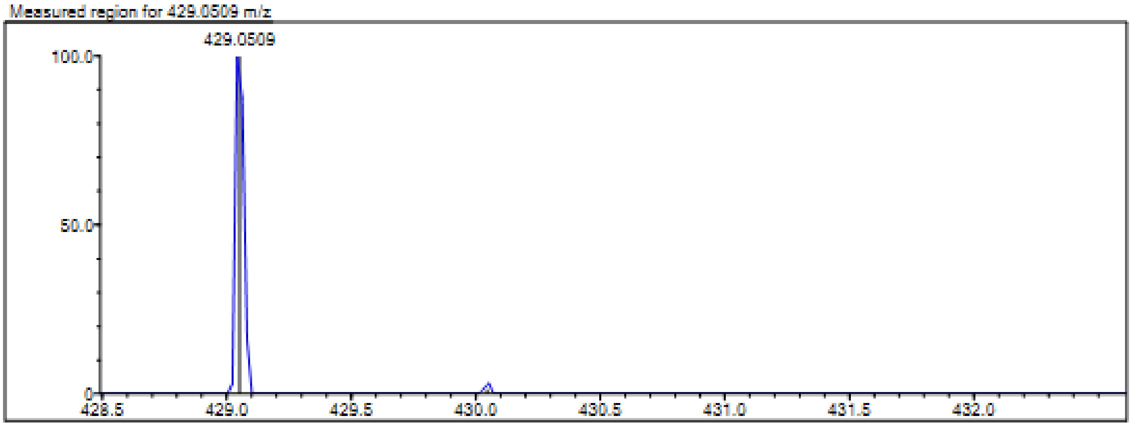

C19 H16 N4 O2 S3 [M+H]+ : Predicted region for 429.0508 m/z

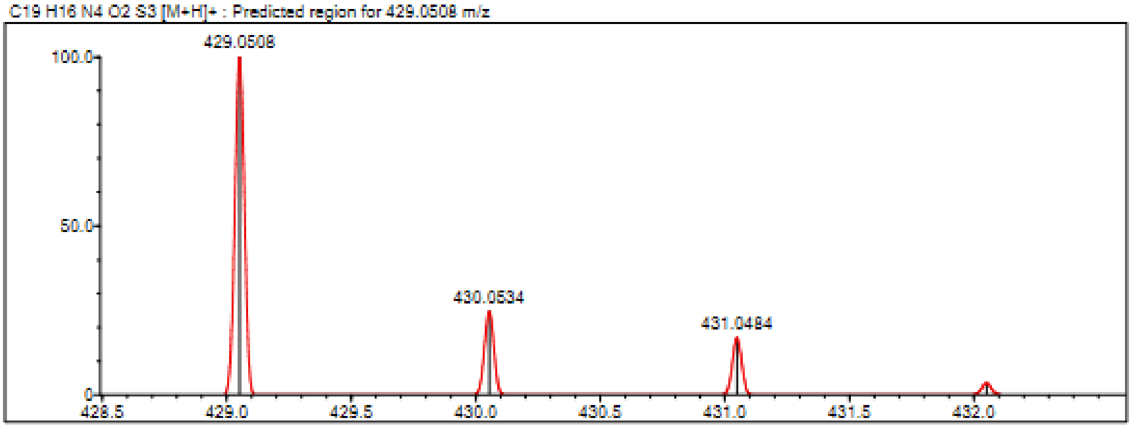

Rank	Score	Formula (M)	Ion	Meas. m/z	Pred. m/z	Df. (mDa)	Df. (ppm)	Iso	DBE
1	0.00	C19 H16 N4 O2 S3	[M+H]+	429.0509	429.0508	0.1	0.23	0.00	14.0

Figure S9. HRMS of 3i:

Data File: C:\LabSolutions\Data\Analiz\derya\COMP-10_111.lcd

Elmt	Val.	Min	Max	Elmt	Val.	Min	Max	Elmt	Val.	Min	Max	Elmt	Val.	Min	Max	Use Adduct
H	1	10	40	O	2	0	3	S	2	1	3	Ru	2	0	0	H
C	4	10	40	F	1	0	0	Cl	1	0	0	Pd	2	0	0	
N	3	3	7	P	3	0	0	Br	1	0	0	I	3	0	0	

Error Margin (ppm): 5
HC Ratio: unlimited
Max Isotopes: 3
MSn Iso RI (%): 10.00

DBE Range: 5.0 - 20.0
Apply N Rule: yes
Isotope RI (%): 1.00
MSn Logic Mode: AND

Electron Ions: both
Use MSn Info: yes
Isotope Res: 9000
Max Results: 150

Event#: 1 MS(E+) Ret. Time : 3.507 -> 3.840 Scan# : 527 -> 577

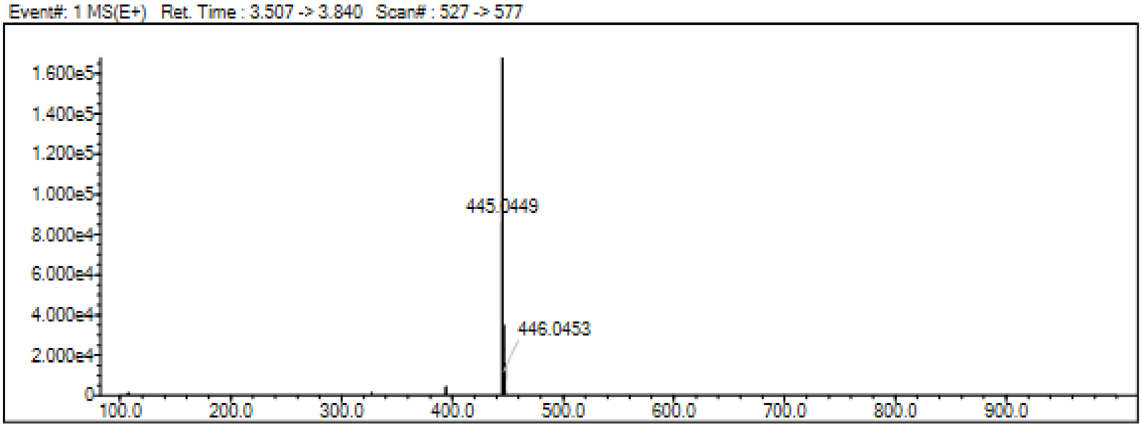

Measured region for 445.0449 m/z

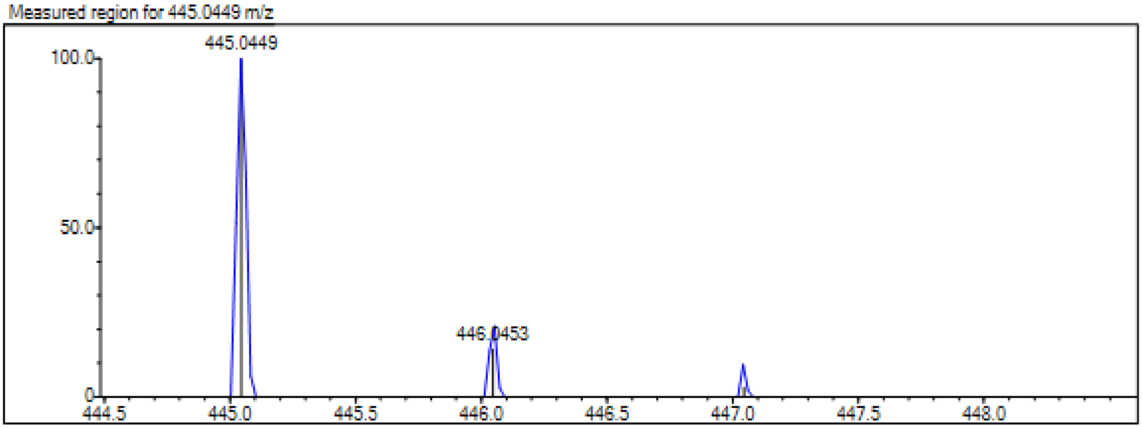

C19 H16 N4 O3 S3 [M+H]+ : Predicted region for 445.0457 m/z

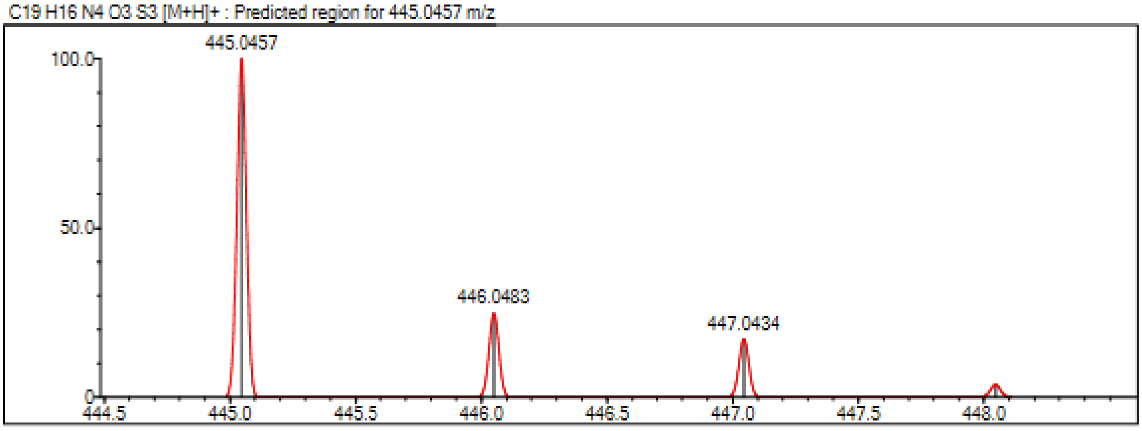

Rank	Score	Formula (M)	Ion	Meas. m/z	Pred. m/z	Df. (mDa)	Df. (ppm)	Iso	DBE
1	67.37	C19 H16 N4 O3 S3	[M+H]+	445.0449	445.0457	-0.8	-1.80	68.75	14.0

Figure S10. HRMS of 3j:

Data File: C:\LabSolutions\Data\Analiz\derya\COMP-11_1.lcd

Elmt	Val.	Min	Max	Elmt	Val.	Min	Max	Elmt	Val.	Min	Max	Elmt	Val.	Min	Max	Use Adduct
H	1	5	40	O	2	0	4	S	2	0	3	Ru	2	0	0	H
C	4	5	30	F	1	0	0	Cl	1	0	1	Pd	2	0	0	
N	3	0	6	P	3	0	0	Br	1	0	0	I	3	0	0	

Error Margin (ppm): 5
HC Ratio: unlimited
Max Isotopes: 3
MSn Iso RI (%): 10.00

DBE Range: 5.0 - 17.0
Apply N Rule: yes
Isotope RI (%): 1.00
MSn Logic Mode: AND

Electron Ions: both
Use MSn Info: yes
Isotope Res: 9000
Max Results: 200

Event#: 1 MS(E+) Ret. Time : 3.227 -> 3.333 Scan# : 485 -> 501

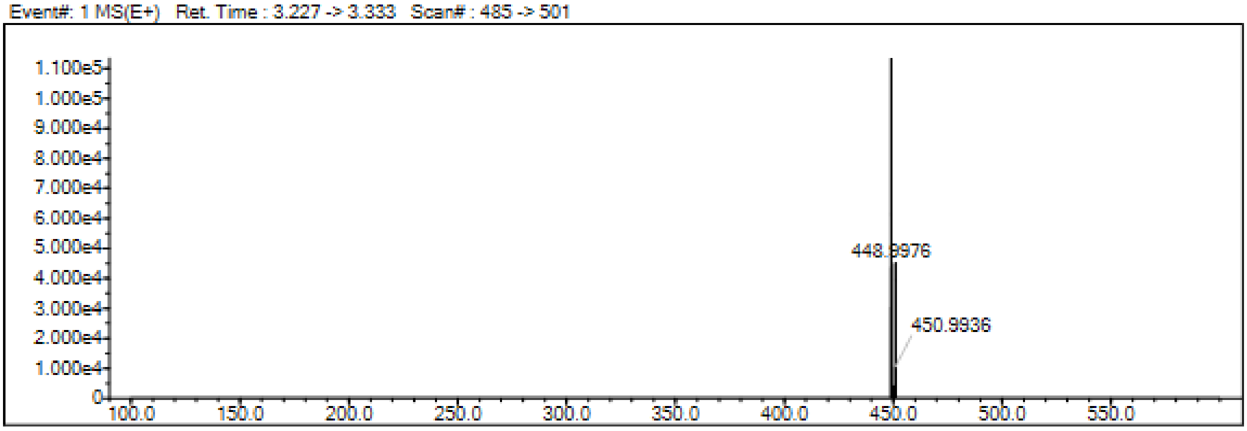

Measured region for 448.9976 m/z

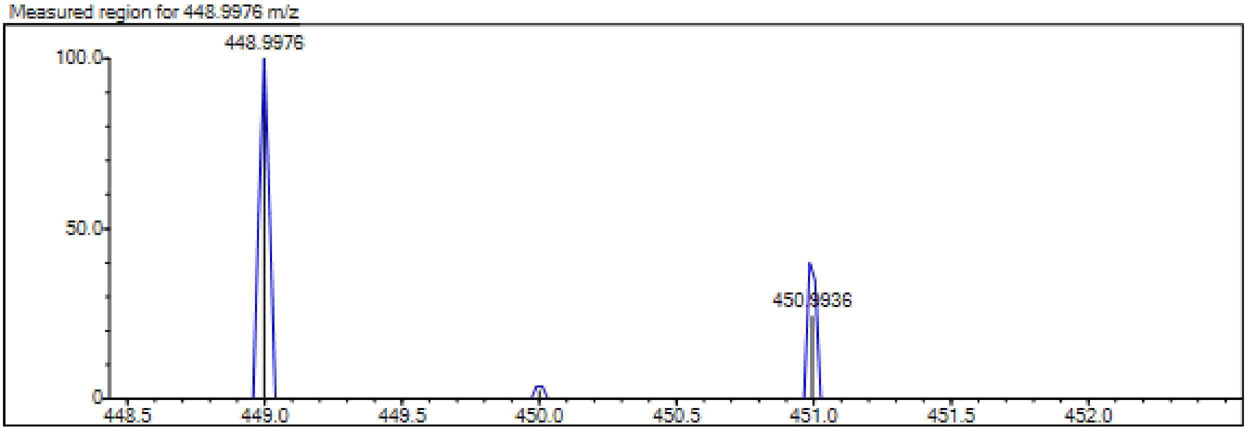

C18 H13 N4 O2 S3 Cl [M+H]+ : Predicted region for 448.9962 m/z

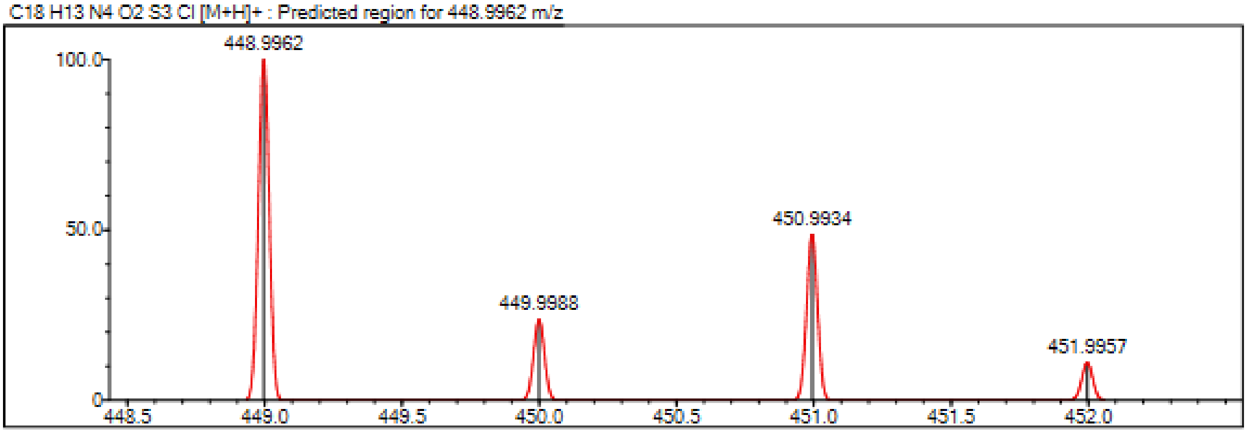

Rank	Score	Formula (M)	Ion	Meas. m/z	Pred. m/z	Df. (mDa)	Df. (ppm)	Iso	DBE
1	50.91	C18 H13 N4 O2 S3 Cl	[M+H]+	448.9976	448.9962	1.4	3.12	53.75	14.0

^{1}HNMR of Compound 3a-3j:

Figure S11. ^{1}HNMR 3a:

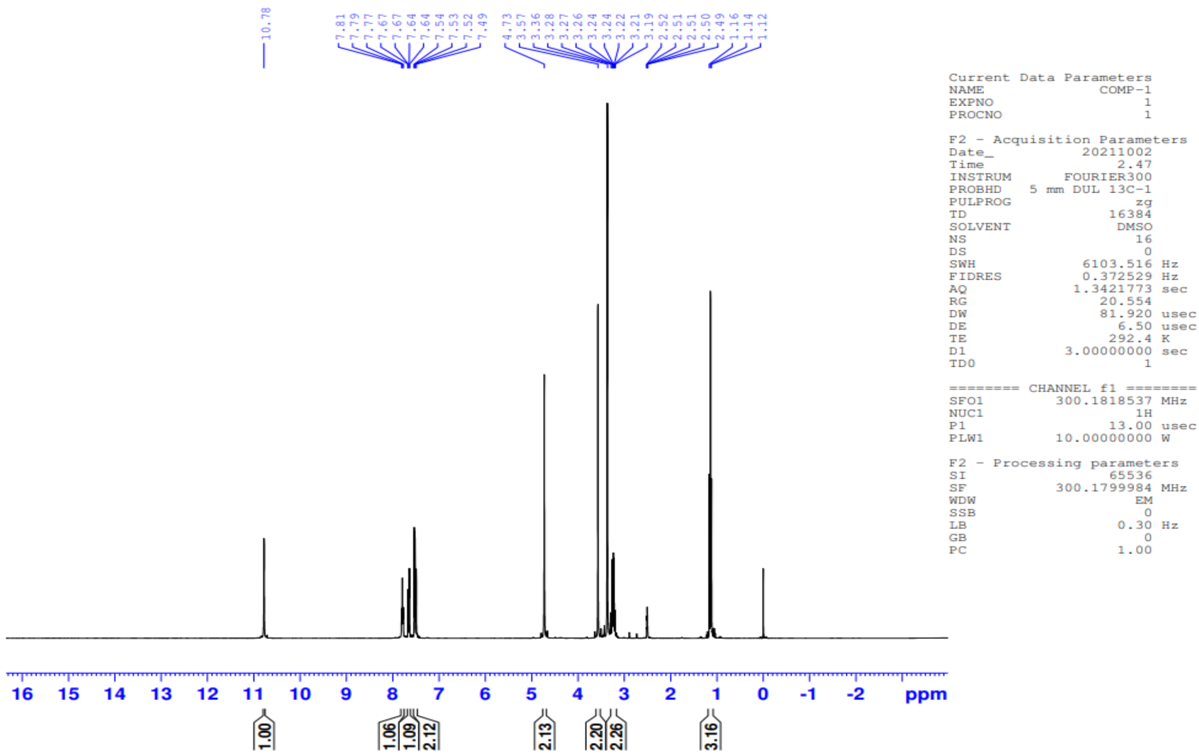

Figure S12. ^{1}HNMR 3b:

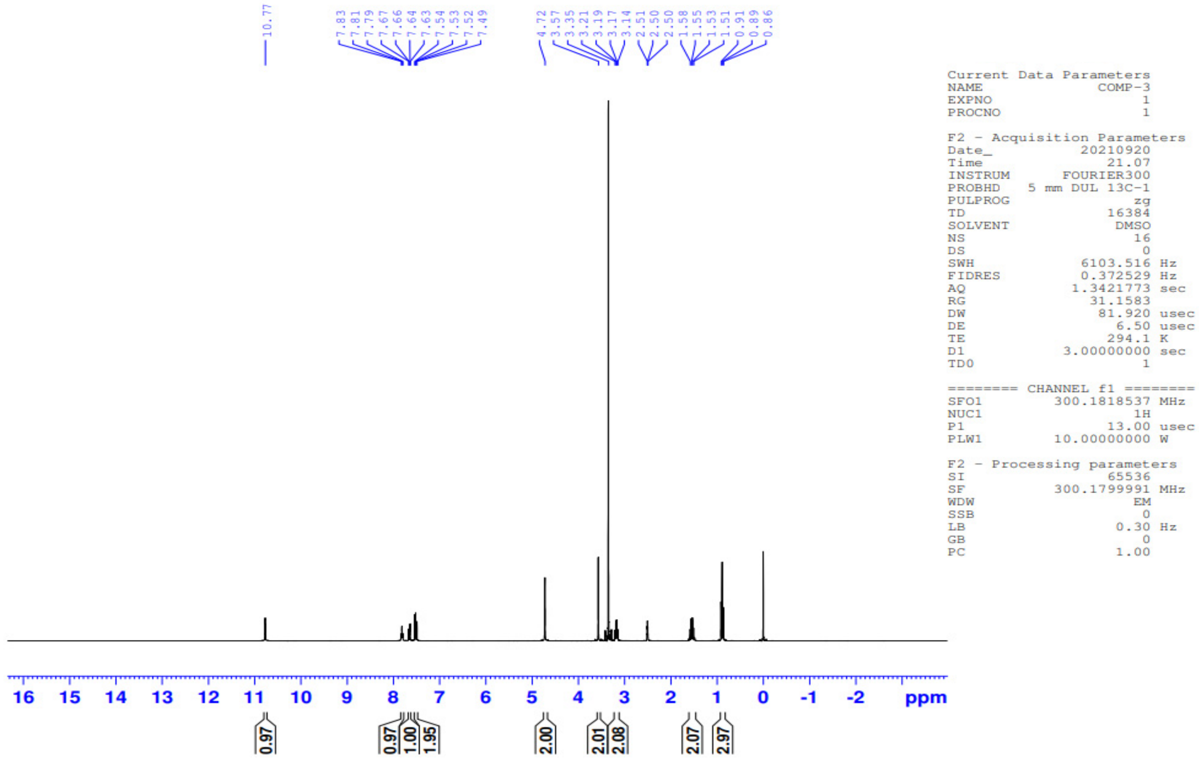

Figure S13. ^{1}HNMR 3c:

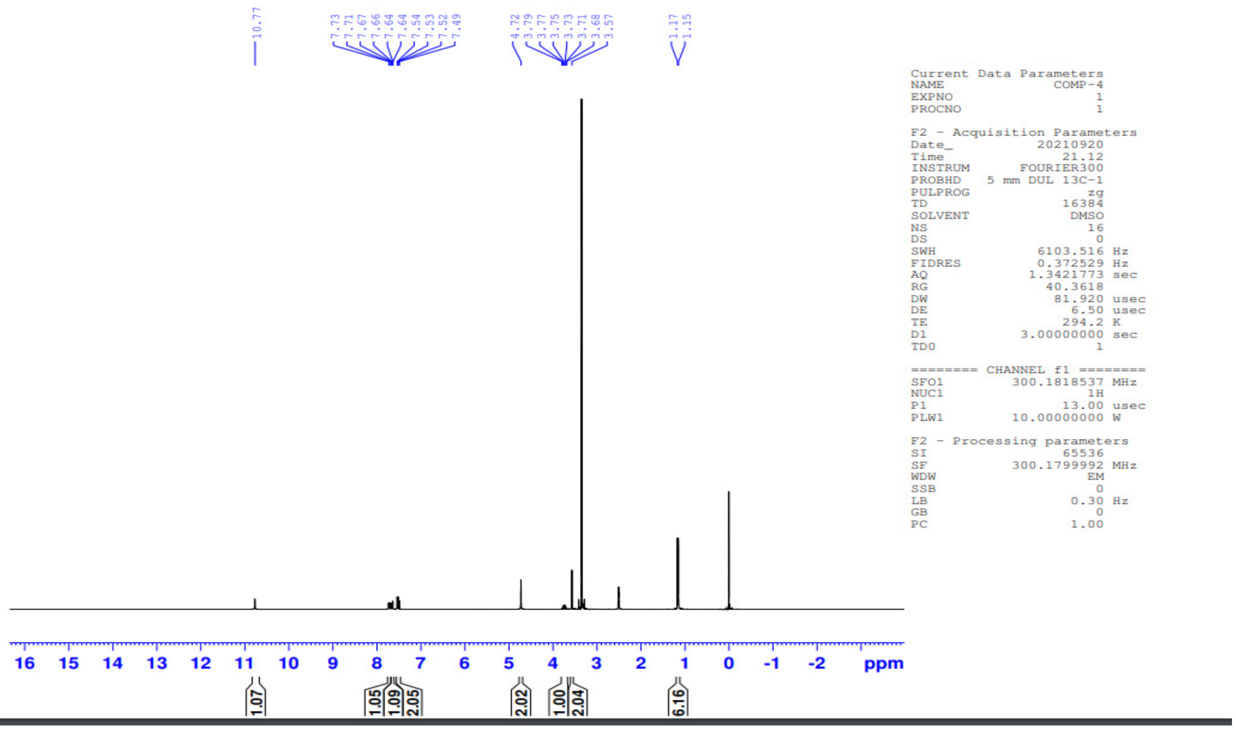

Figure S14. ^{1}HNMR 3d:

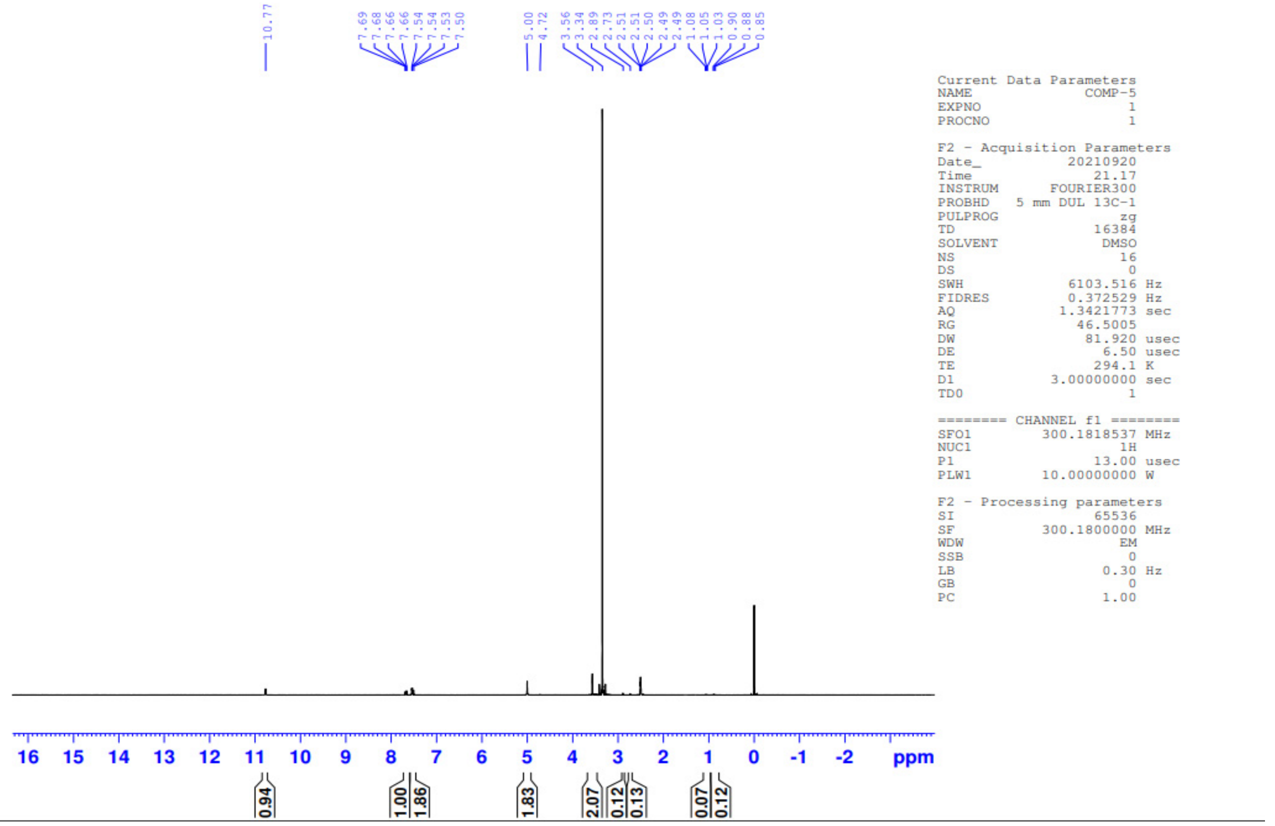

Figure S15. ^{1}**HNMR 3e:**

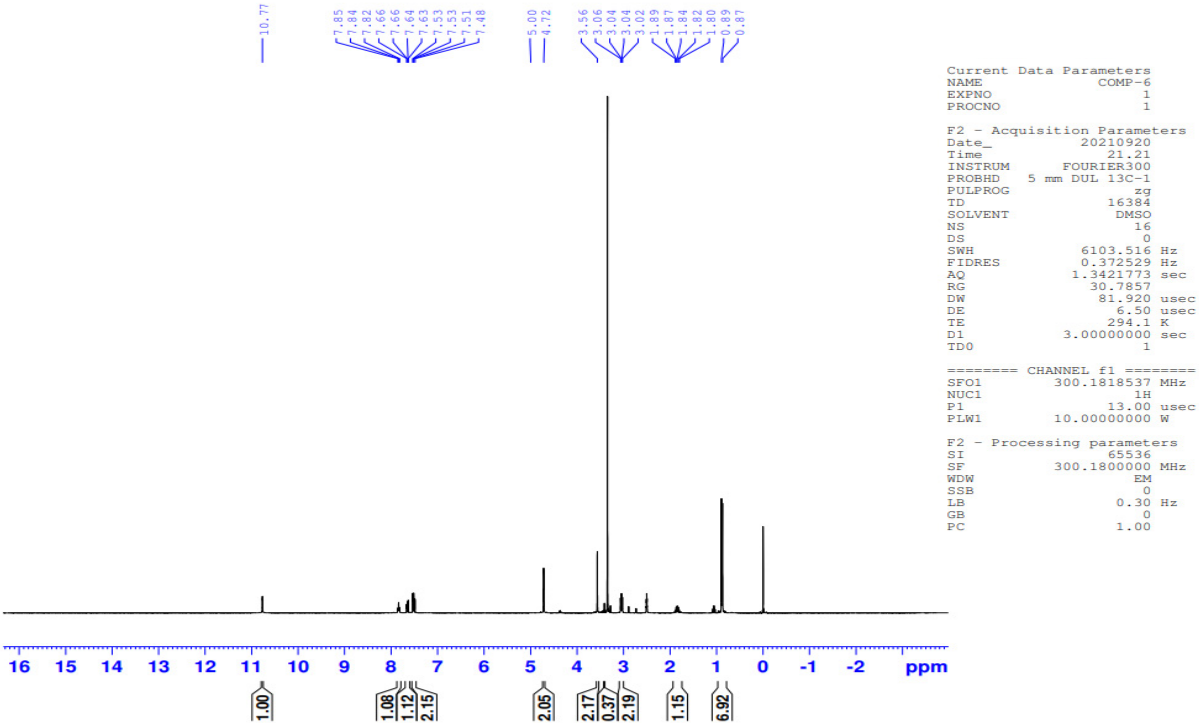

Figure S16. ^{1}**HNMR 3f:**

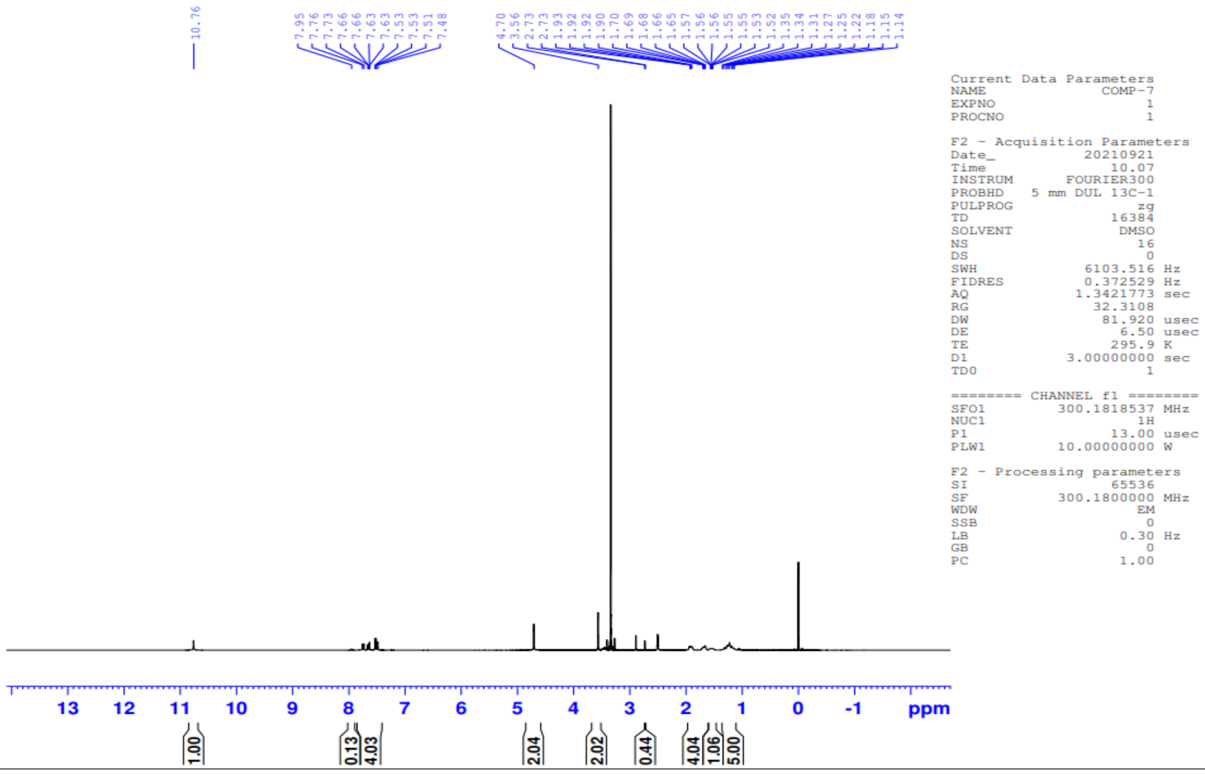

Figure S17. ^{1}HNMR 3g:

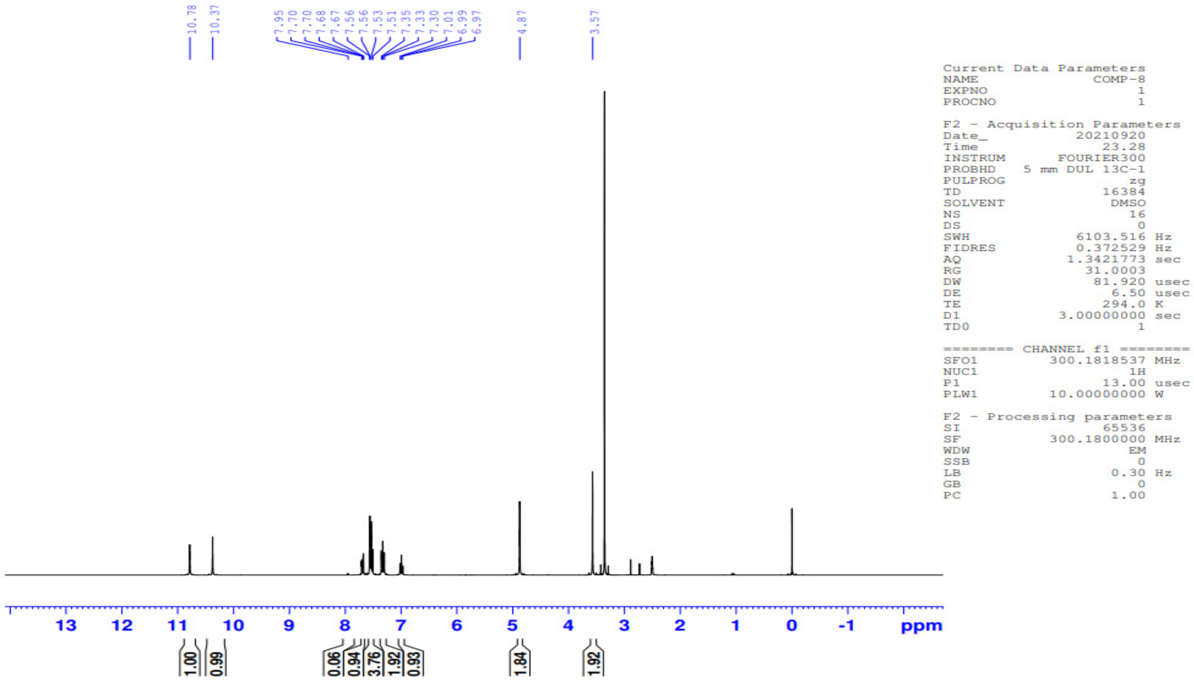

Figure S18. ^{1}HNMR 3h:

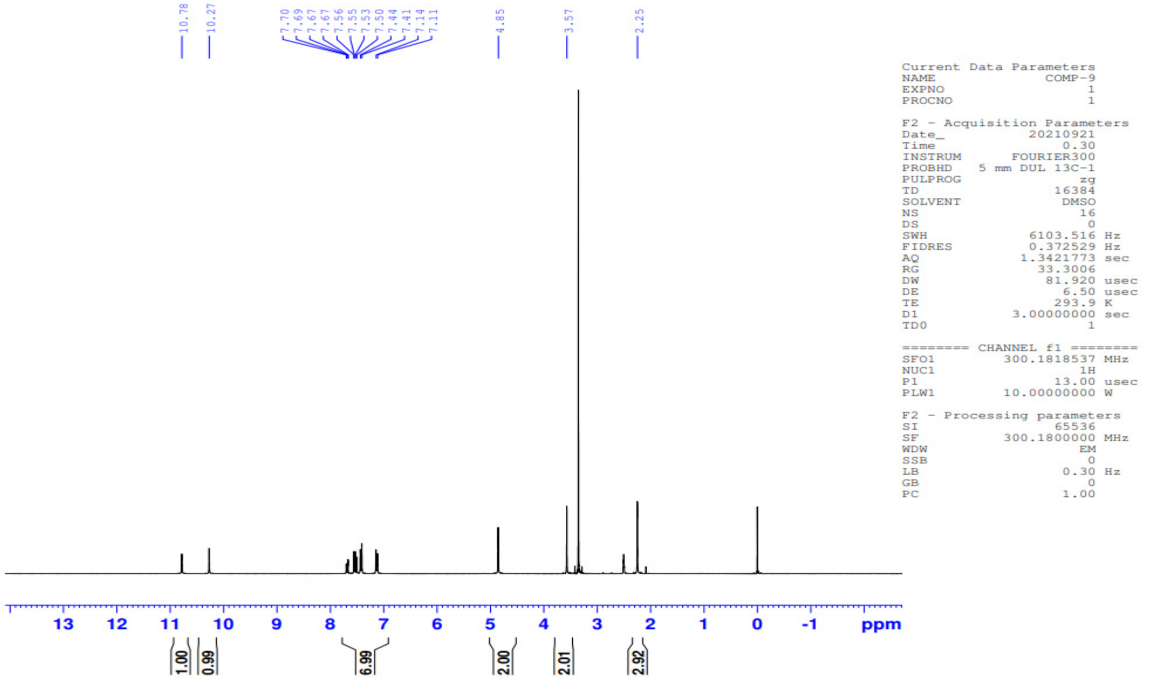

Figure S19. ^{1}**HNMR 3i:**

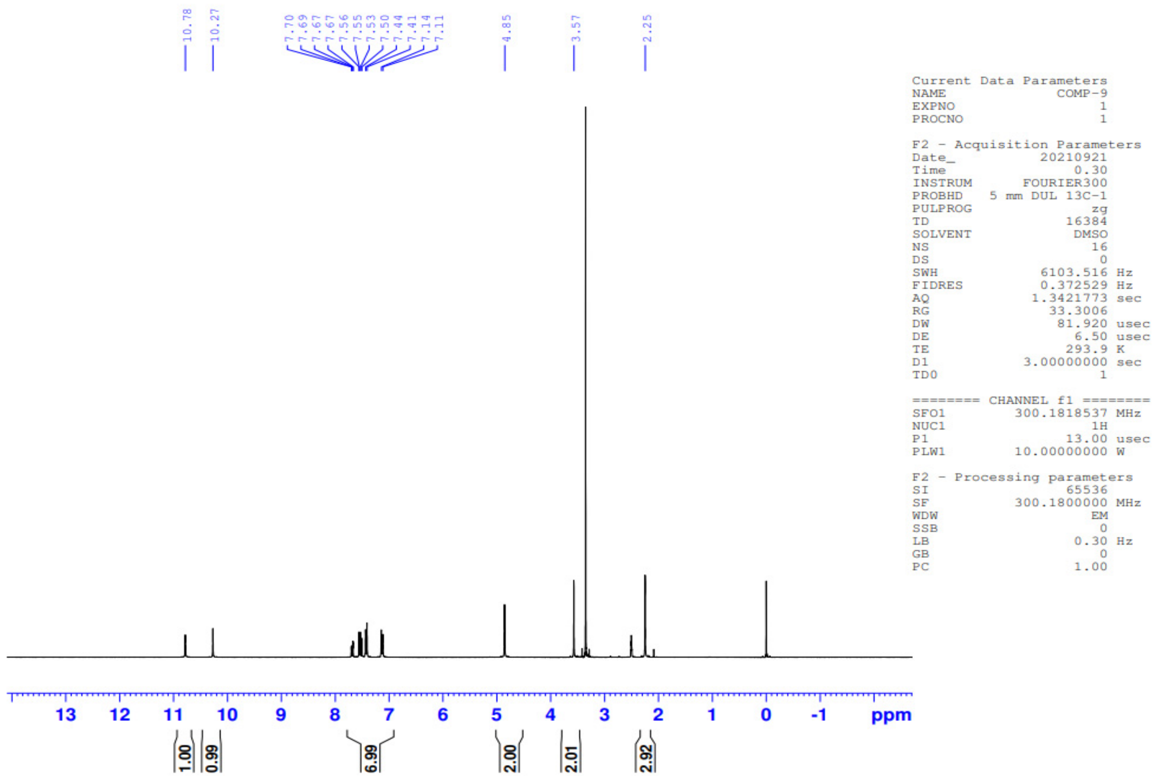

Figure S20. ^{1}**HNMR 3j:**

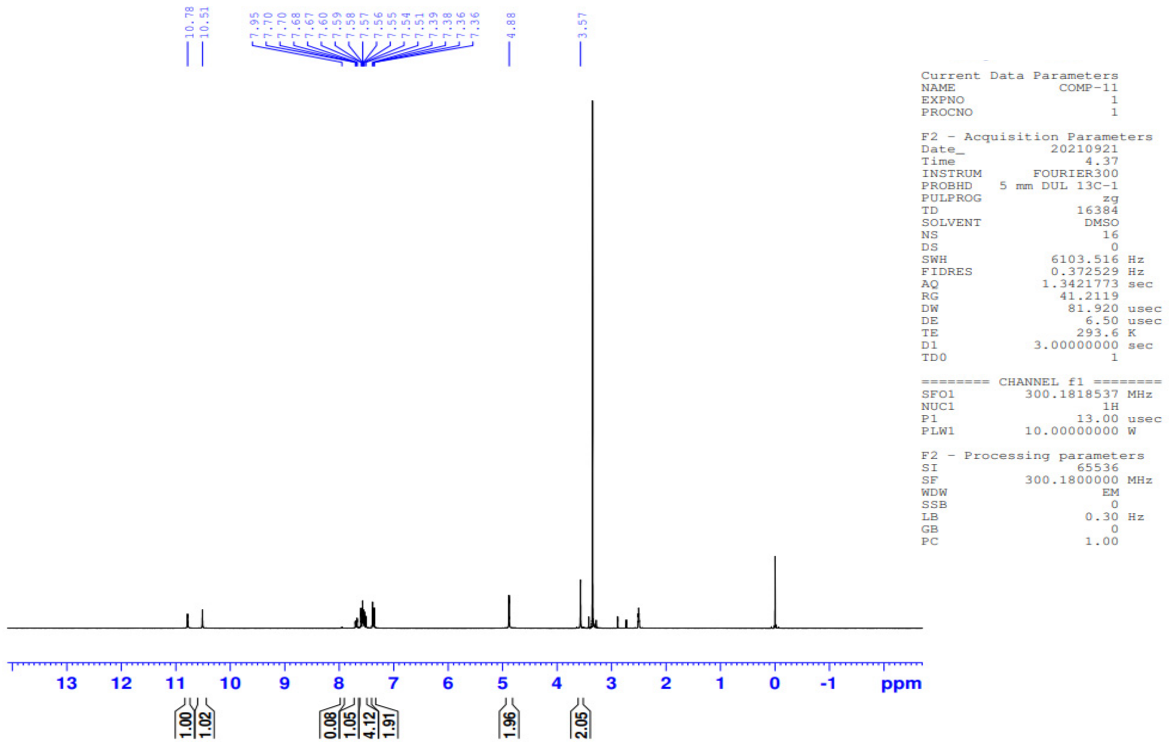

Figure S21. ^{13}CNMR-3a:

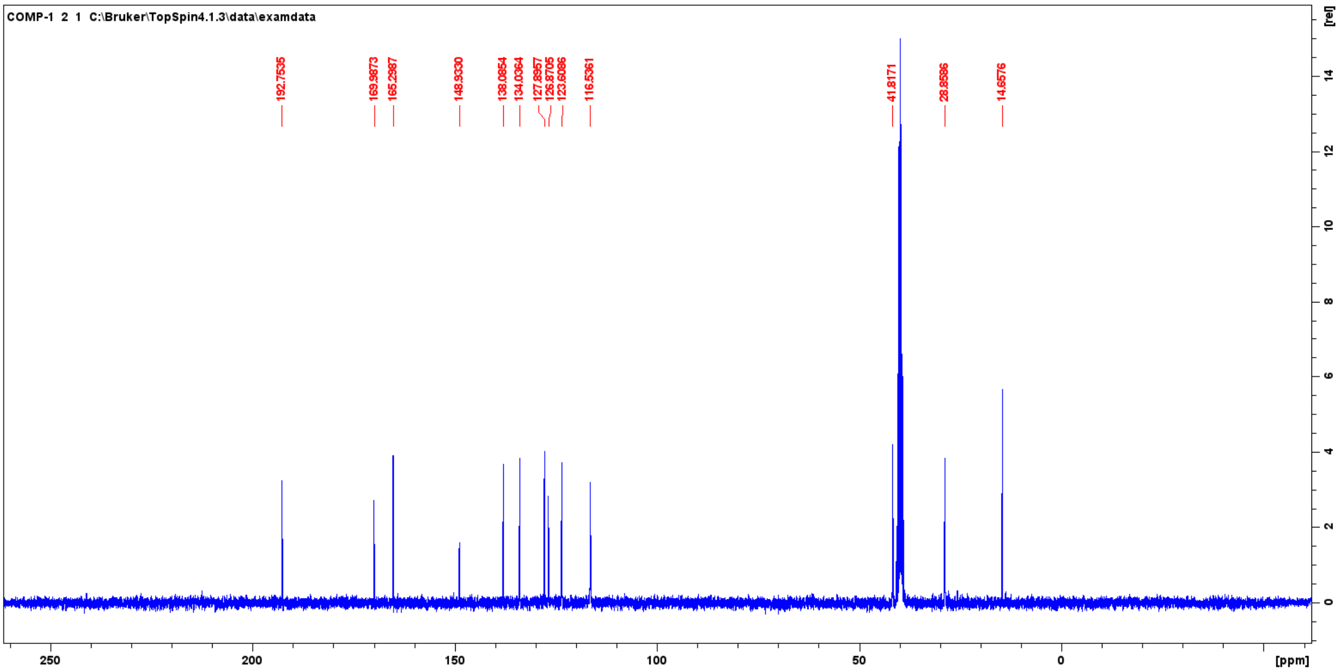

Figure S22. ^{13}CNMR-3b:

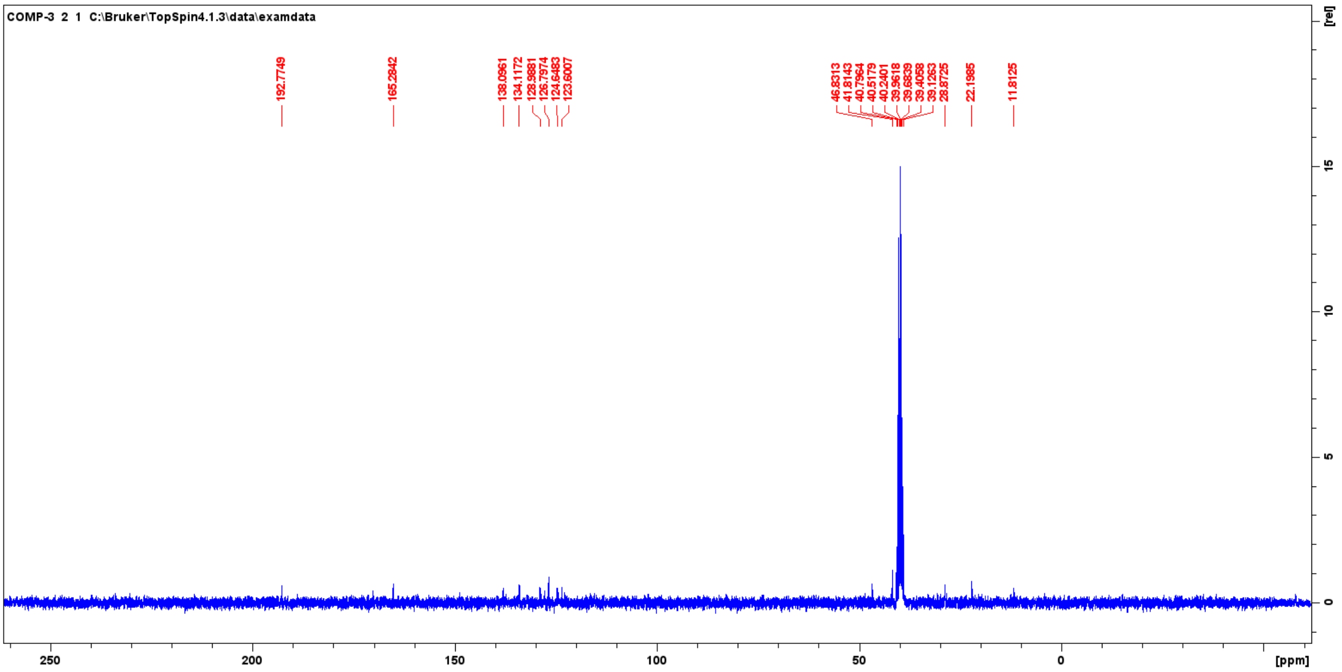

Figure S23. ^{13}CNMR-3c:

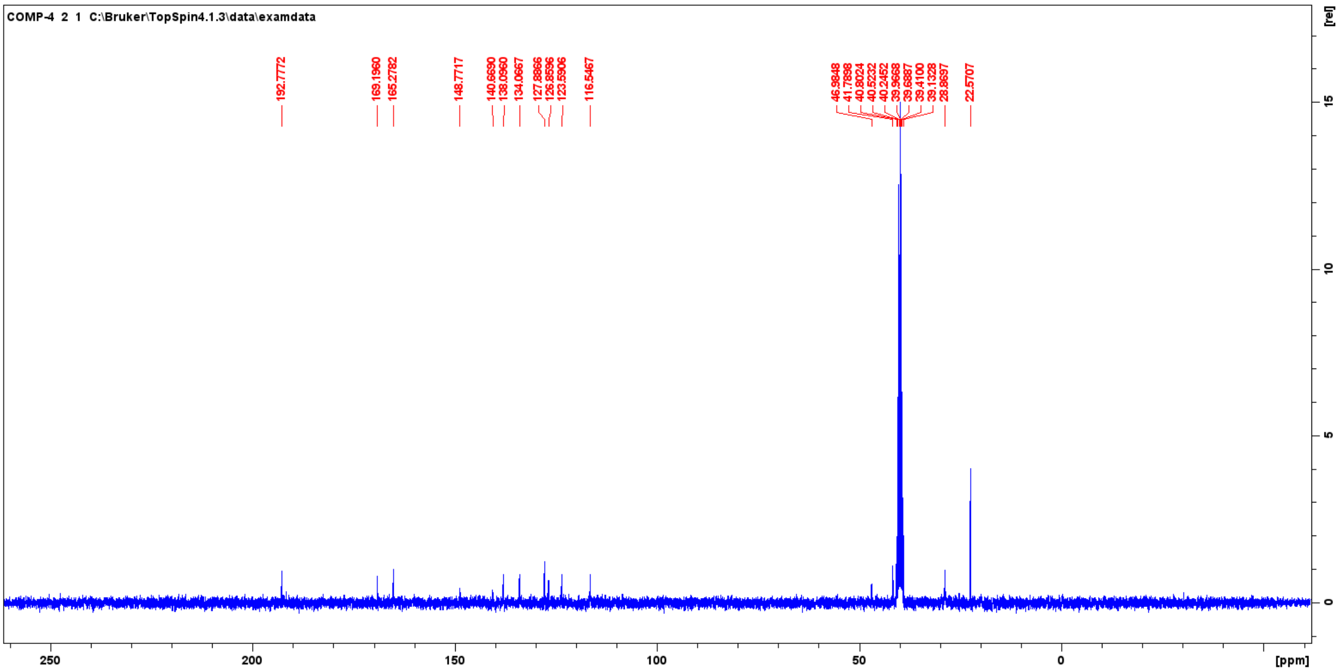

Figure S24. ^{13}CNMR-3e:

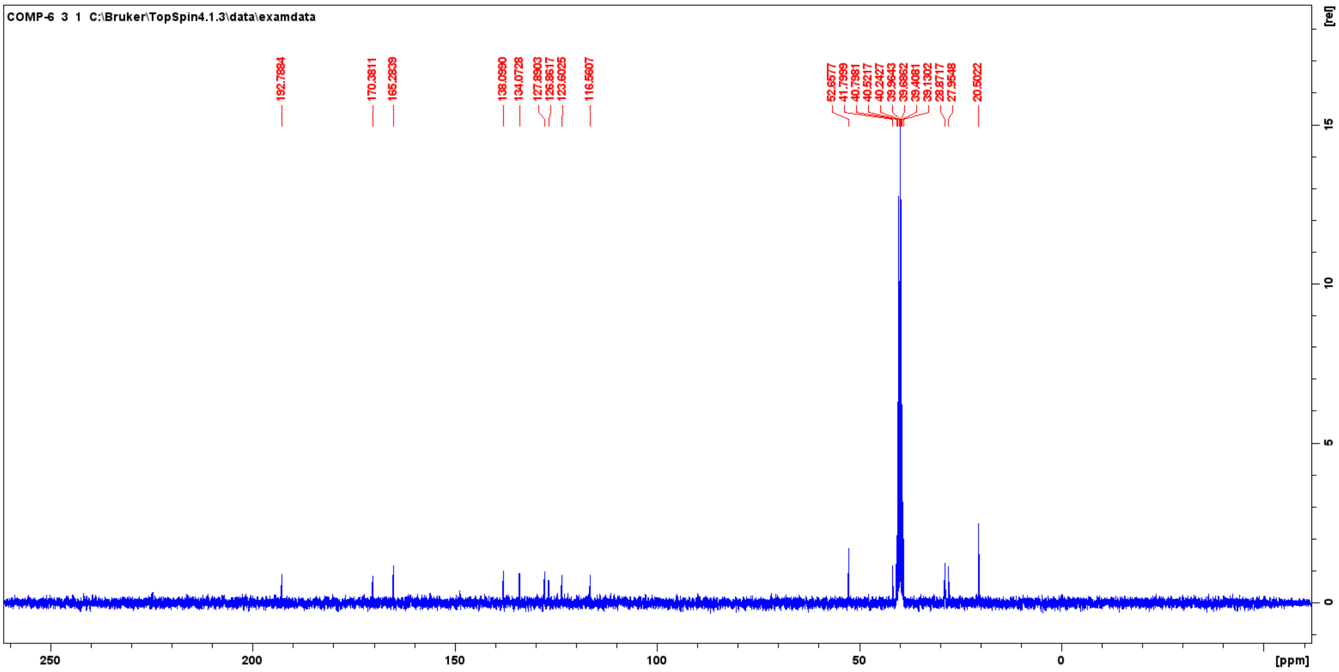

Figure S25. ^{13}**CNMR-3f:**

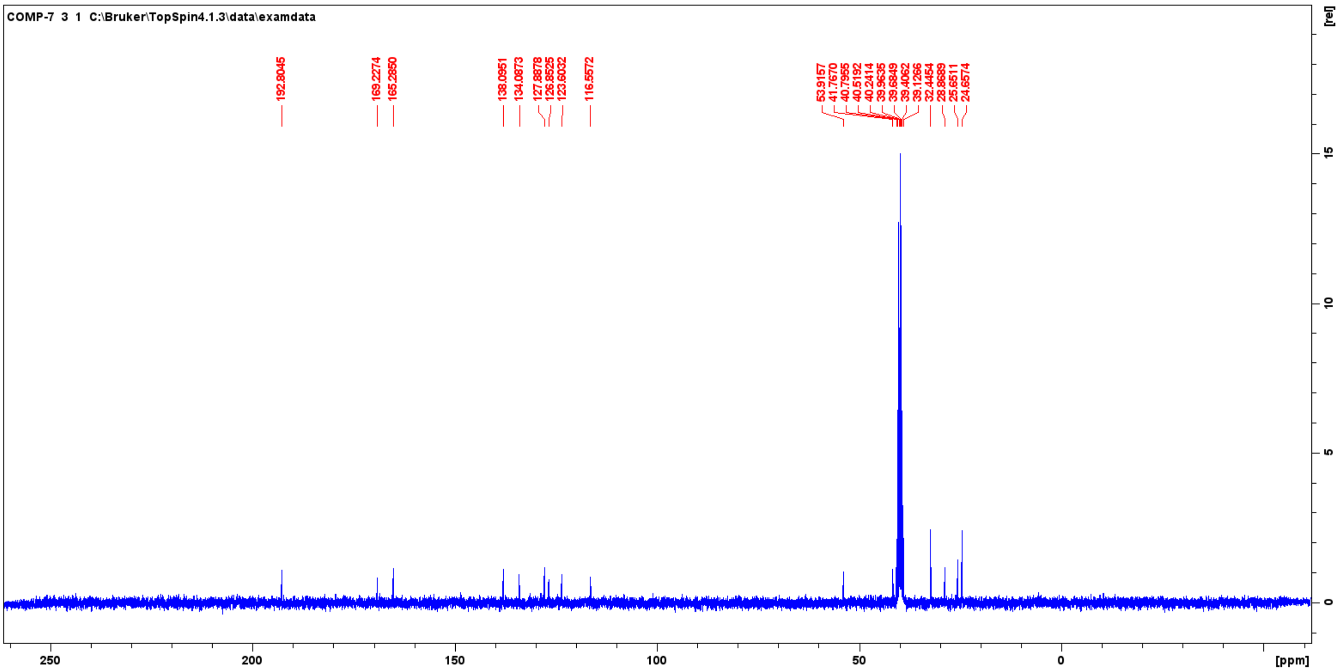

Figure S26. ^{13}**CNMR-3g:**

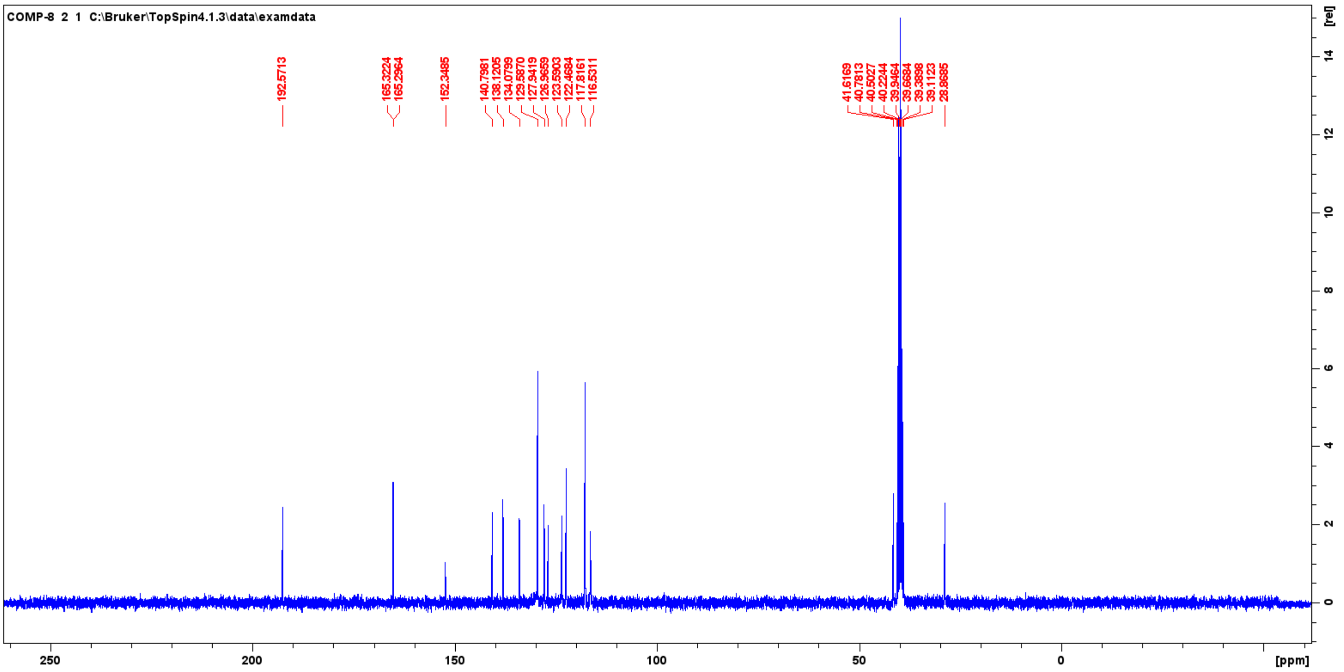

Figure S27. ^{13}CNMR-3h:

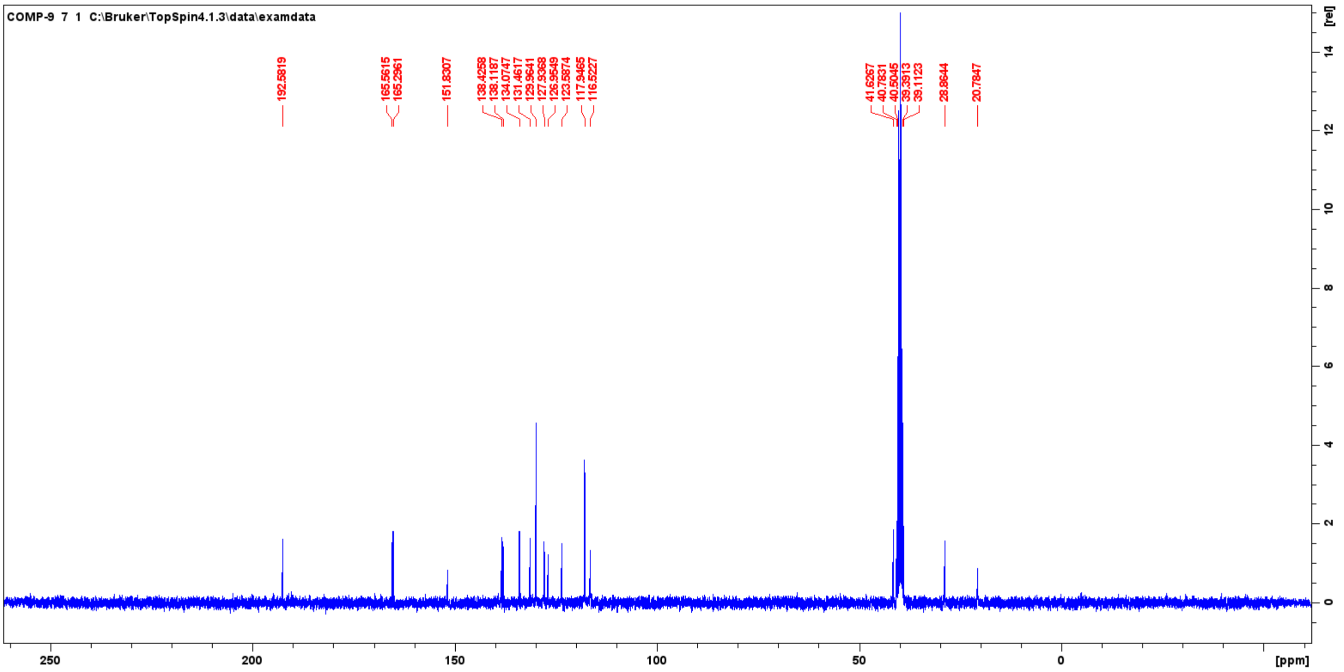

Figure S28. ^{13}CNMR-3i:

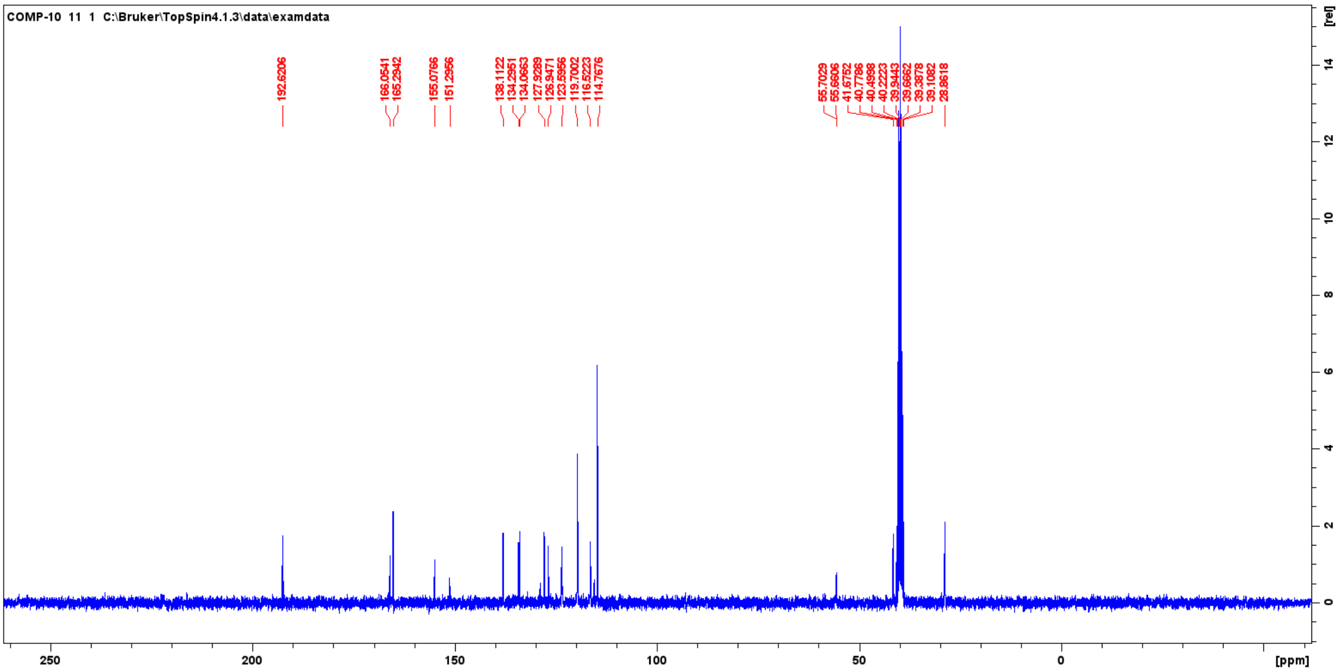

Figure S29. ^{13}CNMR-3j

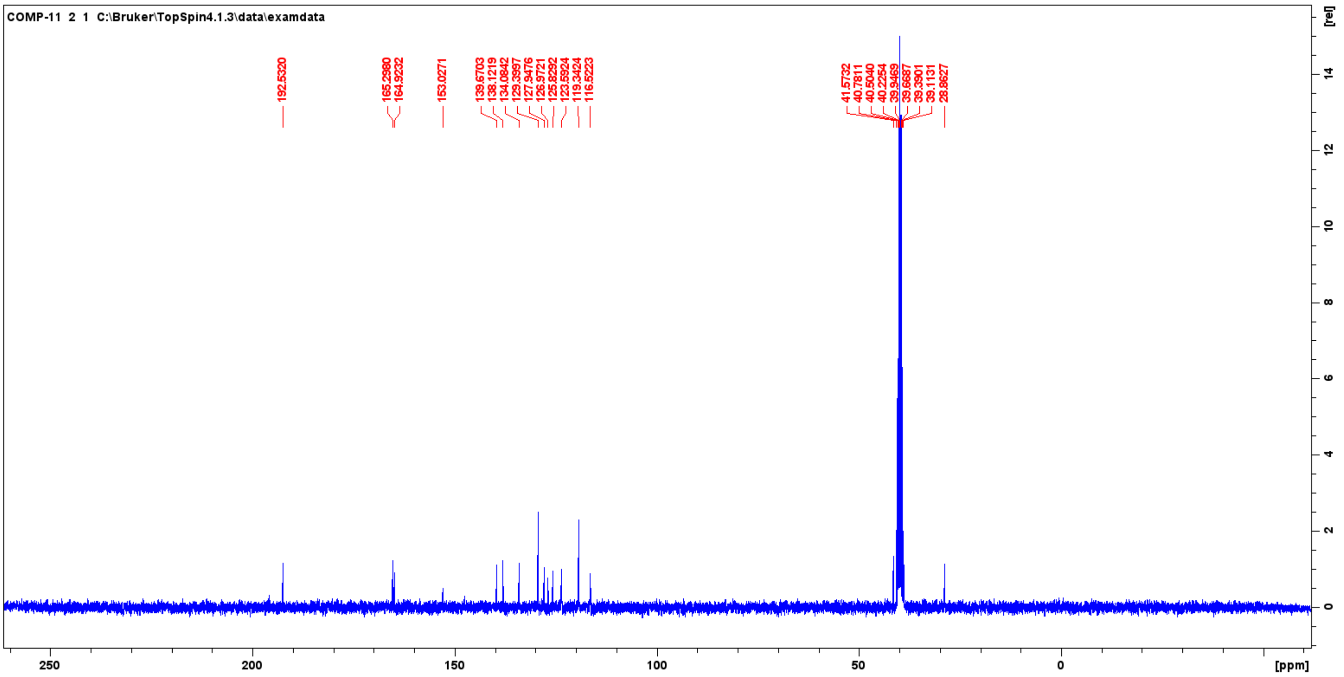

Figure S30. IR spectrum of 3a:

DOPNALAB

	Item	Value
	Acquired Date&Time	11.03.2022 15:25:51
	Acquired by	System Administrator
	Filename	C:\Users\dopnalab\Desktop\sazan\comp-1.1.ispd
	Spectrum name	comp-1.1
	Sample name	comp-1
	Sample ID	
	Option	
	Comment	
	No. of Scans	15
	Resolution	4 [cm-1]
	Apodization	Happ-Genzel

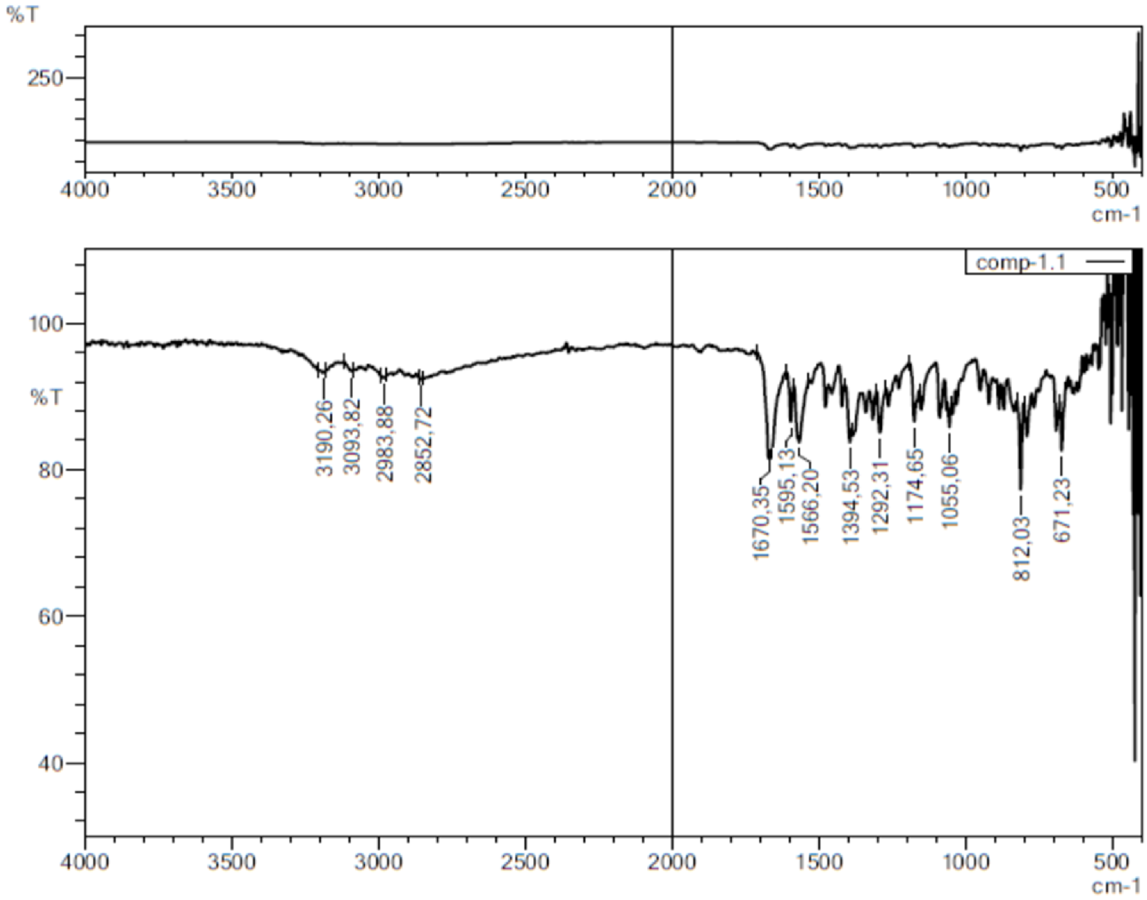

Figure S31. IR spectrum of 3b:

DOPNALAB

	Item	Value
	Acquired Date&Time	11.03.2022 15:36:20
	Acquired by	System Administrator
	Filename	C:\Users\dopnalab\Desktop\sazan\3b.1.ispd
	Spectrum name	3b.1
	Sample name	3b
	Sample ID	
	Option	
	Comment	
	No. of Scans	15
	Resolution	4 [cm-1]
	Apodization	Happ-Genzel

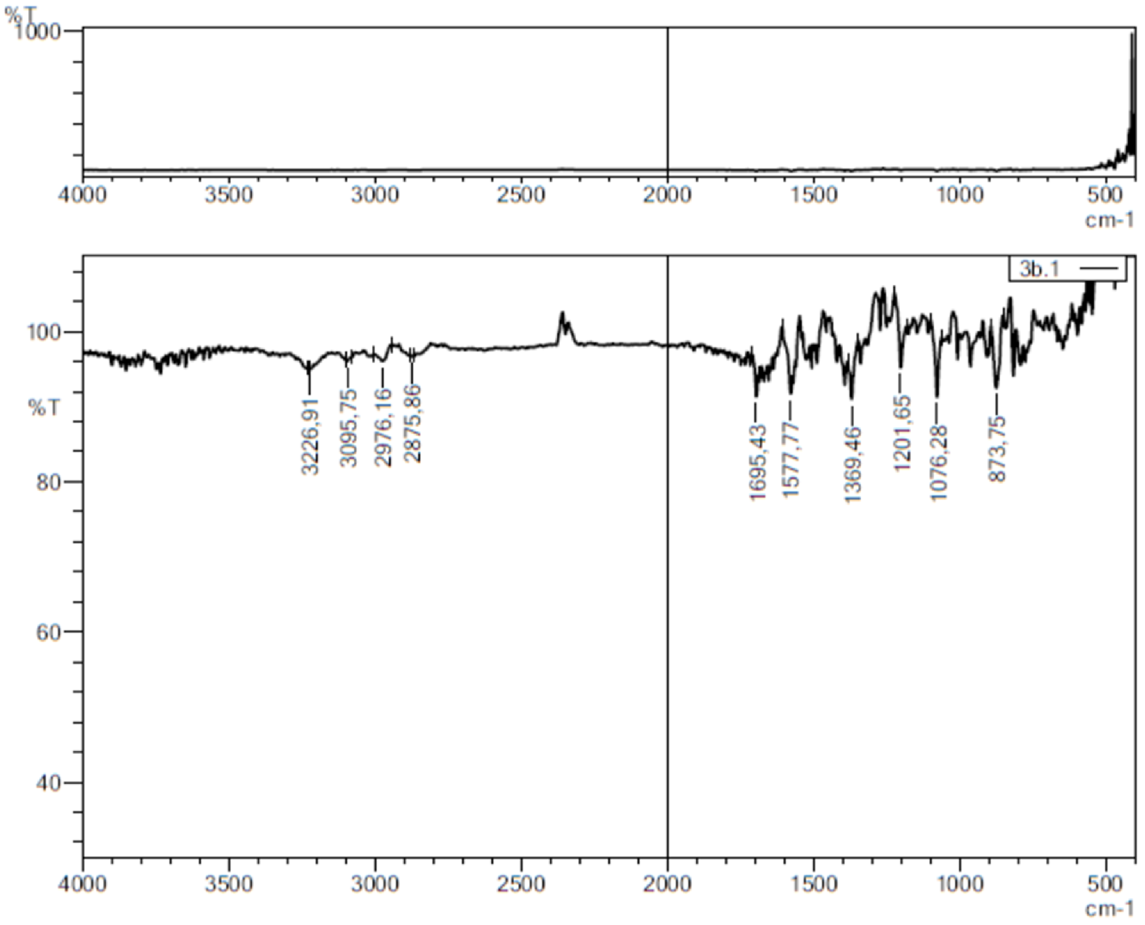

Figure S32. IR spectrum of 3c:

DOPNALAB

Item	Value
Acquired Date&Time	11.03.2022 15:45:04
Acquired by	System Administrator
Filename	C:\Users\dopnalab\Desktop\sazan\3c.1.ispd
Spectrum name	3c.1
Sample name	3c
Sample ID	
Option	
Comment	
No. of Scans	15
Resolution	4 [cm-1]
Apodization	Happ-Genzel

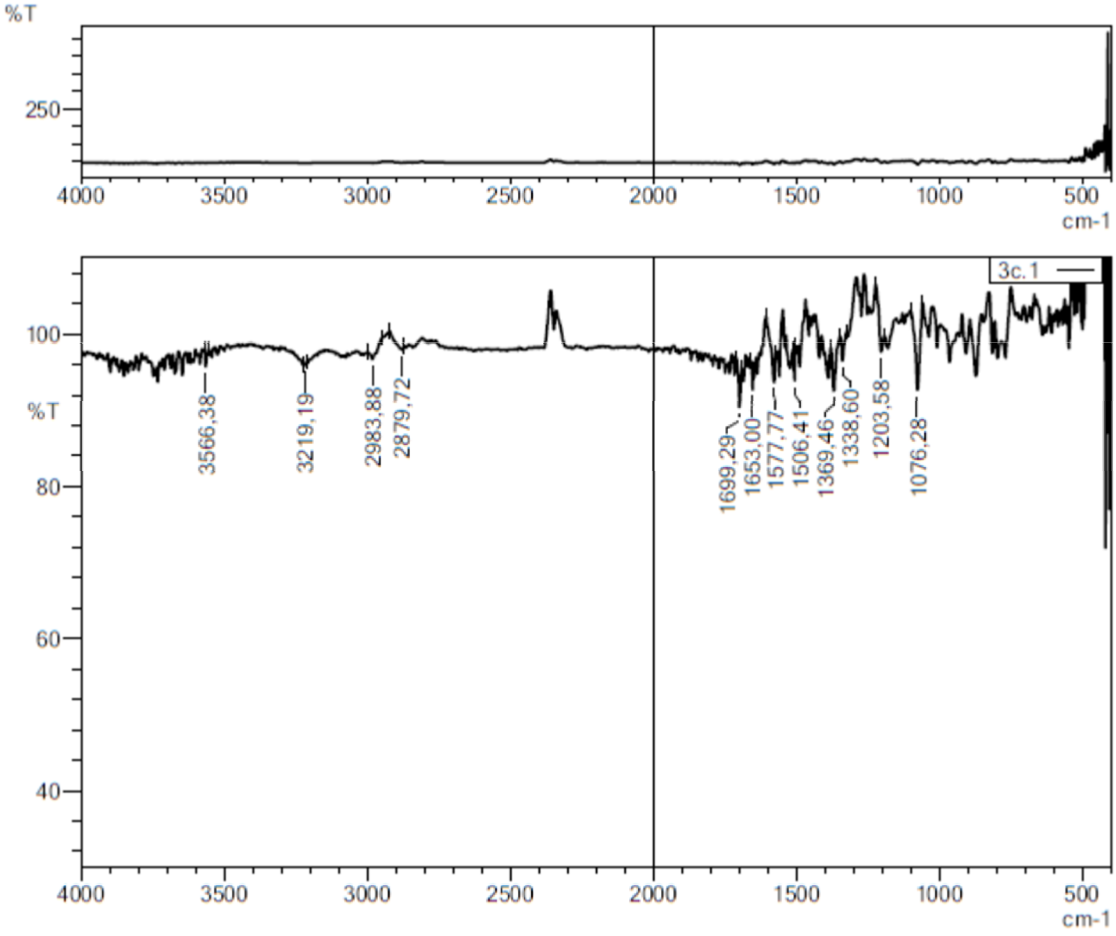

Figure S33. IR spectrum of 3d:

DOPNALAB

Item	Value
Acquired Date&Time	11.03.2022 15:55:10
Acquired by	System Administrator
Filename	C:\Users\dopnalab\Desktop\sazan\3d.1.ispd
Spectrum name	3d.1
Sample name	3d
Sample ID	
Option	
Comment	
No. of Scans	15
Resolution	4 [cm-1]
Apodization	Happ-Genzel

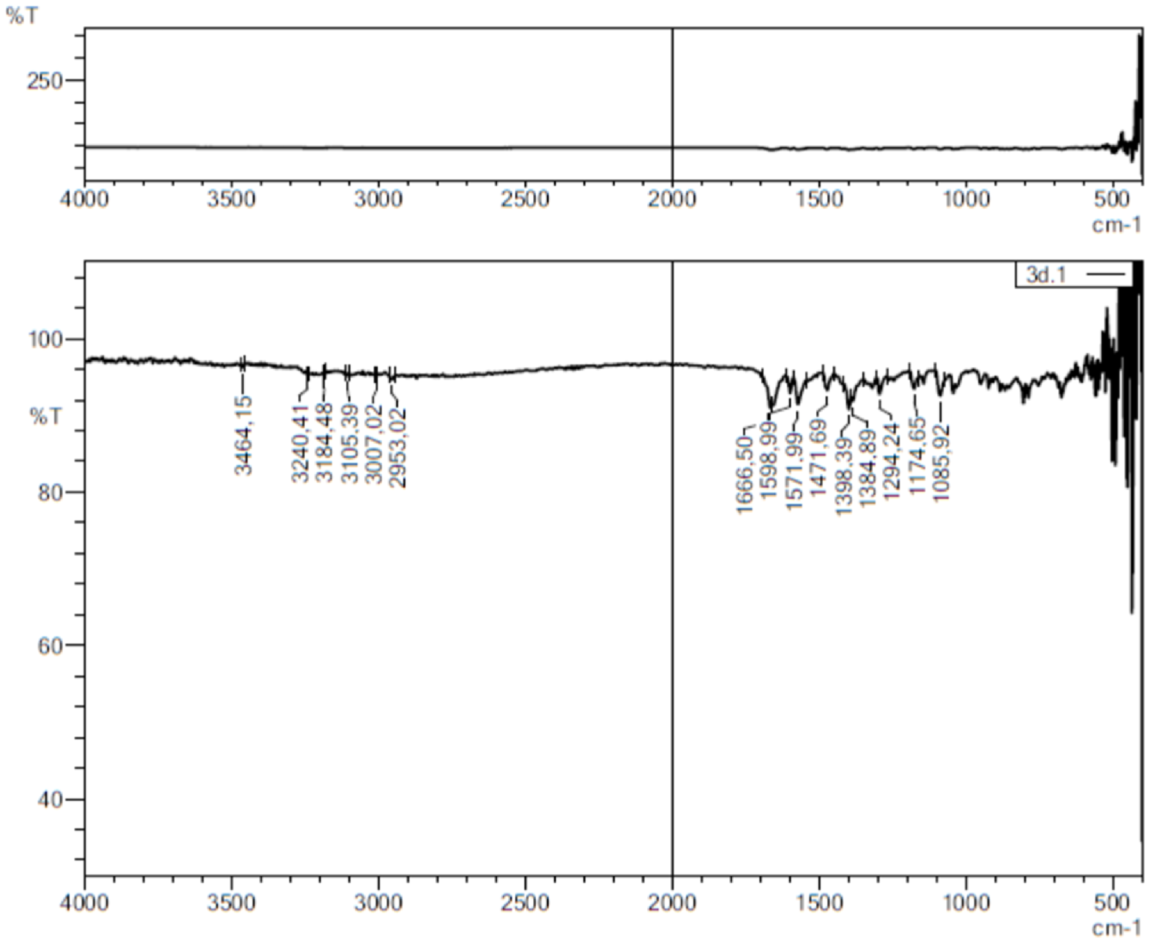

Figure S34. IR spectrum of 3e:

DOPNALAB

Item	Value
Acquired Date&Time	11.03.2022 16:02:59
Acquired by	System Administrator
Filename	C:\Users\dopnalab\Desktop\sazan\3e.1.ispd
Spectrum name	3e.1
Sample name	3e
Sample ID	
Option	
Comment	
No. of Scans	15
Resolution	4 [cm-1]
Apodization	Happ-Genzel

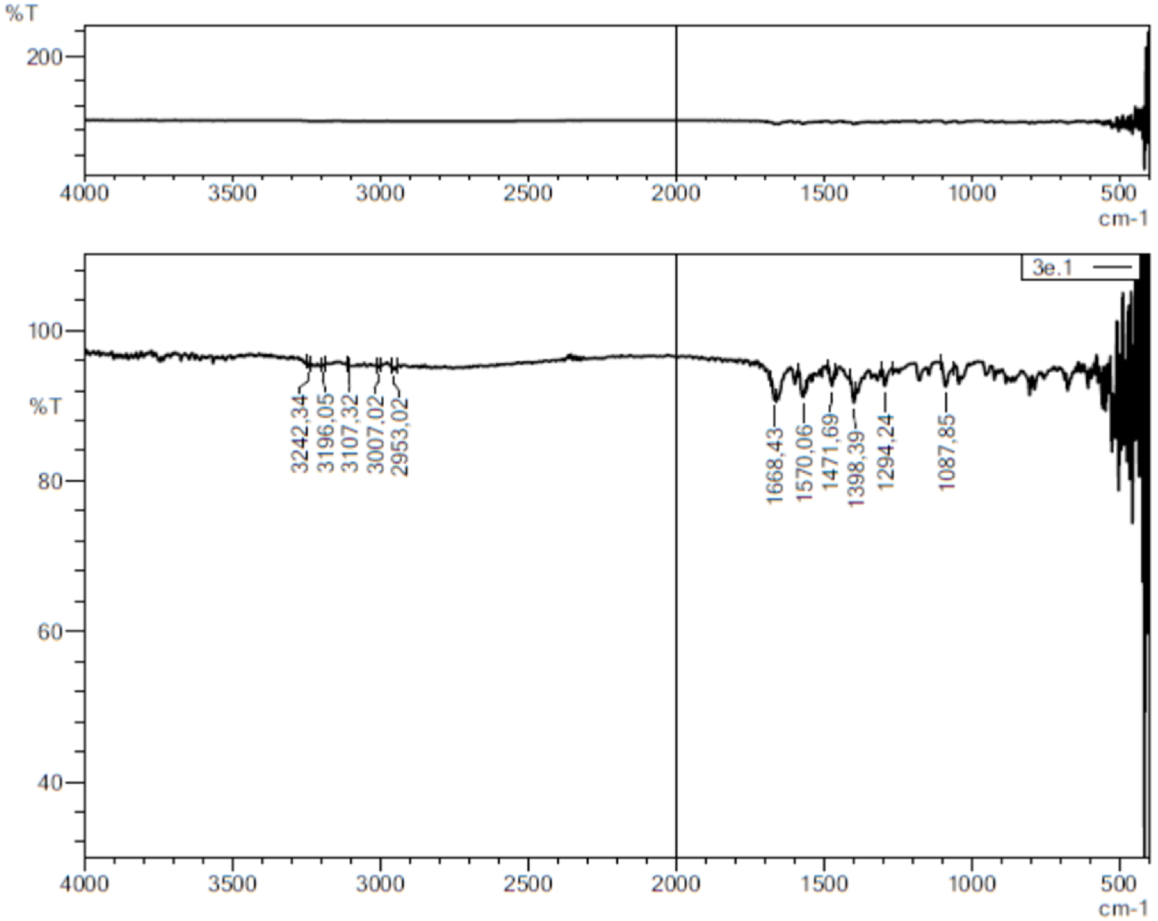

Figure S35. IR spectrum of 3f:

DOPNALAB

Item	Value
Acquired Date&Time	11.03.2022 16:10:03
Acquired by	System Administrator
Filename	C:\Users\dopnalab\Desktop\sazan\3f.1.ispd
Spectrum name	3f.1
Sample name	3f
Sample ID	
Option	
Comment	
No. of Scans	15
Resolution	4 [cm-1]
Apodization	Happ-Genzel

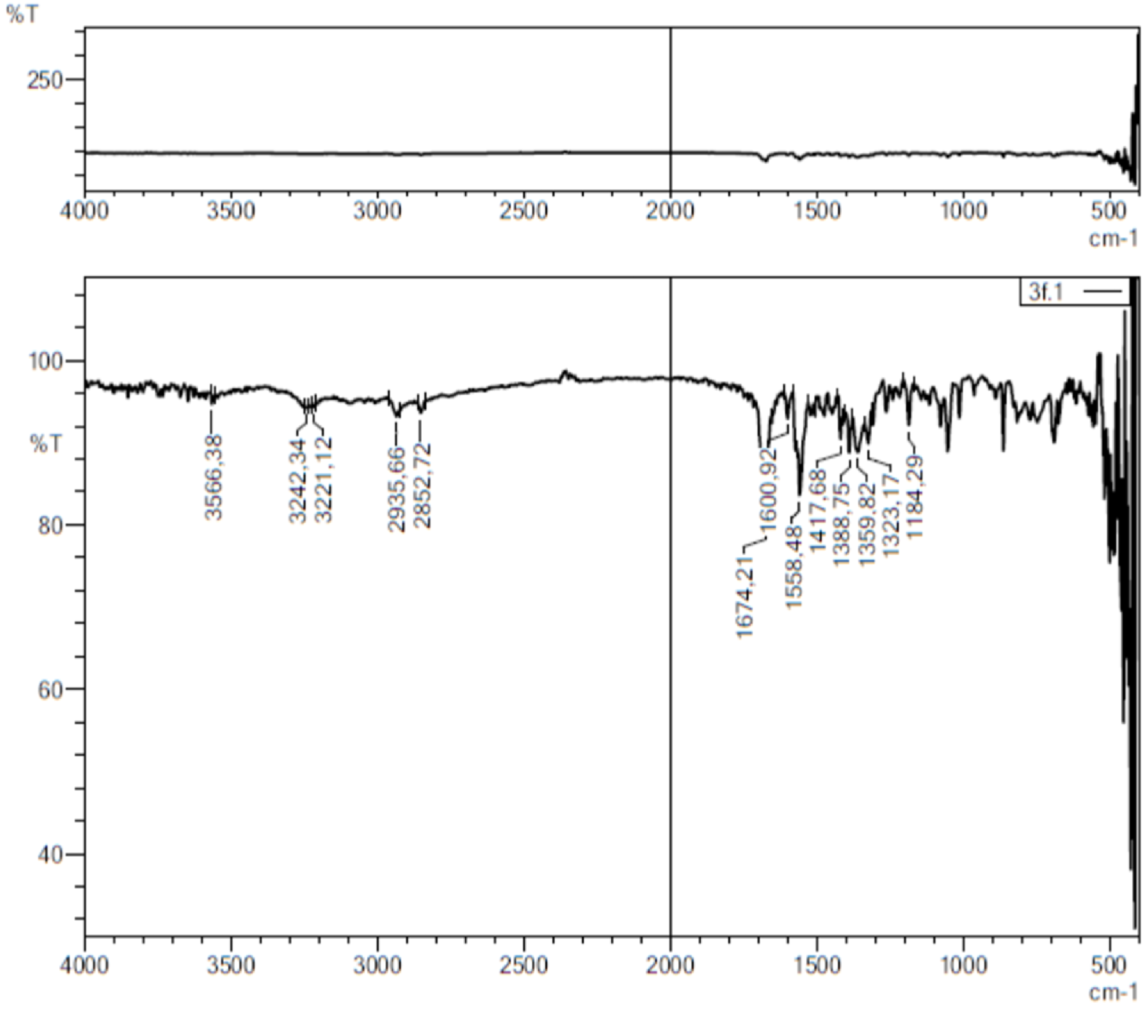

Figure S36. IR spectrum of 3g:

DOPNALAB

Item	Value
Acquired Date&Time	11.03.2022 16:20:17
Acquired by	System Administrator
Filename	C:\Users\dopnalab\Desktop\sazan\3g.1.ispd
Spectrum name	3g.1
Sample name	3g
Sample ID	
Option	
Comment	
No. of Scans	15
Resolution	4 [cm-1]
Apodization	Happ-Genzel

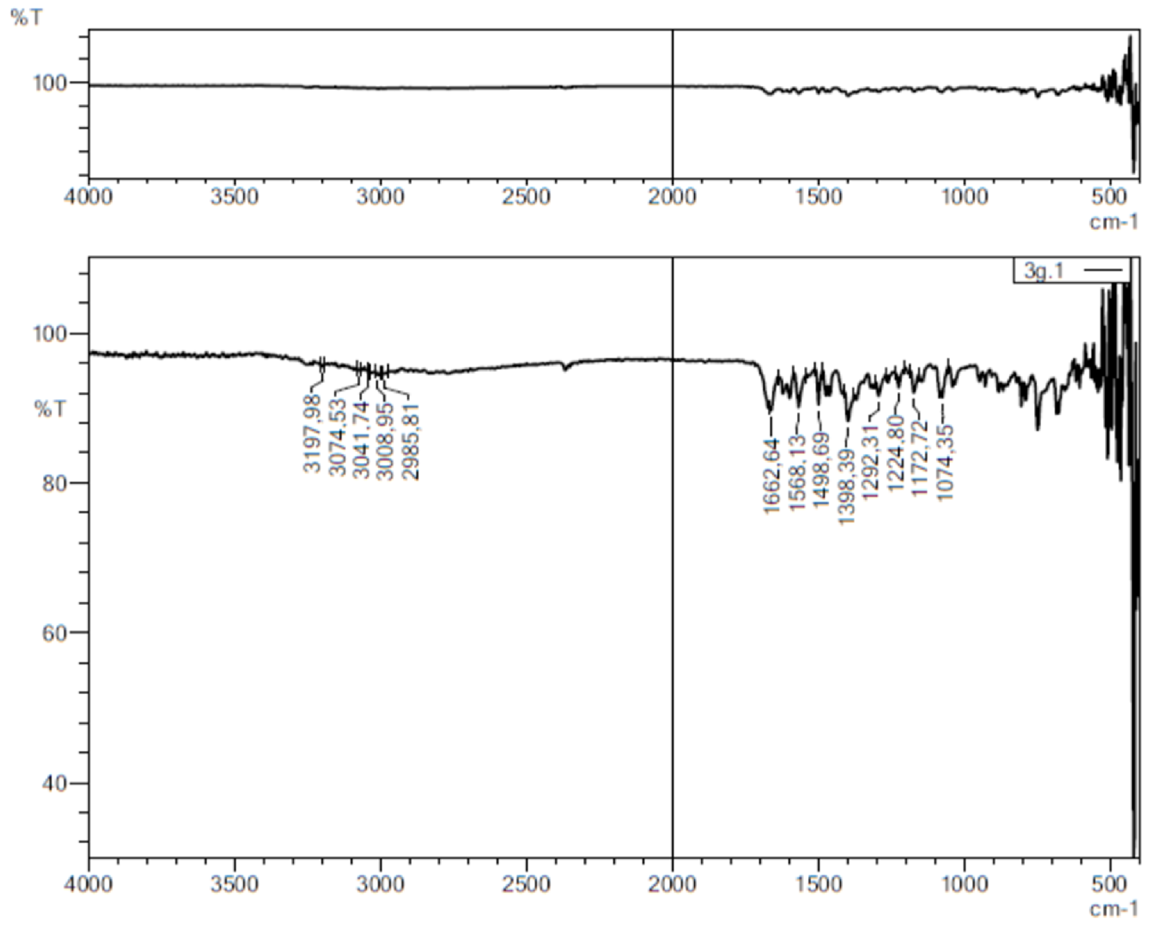

Figure S37. IR spectrum of 3h:

DOPNALAB

Item	Value
Acquired Date&Time	11.03.2022 16:28:45
Acquired by	System Administrator
Filename	C:\Users\dopnalab\Desktop\sazan\3h.1.ispd
Spectrum name	3h.1
Sample name	3h
Sample ID	
Option	
Comment	
No. of Scans	15
Resolution	4 [cm-1]
Apodization	Happ-Genzel

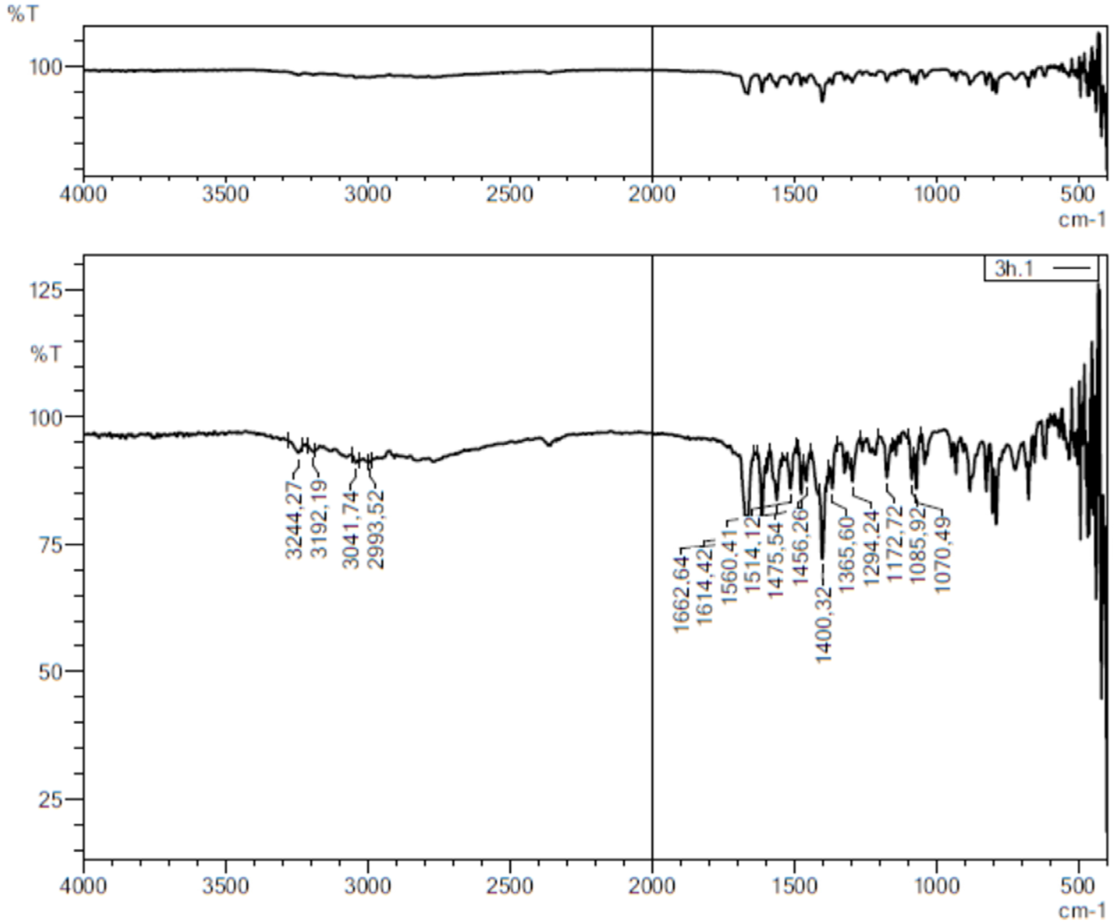

Figure S38. IR spectrum of 3i:

DOPNALAB

	Item	Value
	Acquired Date&Time	11.03.2022 16:34:51
	Acquired by	System Administrator
	Filename	C:\Users\dopnalab\Desktop\sazan\3i.1.ispd
	Spectrum name	3i.1
	Sample name	3i
	Sample ID	
	Option	
	Comment	
	No. of Scans	15
	Resolution	4 [cm-1]
	Apodization	Happ-Genzel

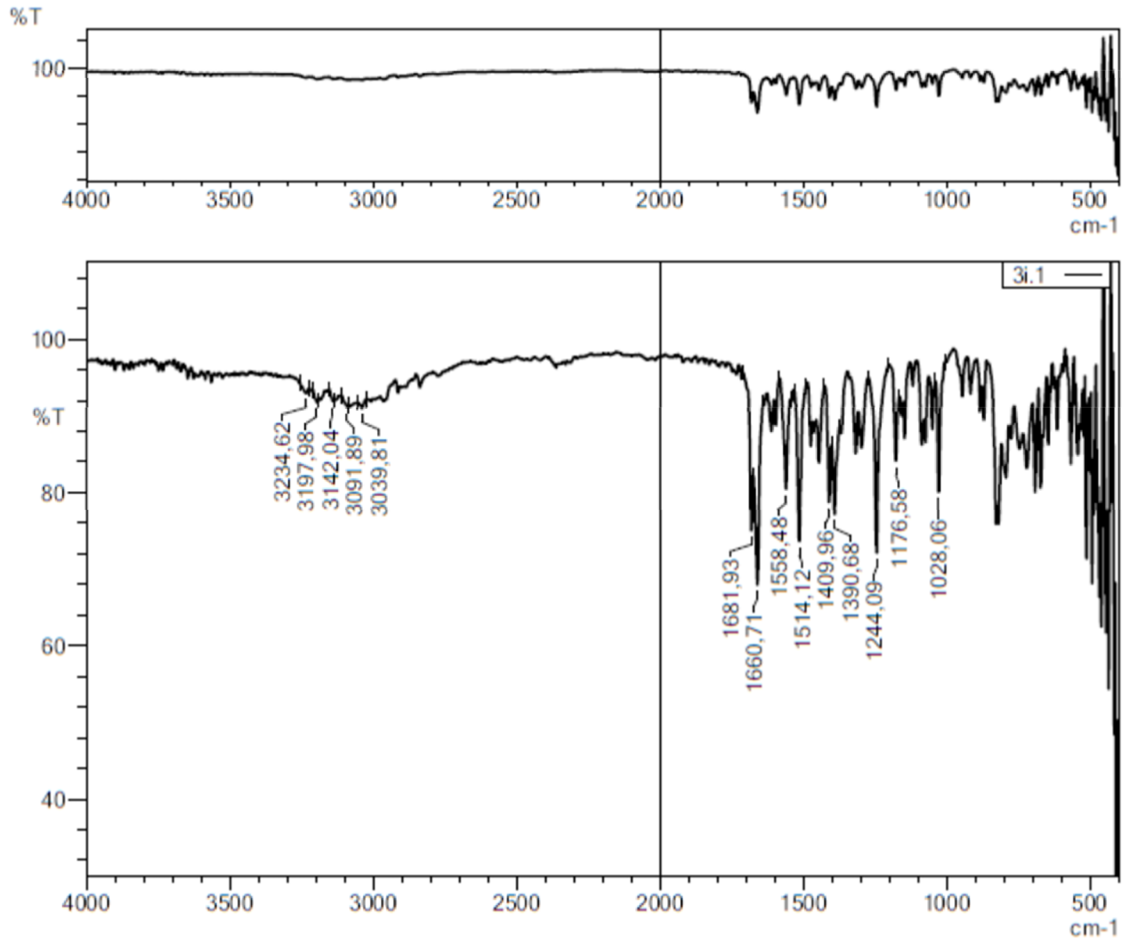

Figure S39. IR spectrum of 3j:

DOPNALAB

Item	Value
Acquired Date&Time	11.03.2022 16:39:14
Acquired by	System Administrator
Filename	C:\Users\dopnalab\Desktop\sazan\3j.1.ispd
Spectrum name	3j.1
Sample name	3j
Sample ID	
Option	
Comment	
No. of Scans	15
Resolution	4 [cm-1]
Apodization	Happ-Genzel

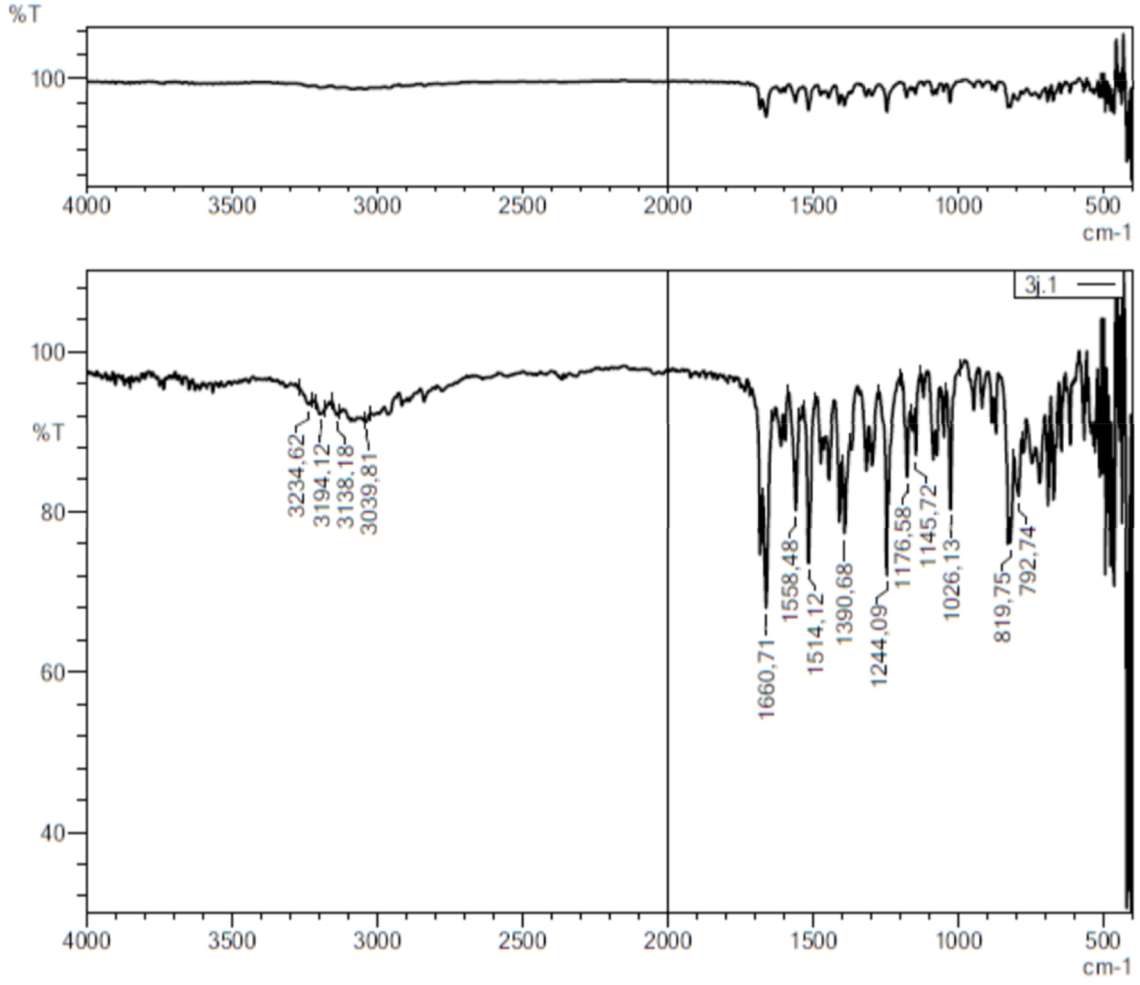

Figure S40. ^{1}**HNMR of 2a:**

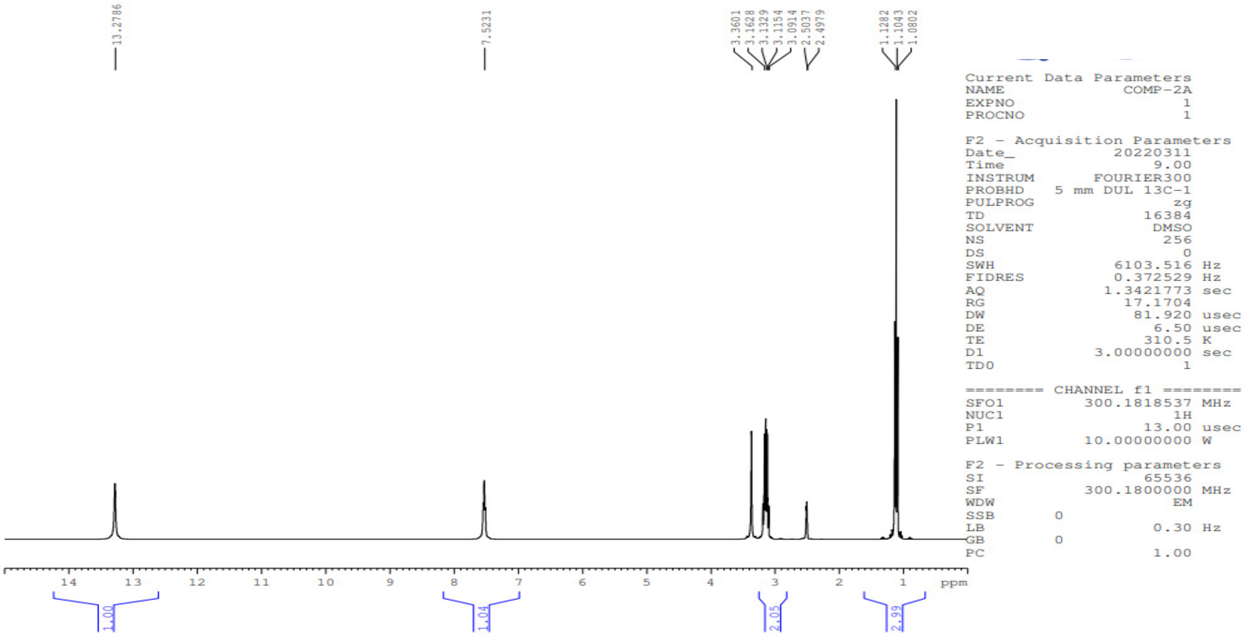

Figure S41. ^{1}**HNMR of 2b:**

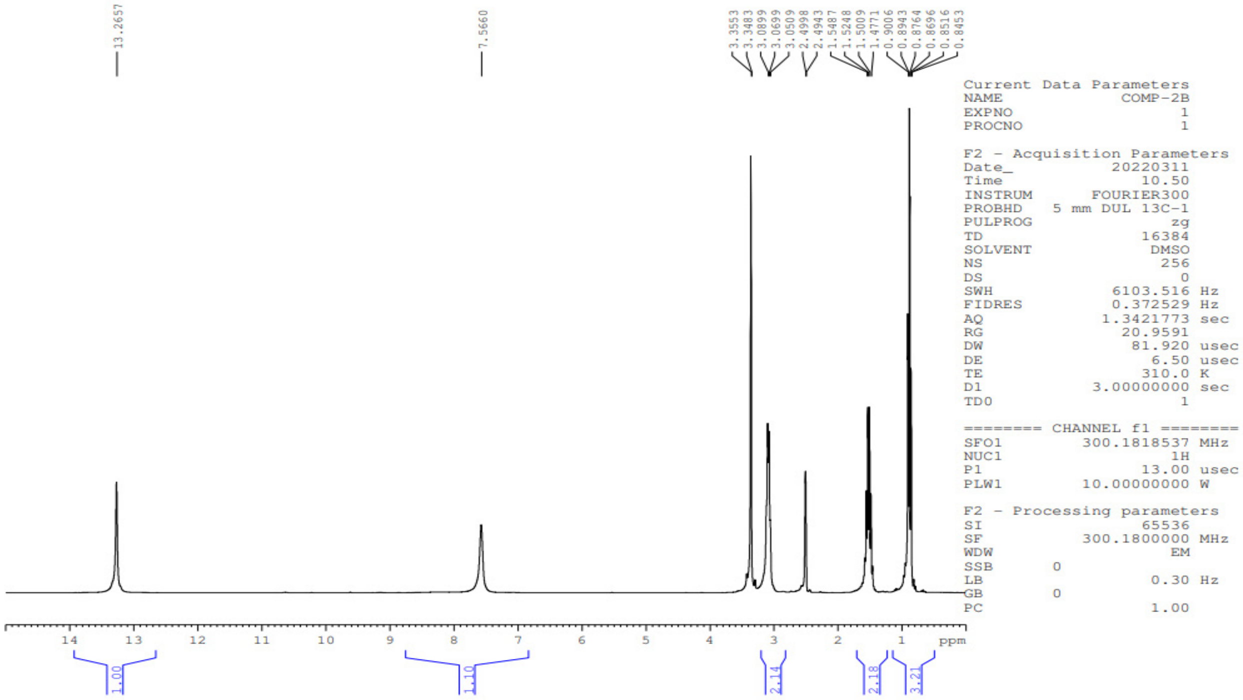

Figure S42. ^{1}**HNMR of 2c:**

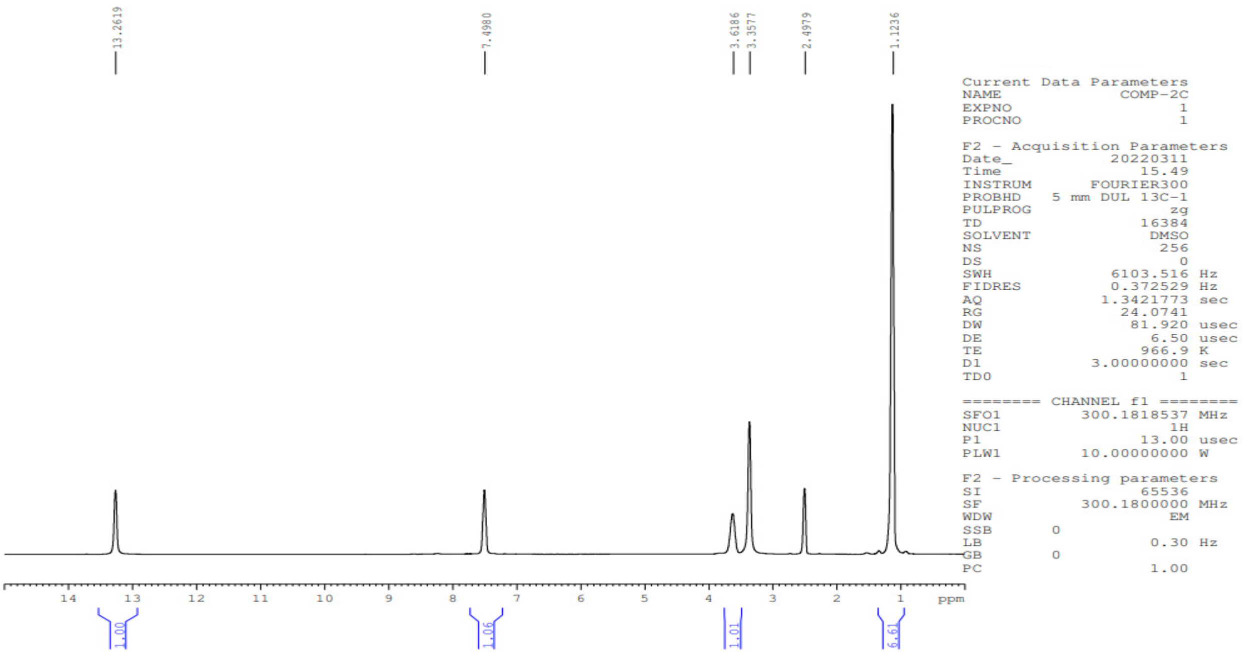

Figure S43. ^{1}**HNMR of 2d:**

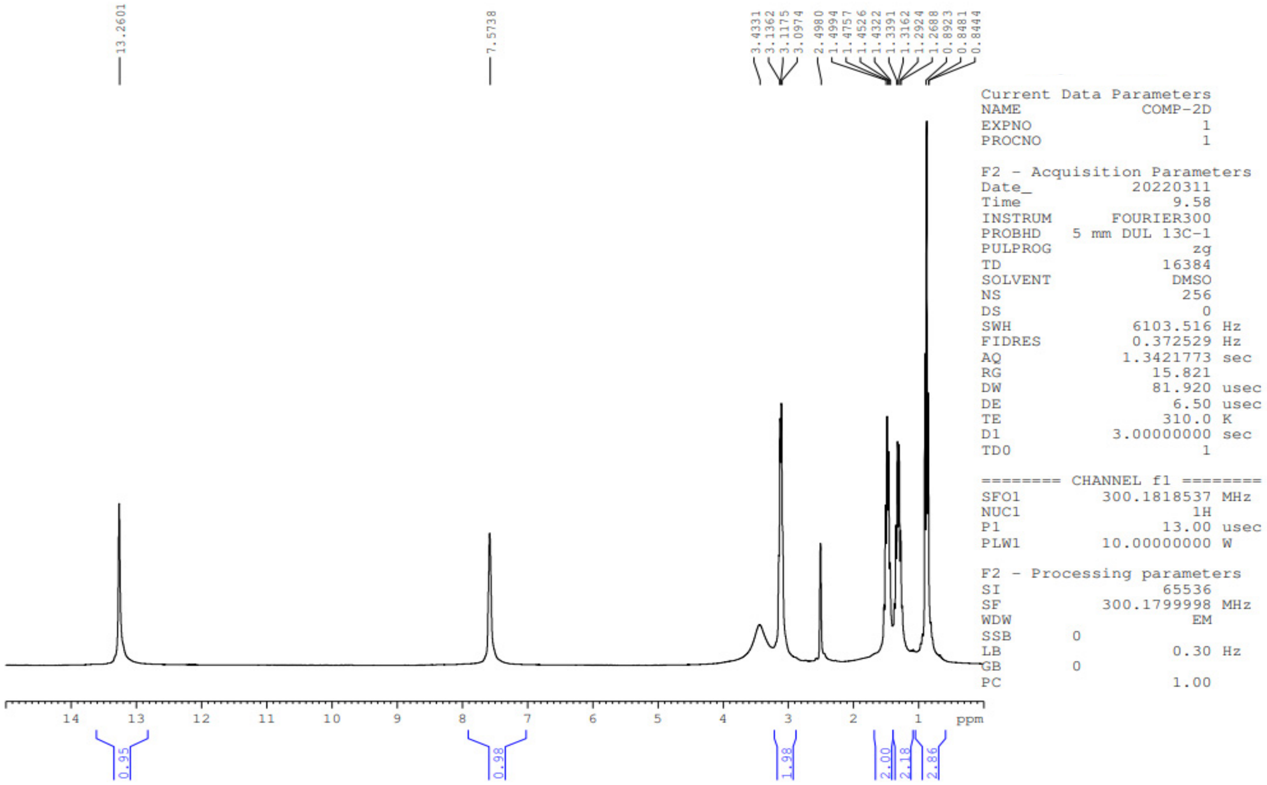

Figure S44. ^{1}HNMR of 2e:

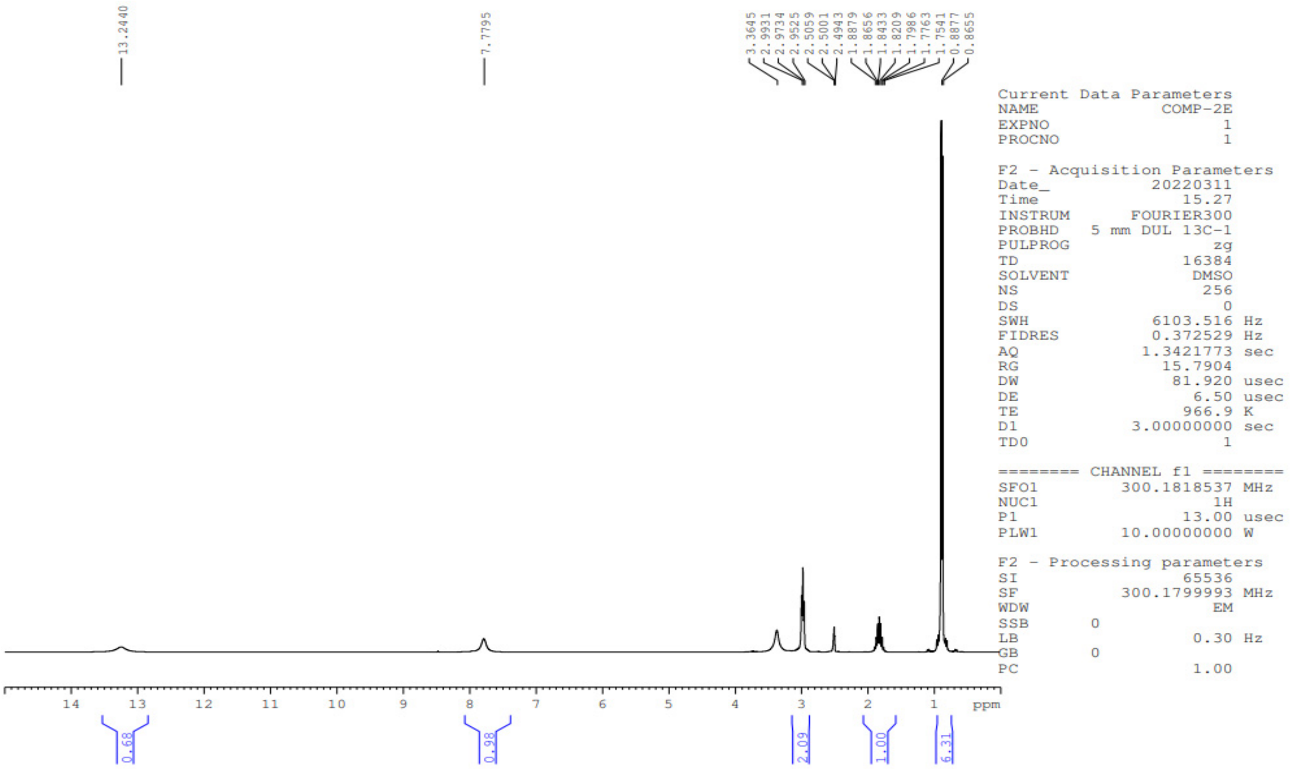

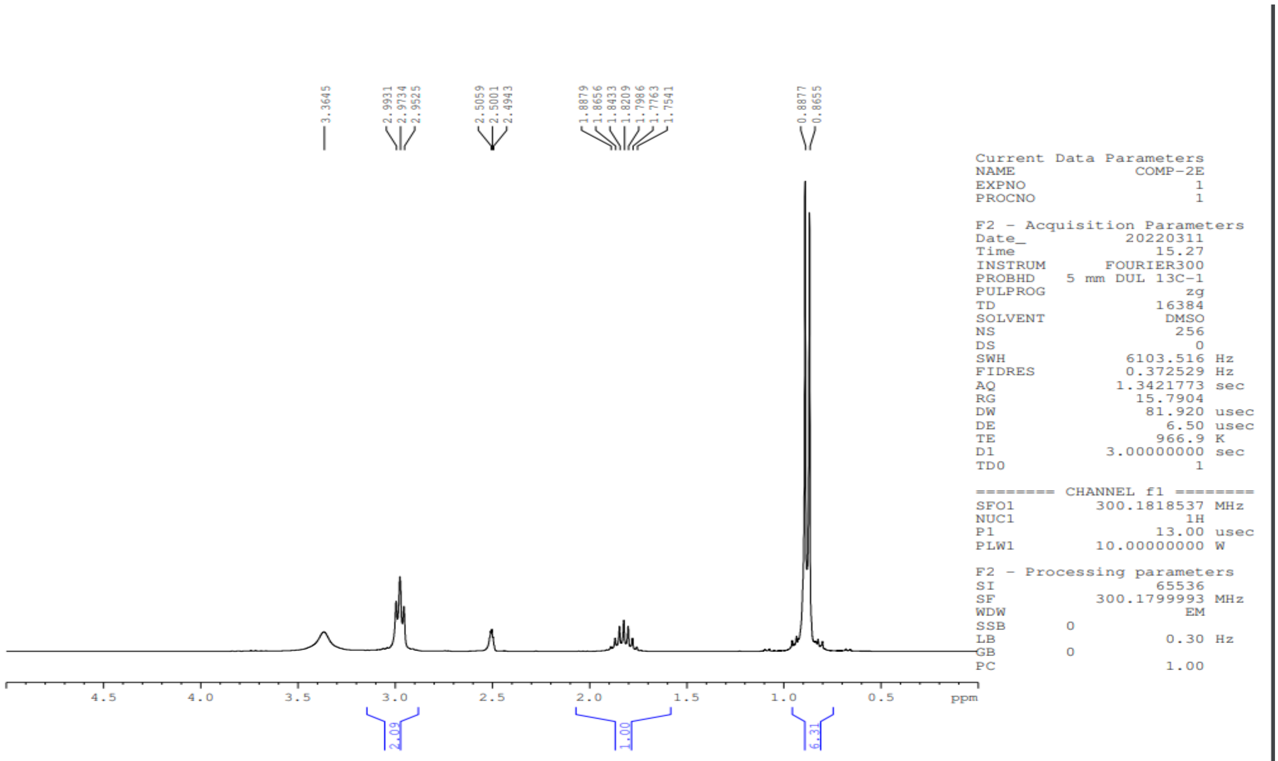

Figure S45. [1]**HNMR of 2f:**

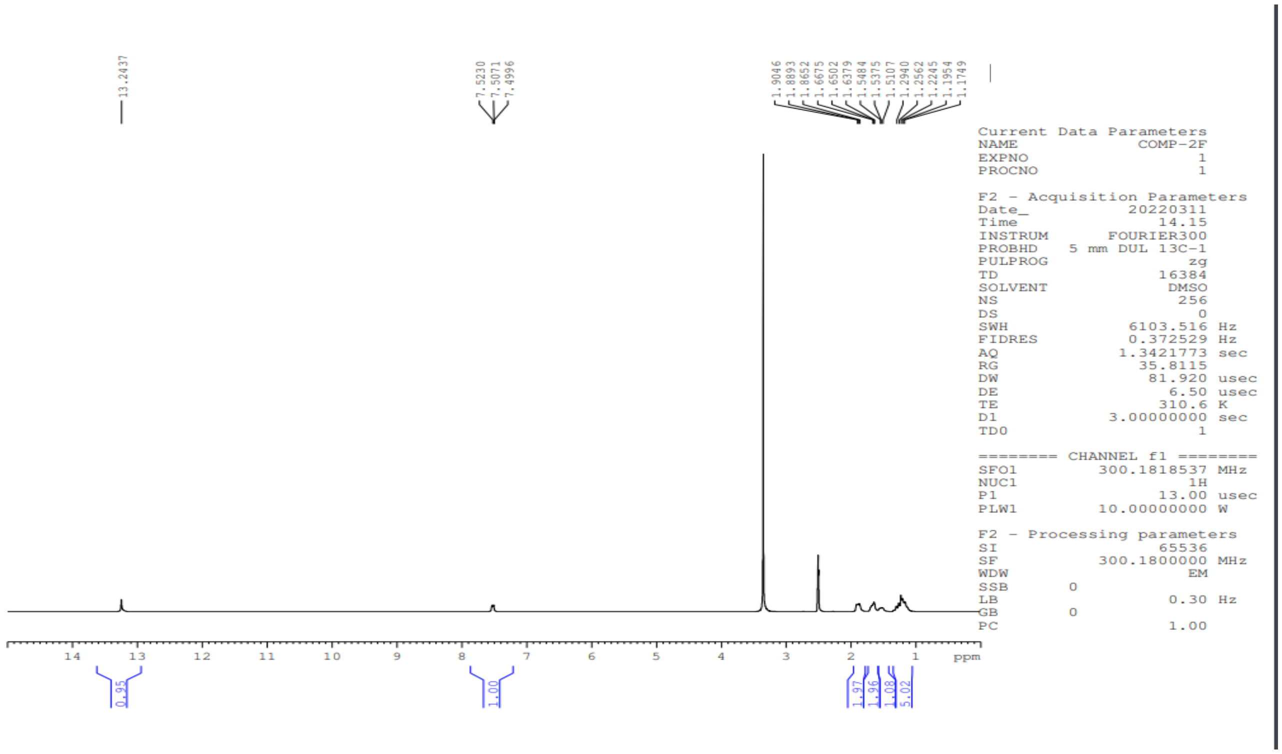

Figure S46. [1]**HNMR of 2g:**

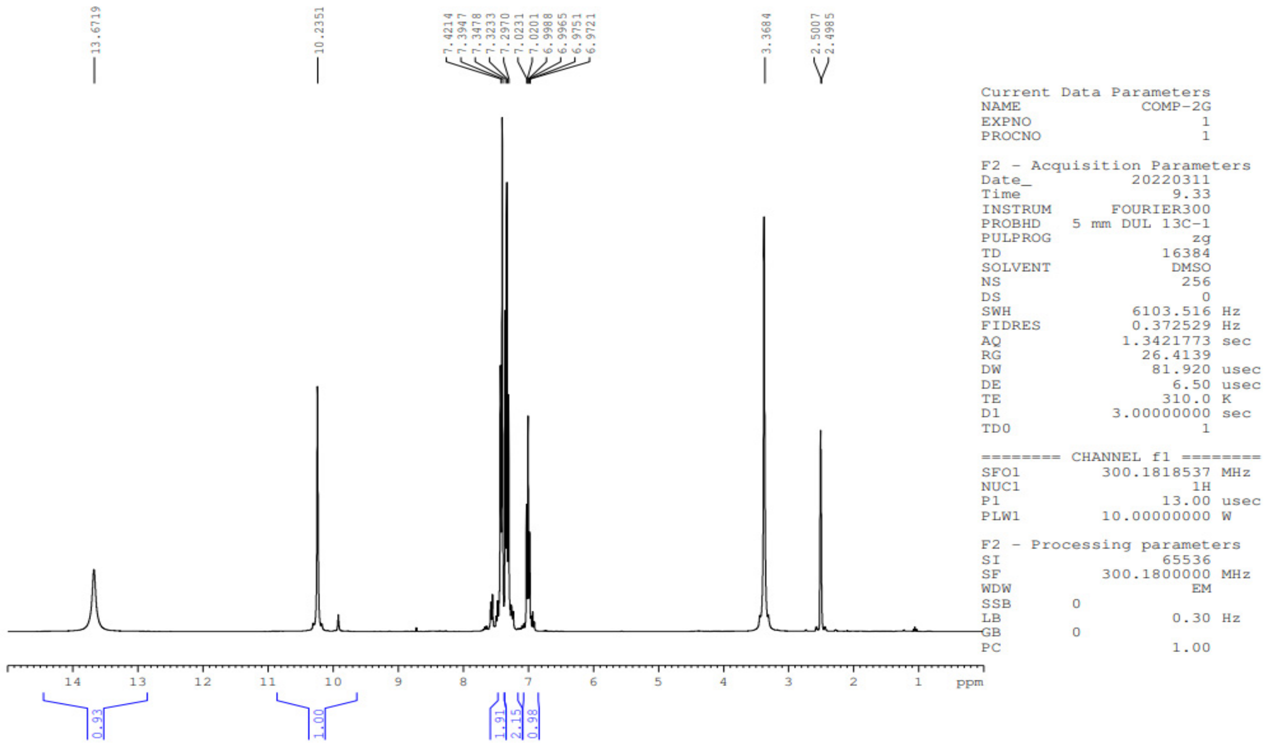

Figure S47. ^{1}**HNMR of 2h:**

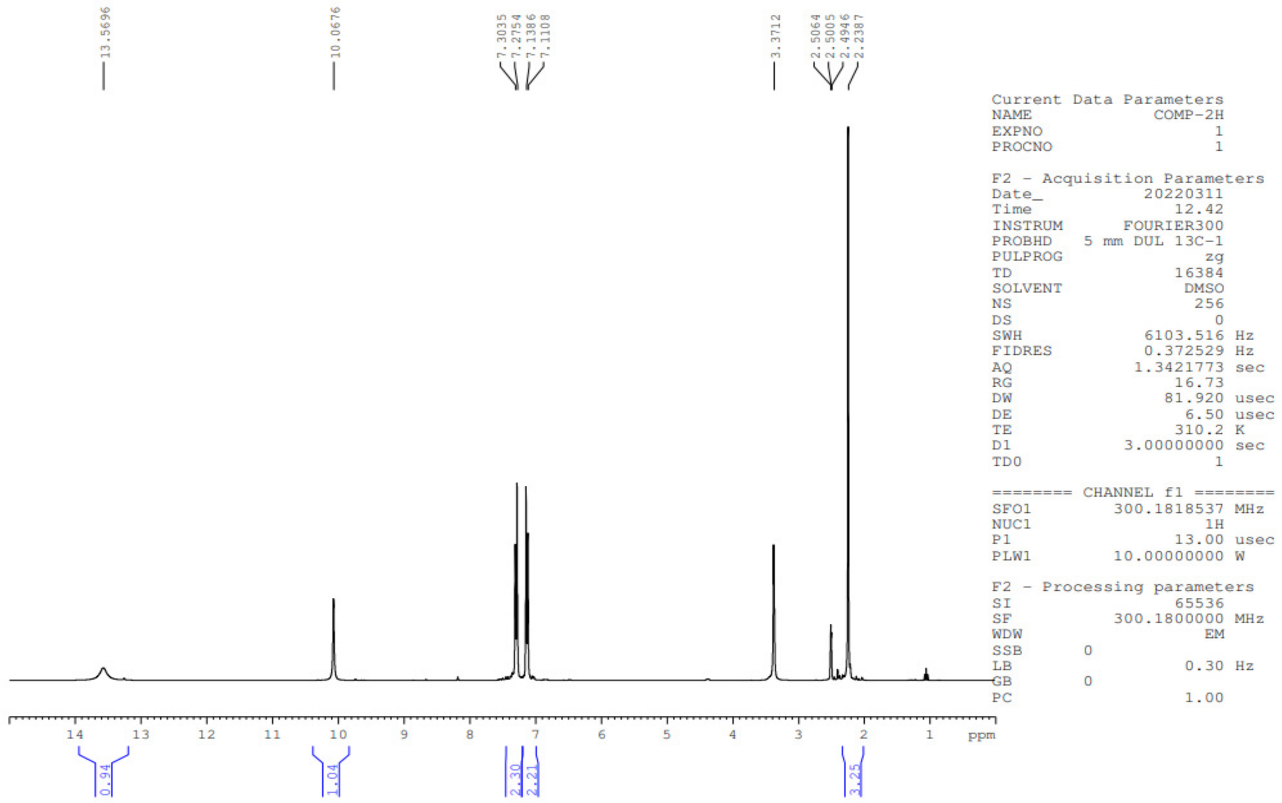

Figure S48. ^{1}**HNMR of 2i:**

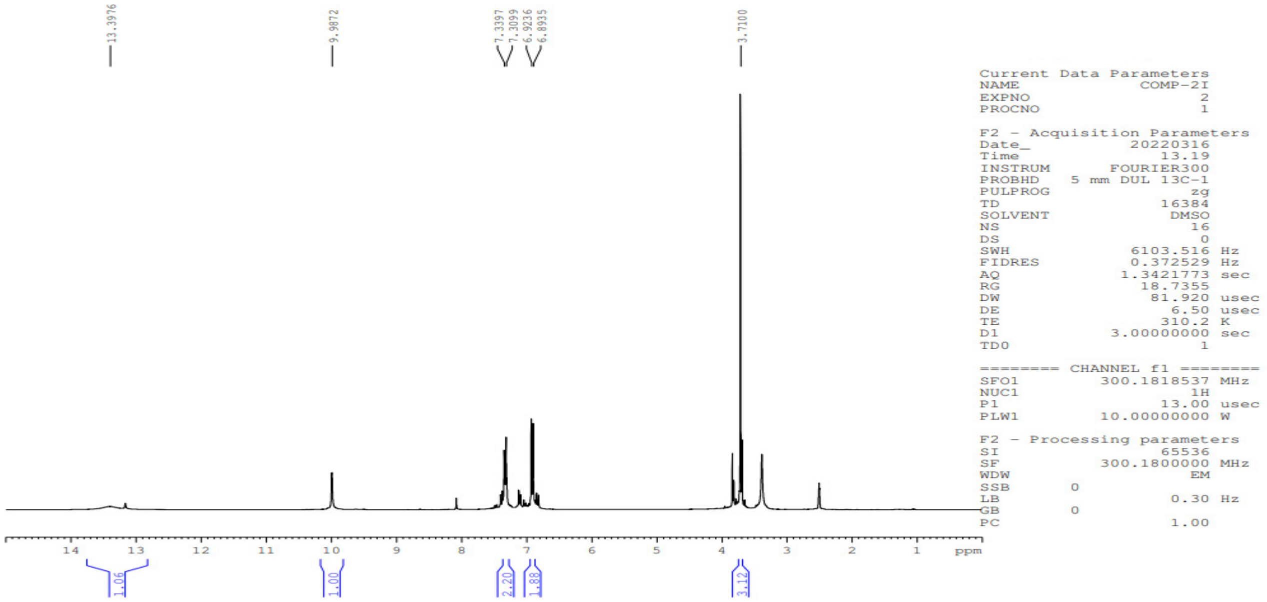

Figure S49. ^{1}HNMR of 2j:

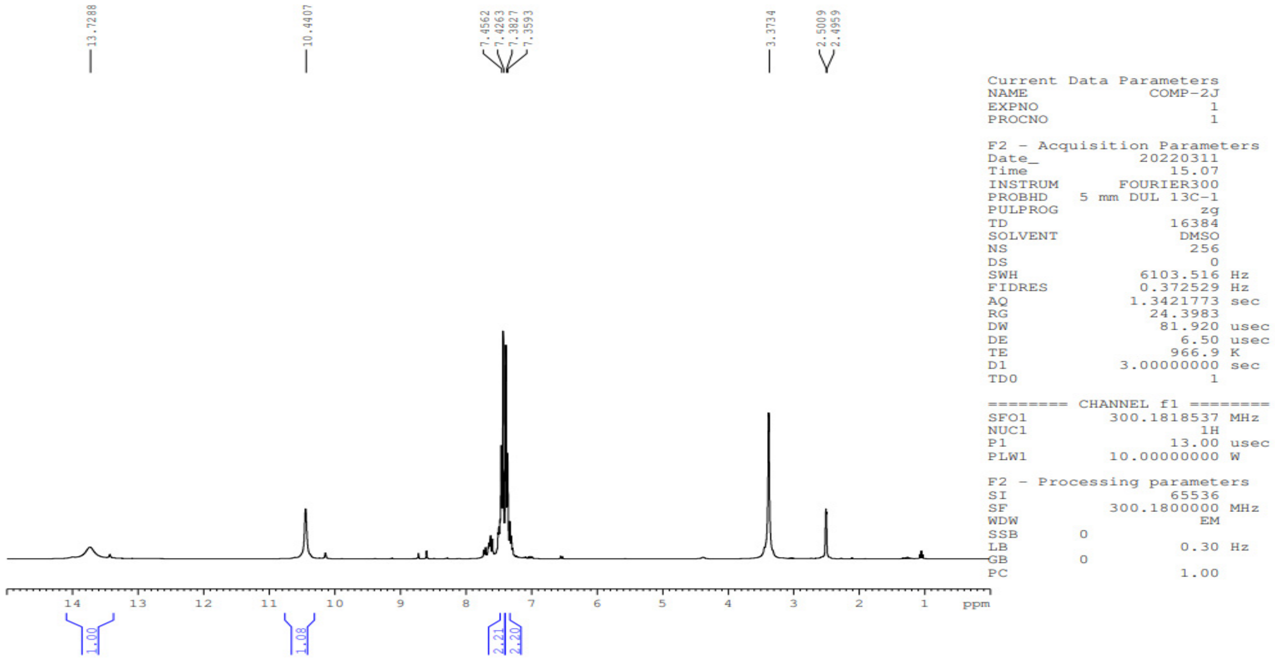

Supplement: Supplementary file 1 [file molecules-27-02121-s001.zip › molecules-1638141-supplementary.pdf]
